# Abstracts from hydrocephalus 2018: the tenth meeting of the International Society for Hydrocephalus and Cerebrospinal Fluid Disorders

**DOI:** 10.1186/s12987-018-0119-0

**Published:** 2018-12-24

## I1 Introduction

### Giorgio Palandri Hydrocephalus 2018 President

#### Consultant, Department of Neurosurgery, IRCCS Institute of Neurological Sciences of Bologna, Bellaria Hospital, Bologna, Italy

##### **Correspondence:** Giorgio Palandri: giopalandri@gmail.com

*Fluids and Barriers of the CNS* 2018, **15(Suppl 2):**I1

It has been a great privilege and honour for me and my team to host the 10th meeting of Hydrocephalus Society (International Society for Hydrocephalus and CSF Disorders) in the historic city of Bologna, Italy. This annual meeting was dedicated to research into hydrocephalus and related disorders and attracted over 300 delegates from 32 different countries.

Hydrocephalus 2018 presented a vital opportunity for clinicians and scientists to stay current on advances in the assessment, diagnosis and treatment of hydrocephalus. The delegates included many different professionals, who in very different ways and fields, deal every day with patients suffering from hydrocephalus: for example, engineers, physicists, nurses, radiologists, neurologists, neuroscientists, neurosurgeons, geriatricians, anaesthetists and neuropsychologists.

Delegates heard keynote lectures by the world’s experts on many topics including pediatric hydrocephalus, experimental hydrocephalus, normal pressure hydrocephalus, diagnosis, shunt technology, pathophysiology and brain imaging. We also had a great social program starting with a Welcome Reception at the Lamborghini Museum, followed on successive evenings by an Italian evening and a Gala dinner. Altogether, we enjoyed a very successful and exciting meeting here in Bologna.

## O1 CSF amyloid Β-related and neurofilament light proteins in the differential diagnosis of normal pressure hydrocephalus

### Samir Abu-Rumeileh^1^, Giulia Giannini^1^, Luca Albini-Riccioli^2^, Barbara Polischi^2^, David Milletti^2^, Federico Oppi^2^, Paolo Mantovani^2^, Giorgio Palandri^2^, Pietro Cortelli^1,2^, Sabina Cevoli^2^, Piero Parchi^2,3^

#### ^1^Department of Biomedical and NeuroMotor Sciences (DIBINEM), University of Bologna, Italy; ^2^IRCCS Institute of Neurological Sciences of Bologna, Bellaria Hospital, Italy; ^3^Department of Diagnostic Experimental and Specialty Medicine (DIMES), University of Bologna, Italy

##### **Correspondence**: Piero Parchi

*Fluids and Barriers of the CNS* 2018, **15(Suppl 2):**O1

**Introduction:** The diagnosis of normal pressure hydrocephalus (NPH) can be quite challenging given the phenotypic overlap with other cognitive disorders. Moreover, knowledge about the pathophysiological mechanism leading to neurological dysfunction in NPH is limited. Cerebrospinal fluid (CSF) biomarkers have been investigated to these aims, but the results appear discordant among studies.

**Methods:** We measured CSF amyloid β (Aβ)42 and Aβ40, neurofilament light chain protein (NfL), total(t)-tau and phosphorylated(p)-tau in healthy controls (n = 38) and subjects with cognitive disorders including NPH (n = 66), Alzheimer’s disease (AD) (n = 60), vascular dementia (n = 30), frontotemporal dementia spectrum (FTD) (n = 80), and dementia with Lewy bodies (n = 35).

**Results:** NPH patients showed significantly lower levels of Aβ42 (p = 0.024) and Aβ40 (p = 0.006) than controls, whereas the concentration of both t-tau and p-tau were similar between the two groups. All disease groups showed a significant increase in NfL levels (p < 0.001), with FTD patients demonstrating the highest values. Within the NPH group, NfL levels did not significantly differ between those with or without vascular and/or AD comorbidities. Since the values of Aβ42/Aβ40 ratio were significantly reduced only in AD cases, the Aβ42/Aβ40 ratio demonstrated a higher diagnostic accuracy than Aβ42 alone (AUC 0.955 ± 0.018 and 0.858 ± 0.035, respectively) in the discrimination between NPH and AD.

**Conclusion:** CSF levels of Aβ peptides and NfL may reflect two distinct pathophysiological mechanisms in NPH, namely the down-regulation of β-amyloid production and the degeneration of periventricular myelinated axons. Our data also underline the clinical value of Aβ42/Aβ40 in the differential diagnosis between NPH and AD.

## O2 The iNPH scale, DESH-score, MMSE and MRS. Efforts at associations and predictions

### Simon Agerskov^1^, Mats Tullberg^1^, Dan Farahmand^1^, Karin Sundström^1^, Carsten Wikkelsö^1^, Per Hellström^1^

#### ^1^Hydrocephalus Research Unit, Institute of Neuroscience and Physiology, Department of Clinical Neuroscience, The Sahlgrenska Academy, University of Gothenburg, Sweden

##### **Correspondence:** Simon Agerskov

*Fluids and Barriers of the CNS* 2018, **15(Suppl 2):**O2

**Introduction:** There is lack of studies on the relationship between different assessment methods in the field of iNPH. We compared the severity of symptoms in iNPH before and 3 months after shunt surgery with preoperative MRI findings, aiming to investigate the association between the iNPH scale and the DESH-score and to evaluate the ability of rated MRI findings to predict symptomatological changes following treatment.

**Methods**: INPH patients (N = 105, mean age 74, 69% male) were consecutively included. MRI scans were rated according to the scale introduced by Shinoda et al., and all patients were clinically assessed before and 3 months after surgery with the iNPH scale presented by Hellström et al. The MMSE and the mRS were also used.

**Results:** There were no significant correlations between DESH-scores and iNPH-scores (neither total nor separate domain scores) before surgery. Further, DESH-scores did not correlate significantly with changes in iNPH-scores. The iNPH scale total score was significantly correlated with preoperative MMSE and modified Rankin Scale (mRS) scores, and changes in iNPH scale scores were also associated with the changes in MMSE and mRS. No corresponding associations were found between DESH scores and these measures.

**Conclusions:** Despite the acknowledged association between DESH features and the diagnosis of iNPH, as opposed to structural findings in other neurological diseases, we found no relationship between these features and the preoperative symptom severity of the iNPH patients on the one hand, or their prognosis, on the other.

## O3 Proton spectroscopy documents deep white matter changes in shunted iNPH patients

### Luca Albini Riccioli^1^, Giorgio Palandri^2^, Sabina Cevoli^3^, David Milletti^4^, Marco Cernigliaro^5^, Alberto Conficoni^6^, Raffaele Agati^1^, Anna Federica Marliani^1^, Paolo Mantovani^2^, Giulia Giannini^3^, Pietro Cortelli^3^

#### ^1^Neuroradiology, IRCCS Istituto Scienze Neurologiche Bologna, Italy; ^2^Neurosurgery, IRCCS Istituto Scienze Neurologiche Bologna, Italy; ^3^Neurology, IRCCS Istituto Scienze Neurologiche Bologna, Italy; ^4^Rehabilitation Medicine, IRCCS Istituto Scienze Neurologiche Bologna, Italy; ^5^Radiology, Cliniche Universitarie, Università di Sassari, Italy; ^6^Cardio-Thoracic Radiology unit, Departiment of Experimental, Diagnostic and Speciality Medicine, S. Orsola-Malpighi Hospital, University of Bologna, Italy

##### **Correspondence:** Luca Albini Riccioli

*Fluids and Barriers of the CNS* 2018, **15(Suppl 2):**O3

**Introduction:** Quantitative proton magnetic resonance spectroscopy ((1) H-MRS) has been shown to be able to highlight alterations of the major metabolites in many diseases involving the deep white matter. However, very few studies in the literature have focused on its use in patients affected from idiopathic Normal Pressure Hydrocephalus (iNPH).

**Methods:** We used high field 3 Tesla (1) H-MRS to evaluate mean relative concentrations ratios for NAA, Cr, Cho and mI during the study of frontal deep white matter in a group of iNPH patients. In total, seven patients with iNPH, mean age 72 years were examined before and after shunt surgery. Relative quantitative (1) H-MR spectroscopy (3T, mean volume of interest 2.9 mL) was performed on the patients in the frontal deep white matter, and compared with healthy subjects metabolites content.

**Results:** We found a significant decrease of total *N*-acetylaspartate/creatine, total *N*-acetylaspartate/choline and an increase in myoInositol/creatine in comparison with healthy subjects in the preoperative study. No significant changes in choline values are observed, which always remains within the normal range. Spectroscopy however tend to normalize 6 months after surgery, correlating with clinical improvement.

**Conclusions:** In this small sample, (1) H-MRS seems to highlight an alteration of the relationships between the main metabolites in iNPH patients compared to the healthy patients and, after the intervention, seems to show a tendency to normalization correlated with an overall improvement of the clinical status.

## O4 Multidisciplinary evaluation of NPH at Uppsala University Hospital

### Christian Andersson^1^, Agneta Gustafsson^1^, Maria Ekblom^1^, Johan Virhammar^1^, Madelene Braun^1^, Lars Stridh^1^, Dag Nilsson^1^, Kristina G Cesarini^2^

#### ^1^Department of Neuroscience, Neurology, Uppsala University Hospital, Sweden; ^2^Department of Neuroscience, Neurosurgery, Uppsala University Hospital, Sweden

##### **Correspondence:** Christian Andersson

*Fluids and Barriers of the CNS* 2018, **15(Suppl 2):**O4

**Introduction:** A multidisciplinary NPH team is since 2003 operating at the University Hospital of Uppsala, investigating and treating patients with suspected NPH. A NPH evaluation aims to assess a correct diagnosis, but also to select candidates for shunt surgery.

**Methods:** The Uppsala NPH-team includes neurologists, neurosurgeons, occupational therapists (OT), physiotherapists (PT), a nurse and nurse assistant, an engineer and a secretary for administrative support. The investigation set up for all patients includes neuroimaging with MRI, CSF-dynamic test, CSF tap test and CSF-biomarkers for dementia. Symptoms are assessed with the Hellström iNPH-scale and each patient are investigated by neurologists, OT and PT. At a weekly multidisciplinary team conference, the members present their test results, a diagnosis is suggested, and it is decided whether the patient should be recommended shunt surgery. A thorough control of preoperative risk factors is done. Follow-up with repeated evaluation is performed at 3 and 12 months after surgery.

**Result:** During 2017 the NPH team evaluated 154 patients. 127 first time investigations for NPH, 24 were investigated for possible shunt dysfunction, three patients with idiopathic intracranial hypertension. 54 new shunts were implanted and 17 revised due to malfunction.

**Conclusion:** NPH is a disease with symptoms that overlap with other neurodegenerative disorders. Correct diagnosis and selection of suitable shunt surgery candidates are of great benefit for the group of patients and positive in a social-economic perspective. A multidisciplinary team with a standardized protocol allows reproducible evaluations of symptoms, aiding the assessment of outcome and complications of shunt surgery.

## O5 The association between self-reported INPH symptoms and findings on clinical examination

### Johanna Andersson^1^, Katrina Fors^1^, Lars Söderström^2^, Katarina Laurell^1^

#### ^1^Department of Pharmacology and Clinical Neuroscience, Umeå University, Sweden; ^2^Unit of Research, Development and Education, Östersund Hospital, Region Jämtland Härjedalen, Sweden

##### **Correspondence:** Johanna Andersson

*Fluids and Barriers of the CNS* 2018, **15(Suppl 2):**O5

**Background/Objective:** Questionnaires that make it easy to identify elderly with symptoms of idiopathic normal pressure hydrocephalus (iNPH) are needed in larger scaled epidemiological studies. The aim of this study was to evaluate whether self-reported symptoms in a questionnaire could be verified by objective findings on clinical tests.

**Methods:** A total of 1000 individuals, 65 years and older, randomly selected from the Swedish population registry, received a questionnaire with seven “yes” or “no” questions on iNPH symptoms. The response rate was 67%. Based on the answers, a subgroup (n = 168) underwent clinical examination and a CTscan of the brain. Hellström’s iNPH scale, with the four domains of neuropsychology, gait, continence and balance, was used to assess the severity of symptoms.

**Results:** The number of symptoms reported in the questionnaire correlated with the severity of symptoms at the clinical examination (r_s_ = − 0.6, p < 0.001). The concordance between symptoms reported in the questionnaire and observed at the clinical examination, was highest for gait and continence (AUC = 0.75 and 0.86 respectively, p < 0.001) and lowest for neuropsychology (AUC = 0.6, p = 0.25).

**Conclusion:** The questionnaire seems to be a feasible screening method for iNPH symptoms, especially for gait impairment and incontinence.

## O6 Survival in idiopathic normal pressure hydrocephalus

### Kerstin Andrén^1^, Carsten Wikkelsö^1^, Nina Sundström^2^, Simon Agerskov^1^, Hanna Israelsson^3^, Katarina Laurell^3^, Per Hellström^1^, Mats Tullberg^1^

#### ^1^Hydrocephalus Research Unit, Institute of Neuroscience and Physiology, Department of Clinical Neuroscience, The Sahlgrenska Academy, University of Gothenburg, Sweden; ^2^Department of Radiation Sciences, Biomedical Engineering, Umeå University, Sweden; ^3^Department of Pharmacology and Clinical Neuroscience, Umeå University, Sweden

##### **Correspondence:** Kerstin Andrén

*Fluids and Barriers of the CNS* 2018, **15(Suppl 2):**O6

**Introduction:** In treated patients with idiopathic Normal Pressure Hydrocephalus (iNPH), mortality has been described to be 2.5 to 3.3 times increased compared to the general population. It is unknown whether a positive treatment effect influences survival and data on the influence of vascular comorbidities on survival is scarce.

**Methods:** We included 979 iNPH patients from the Swedish Hydrocephalus Quality Registry, SHQR. A matched control group (n = 4890) was defined from the general population by Statistics Sweden. Dates of death were obtained from the National Board of Health and Welfare’s Cause of Death Register. Hazard ratios, HR, were calculated by Cox proportional hazards models.

**Results:** Thirty-seven % of the patients and 23% of controls died during the (median) 6 year study period. HR for iNPH patients compared to controls, was 1.81, 95% CI 1.61–2.04, p < 0.001. A multivariable analysis was performed within the cohort of iNPH patients: significant covariates were age, HR 1.07/year, 95% CI 1.05–1.09, p < 0.001 and heart disease, HR 1.30, 95% CI 1.01–1.68, p = 0.044. Patients who were improved in mRS (≥ 1 step, n = 244) at 3 months after surgery survived longer than patients who were unchanged or deteriorated (n = 295 and n = 85), HR 0.55, 95% CI 0.41–0.75, p < 0.001. The prevalence of vascular comorbidities was similar for improved as for non-improved patients.

**Conclusion:** Mortality in iNPH patients was increased by 1.81 compared to controls; a lower figure than in earlier studies. Patients who were improved in mRS 3 months after surgery had a better survival; this was not explained by any difference in vascular comorbidity.

## O7 Neuroendoscopic procedures in the treatment of compartmentalized hydrocephalus in children

### Elena Arcovio^1^, Flavio Giordano^1^, Barbara Spacca^1^, Regina Mura^1^, Federico Mussa^1^, Mirko Scagnet^1^, Manuela Grandoni^1^, Pierarturo Donati^1^, Lorenzo Genitori^1^

#### ^1^Department of Neurosurgery, Anna Meyer Hospital, Firenze, Italy

##### **Correspondence:** Elena Arcovio

*Fluids and Barriers of the CNS* 2018, **15(Suppl 2):**O7

**Introduction:** Compartmentalized hydrocephalus (CH), also known as complex or multiloculated hydrocephalus, is a rare disease usually observed in the pediatric age. It is characterized by presence of abnormal septa ad/or obstruction that may lead to isolated compartments inside the ventricular system. CH may be congenital but is usually due to infection, hemorrhage or shunt surgery complication. It may be classified in: univentricular hydrocephalus, multicystic hydrocephalus, trapped fourth ventricle, trapped temporal ventricle.

**Materials and methods:** Since 1994 to 2017 sixty-four patients affected by symptomatic CH due to various pathologies underwent to different neuroendoscopic procedures: septostomy and Monro foraminoplasty, endoscopic third ventriculostomy (ETV), acqueductoplasty, multiple fenestrations, ventricular catheter lysis, choroid plexus coagulation. More than half children (49/64; 76.6%) have been previously operated for VP-shunt insertion (secondary CH), while 15 out 64 (23.4%) received neuroendoscopic procedures as first choice surgery (primitive CH). Surgical outcomes were evaluated according to number of patients shunt-free, number of shunt revisions and number of VP-shunt per patient.

**Results:** Sixty-four patients underwent to a total number of 114 neuroendoscopic procedures for CH. There were 36 males, 28 females; mean age was 11.1 years (range 1–15 years). Surgeries were: septostomy and Monro foraminoplasty (27), ETV (26) and redo-ETV (14), acqueductoplasty (22), multiple septa and cyst fenestration (20), ventricular catheter lysis (3), choroid plexus coagulation (2).

Mean follow up was 61.8 months (range 9 months–18.3 years). All of them received a full neuroradiological work-up by means of CISS/CSF-drive MR both before and after surgery.

Surgical mortality occurred in one patient (0.6%) with CH due to myelomeningocele. Morbidity accounted for 26.5% (17/64) because of infection (2/114; 1.7%), CSF-leakage (5/114; 4.4%), cranial nerve palsy (4/114; 3.5%), intraventricular hemorrhage (2/114; 1.7%). Four procedures (3.5%) were aborted because of distorted anatomy.

At last follow-up 9 out of 34 patients carrying only one shunt before were shunt-free after surgery (26.5%) corresponding to an overall 14.2% of shunt-free children in the whole series. 70% (44/63) patients maintained only one shunt while 10/63 (16%) two or more after surgery. The average number of shunt revision after surgery was reduced from 2.82 to 1.51.

**Conclusions:** Neuroendoscopic procedures are efficacious in the management of primary and secondary CH, and may successfully reduce both the number of shunt revisions and the number of VP-shunt per patient. ETV should be considered as first choice for shunt failure everytime the anatomy of hydrocephalus allows it.

## O8 Codman certas, certas-plus and siphonguard

### Alfred Aschoff

#### Department of Neurosurgery, Formerly University of Heidelberg, Germany

##### **Correspondence:** Alfred Aschoff

*Fluids and Barriers of the CNS* 2018, **15(Suppl 2):**O8

**Introduction:** In 2011 the Codman-Certas was launched, in 2013 recalled and in 2015 relaunched. We found in PubMed with Certas 8, CertasPlus 1 and SiphonGuard (SG) 9 papers only.

**Methods**: We tested in vitro 10 SGs, but only could work with demo-probes of Certas- or CertasPlus-valves. 5 patents 2011–2014 were analysed exhaustively, in addition all company-data and publications.

**Results:** In contrast to the excellent precision of adjustable Medos (± 10 mmH_2_O!) Certas showed meagre tolerances of ± 20 to ± 50 mm. Eklund measured (2012) in range 400 mmH_2_O deviations of 51–156 mmH_2_O. The 18-stepped Medos allows a subtile fine regulation. Certas has only seven steps, 3 of those in the seldom used high pressure (145–215 mm). During puncture the tip of needle can penetrate the valve mechanism. With strong magnets (ProSA) it is possible to elevate the rotor and to adjust every position. Using an iPhone 5S the mechanisme can be switched-off (Ozturk 2017). The SG with 8–15 cmH_2_O pressure allows flow up to 150 mL/h, leading to a high quote of chronic subdurals (19.3%, Sundström 2017); between 25 and 50 cmH_2_O the flow is too low. The helictical bottleneck has a diameter of 0.4 mm, ergo is at risk of underdrainage.

**Conclusions**: Certas/Plus has solved the Medos-problems of X-rays and readjustments after MRIs. The price is a massive deterioration of precision, fine tuning and safety (missing needle guard). The risk of cSDH in NPH-patients is with Certas 16.7% and with SG with adjustable Medos 19.6% (Sundström 2017).

## O9 Radiographic markers of disturbances in CSF dynamics: correlating imaging with 24-h ICP monitoring

### Hasan Asif^1^, Claudia L Craven^1^, Lewis T Thorne^1^, Laurence D Watkins^1^, Ahmed K Toma^1^

#### ^1^National Hospital for Neurology and Neurosurgery, Queen Square, London, United Kingdom

##### **Correspondence:** Hasan Asif

*Fluids and Barriers of the CNS* 2018, **15(Suppl 2):**O9

**Introduction:** Disorders with chronically elevated ICP have salient imaging findings associated with the sella turcica and optic nerves, however these can be incidental. Patients in this centre with suspected pressure disturbances undergo 24-h ICP monitoring. We aim to quantify the degree of correlation between imaging features and ICP.

**Methods:** T1-saggital views for sella volume, optic nerve vertical tortuosity, then T2-axial views for optic nerve sheath distension were reviewed against respective median ICP and pulse amplitudes (PA). Imaging data was blindly collected, with triple reviews for discordant values.

**Results:** One-hundred and 26 patients (35M:91F) with suspected/established disorders of CSF dynamics underwent ICPM with recent MR imaging.

The mean ICP of four sella morphologies (full/flat/concave/empty) were 1.17, 4.80, 8.36 and 16.7 mmHg respectively (p < 0.01). AUROC for sella morphology predicting ICP was 0.81. This measurement was able to detect minimum ICP of 5.30 mmHg with 73.0% sensitivity and specificity, 73.0% PPV and 69.8% NPV.

The mean PA values were 3.97, 5.22, 6.06 and 9.61 mmHg respectively (p < 0.01). AUROC for sella morphology predicting PA was 0.78. This measurement was able to detect minimum PA of 5.47 mmHg with 76.3% sensitivity, 79.5% specificity, 63.5% PPV and 81.0% NPV.

Mean ICP values for vertical tortuosity (none/unilateral/bilateral) were 4.65, 7.81 and 13.5 mmHg respectively (ns). Mean PA values were 5.20, 7.14 and 6.98 mmHg respectively (p < 0.05).

Mean ICP values for rail tracking (none/unilateral/bilateral) were 4.52, 7.54 and 15.7 mmHg respectively (p < 0.01). Mean PA values were 5.18, 5.84 and 8.04 mmHg respectively (p < 0.0001).

**Conclusion:** Combined radiological features of ICP are promising non-invasive markers for raised ICP.

## O10 Long-term outcomes of venous sinus stents in idiopathic intracranial hypertension

### Hasan Asif^1^, Claudia L Craven^1^, Lewis T Thorne^1^, Laurence D Watkins^1^, Ahmed K Toma^1^

#### ^1^National Hospital for Neurology and Neurosurgery, Queen Square, London, United Kingdom

##### **Correspondence:** Hasan Asif

*Fluids and Barriers of the CNS* 2018, **15(Suppl 2):**O10

**Introduction:** Idiopathic intracranial hypertension (IIH) is associated with dural venous sinus stenosis (DVSS). This finding is increasingly treated with endovascular insertion of stents. Clinical and manometric improvements after stent placement have been described. However, there is little data reporting further need for CSF diversion, complication rates and sustained improvements in ICP.

**Methods:** Single centre case series. Clinical notes, radiographic reports and 24-h ICP monitoring data before and after stent placement was collected.

**Results:** Between 2010 and 2015, 24 patients underwent stent insertion as a primary interventional procedure for IIH on discovery of DVSS with medical management ongoing. Over follow-up of 1089.2 ± 107.1 days, six patients remained symptomatic and went onto require CSF diversion (one initially had an LP shunt later changed to a VPleural shunt, one had a VP shunt and four had LP shunts), 75.0% did not require CSF diversion.

One patient developed early stent thrombosis requiring VKA anticoagulation for 3-months, this patient also developed a new stenosis proximal to the stent at 2 years. A second patient developed in-stent stenosis requiring balloon angioplasty at 2 years and subsequent repeat stenting at 3 years.

Eleven patients had 24-h ICP monitoring at baseline and a mean of 231.9 ± 129.5 days after DVSS stent placement. The mean reduction in ICP was 7.92 ± 1.80 mmHg (p < 0.01) and PA was 2.84 ± 0.84 mmHg (p < 0.01).

**Conclusion:** DVSS stenting is a viable endovascular therapy for IIH with modest long-term patency and ICP reduction. However, a quarter of stented patients required subsequent CSF diversion to manage their symptoms.

## O11 Ultrasound-measured optic nerve sheath diameter in suspected idiopathic normal-pressure hydrocephalus patients pre and post high volume spinal tap test

### Raffaele Aspide^1^, Luca Albini Riccioli^1^, Maria Chiara Casadio^1^, Giorgio Palandri^1^, Paolo Mantovani^3^, Pietro Cortelli^1^

#### ^1^IRCCS Istituto Scienze Neurologiche Bologna, Anesthesia-Neurointensive care, Bologna, Italy; ^2^IRCCS Istituto Scienze Neurologiche Bologna, Neuroradiology, Bologna, Italy; ^3^IRCCS Istituto Scienze Neurologiche Bologna, Neurosurgery, Bologna, Italy; ^4^DIBIEM, Università di Bologna, Italy, 40139

##### **Correspondence:** Raffaele Aspide

*Fluids and Barriers of the CNS* 2018, **15(Suppl 2):**O11

**Introduction:** Increase in optic nerve sheath diameter (ONSD) in trans-orbital sonography has been proven to be able to detect elevated intracranial pressure (ICP) in different scenarios, but little is known of conditions without increased ICP. From 2015 patients with a suspect of idiopathic Normal Pressure Hydrocephalus (iNPH) were prospectively evaluated by a multidisciplinary team at the Institute of Neurological Sciences of Bologna. In selected patients, we included the measurement of ONSD with MRI and ultrasound (US) technique, to identify a possible new follow-up modality after high volume spinal tap test (TT) and after ventriculo-peritoneal shunt (VPS).

**Methods:** An experienced neuroradiologist measured the vertical and horizontal diameters of optic nerve sheath (ONS) bilaterally, calculated on a plane orthogonal to the optic nerve, in a three dimensional T2 weighted MRI sequence. After an informed consent of each patient was obtained, in a cohort of 13 patients with suspect of iNPH, the ONS cranio-caudal (CC) and latero-lateral (LL) diameters have been measured at landmark (ophthalmic artery-optic nerve cross point, 3 mm behind the optical disk), by another expert performer, in a blind mode, using US, 24 h before and after spinal TT and even after possible VPS (88 measurements). A high-frequency linear probe 7.5 MHz by an ultrasound specially set machine, has been used.

**Results:** The examined cohort had an average of 74 (SD ± 2) years, the median value of Evan’s index was 0.36 (IQR 0.31–0.37), the callosal angle (CA) median value was 70° (IQR 53–80). Analyzing CC and LL ONSD pre-tap test values, we found a weak concordance between MRI and US measurements (range 0.139–0.372), using Spearman’s rho and Bland–Altman plots (best value p 0.05 LL ONSD left eye). In general, US seems to overestimate ONSD with an average of 0.5 mm. The ONSD median values of the two eyes’ sum, in this cohort of patients, with the two different measurement technics are: 6.4 mm [IQR 6.1–6.7] for US and 5.9 mm [5.3–6.5] for MRI, higher than normal values reported by recent literature (3.6–5.1 mm). Comparing pre and post spinal TT ONSD, there aren’t any significant variations (best value p 0.3 LL ONSD left eye). There is no correlation between ONSD (MRI/US) versus Evan’s index (p 0.379) and CA (p 0.158). On average, patients with iNPH have larger ONS diameters.

**Conclusions:** CA and Evan’s index seem to be a good predictive and diagnostic tool, the ONSD could strengthen the arsenal available for iPNH patients. ONS diameters must be verified after spinal TT and VPS, but MRI is not easily repeatable. It is therefore necessary to refine the US technique to obtain more precise measurements. The next step of this study is to collect a correct sample size to validate this method and find correlation with CA and Evan’s index, to contribute to the literature’s debate on compliance of cerebral ventricles in iNPH, that is still very controversial.

## O12 Refractory low-pressure hydrocephalus associated with hemangioblastomatosis managed with improvised negative-pressure shunt

### Abdul Badran^1^, Matthew J. Shepard^1,2^, Alexander Ksendzovsky^1,2^, Roger Murayi^1^, Christina Hayes^1^, DeeDee Smart^1^, Prashant Chittiboina^1^

#### ^1^Surgical Neurology Branch, National Institute of Neurological Disorders and Stroke, Bethesda, Maryland, United States; ^2^Department of Neurologic Surgery, University of Virginia Health System, Charlottesville, United States

##### **Correspondence:** Abdul Badran

*Fluids and Barriers of the CNS* 2018, **15(Suppl 2):**O12

**Introduction:** Low-Pressure Hydrocephalus (LPH) is a rare clinical diagnosis, characterized by neurologic decline and ventriculomegaly that persist despite normal to low intracranial pressure. LPH is typically managed by negative-pressure drainage techniques.

**Methods:** We present the case of a middle-aged man with a history of hemangioblastomatosis who developed LPH which was managed using an improvised ultra-low pressure valveless ventriculoperitoneal shunt created using off-the-shelf components. The following steps were taken to create the shunt: (1) Ligation of the open terminus of a distal peritoneal catheter using 2–0 suture to prevent reflux and allow sub-zero pressure drainage via the distal slit valves, facilitating siphoning; (2) Division and attachment of the ventricular aspect of a ventricular catheter to a convertible reservoir; (3) Division of the peritoneal aspect of a convertible reservoir and connection to the proximal end of the peritoneal catheter using suture.

**Results:** Our patient initially presented to us after spontaneous subarachnoid hemorrhage associated with hemangioblastomatosis. He was treated with a ventriculoperitoneal shunt and underwent resection of a Meckel’s cave hemangioblastoma and whole brain irradiation. One month later, he presented to us again with worsening headaches and persistent hydrocephalus despite shunt interrogations and revisions revealing no malfunction. Ventriculostomy drainage at negative-pressure was required for resolution of symptoms and ventriculomegaly, leading us to a diagnosis of LPH. After insertion of the improvised negative-pressure shunt there has been maintained resolution for over 1 year.

**Conclusions:** LPH can be successfully treated using a simple improvised negative-pressure ventriculoperitoneal shunt.

Informed consent has been obtained from the patient.

## O13 Intravoxel incoherent motion (IVIM) MRI in patients affected by probable idiopathic normal pressure hydrocephalus (INPH): a preliminary study

### Daniele Bagatto^2^, Francesco Tuniz^1^, Maria Cristina De Colle^2^, Daniela Drigo^2^, Marta Maieron^3^, Maria Caterina Vescovi^1^, Enrico Belgrado^4^, Miran Skrap^1^

#### ^1^Neurosurgery Department, ASUI S.M. della Misericordia, Udine, Italy; ^2^Neuroradiology Department, ASUI S.M. della Misericordia, Udine, Italy; ^3^Physics Department, ASUI S.M. della Misericordia, Udine, Italy; ^4^Neurology Department, ASUI S.M. della Misericordia, Udine, Italy

##### **Correspondence:** Daniele Bagatto

*Fluids and Barriers of the CNS* 2018, **15(Suppl 2):**O13

**Introduction:** Intravoxel incoherent motion (IVIM) is a technique which can measure diffusion of water molecules and blood perfusion volume in microvasculature, therefore can provide both information of blood flow and blood perfusion. Aim of the study was to compare IVIM-MRI derived parameters: slow diffusion coefficient (D), fast diffusion coefficient (D*) and perfusion fraction (*f*) among a group of patients affected by probable iNPH.

**Methods:** A total of 12 patients with diagnosis of probable iNPH were submitted to a diagnostic MR examination including an IVIM multi *b*-value sequence on a 3.0 Tesla-Magnet before and immediately after a lumbar-infusion and a Tap-test. Seven subjects were positive to invasive tests (group 1) while five patients were negative (group 2). For each patient the IVIM multi *b*-value sequence was post-processed using a specific software (Olea Sphere, France). IVIM derived parameters D, D* and *f* were calculated from 20 different regions of interest (ROIs) that were placed in the basal ganglia region and in the periventricular-white-matter (PVWM) of both hemispheres. Comparison between D, D* and *f* distribution among the two different groups of patients and correlation with invasive test results was performed with non-parametric-Wilcoxon–Mann–Whitney-test.

**Results:** Relevant differences although not yet significant were found in the distribution of D, D* and *f* in the basal ganglia region or in the PVWM among the two different groups of patients, both D* and *f* were lower in group 1 patients especially at basal evaluation.

**Conclusions:** IVIM-MRI could be a promising non-invasive technique in the evaluation of patients affected by probable iNPH.

## O14 To investigate cerebral blood flow by MRI

### Olivier Balédent^1,2^, Pan Liu^1^, Armelle Lokossou^1^, Serge Metanbou^2^, Cyrille Capel^1,2^, Malek Makki^1,2^

#### ^1^Chimère 7516, University of Picardie Jules Verne, Amiens, France; ^2^University Hospital, Amiens, Picardie, France

##### **Correspondence:** Olivier Balédent

*Fluids and Barriers of the CNS* 2018, **15(Suppl 2):**O14

**Introduction:** Phase Contrast sequence (PCMRI) is the unique technique to quantify in around 1-min blood and CSF in the cranium to provide a mean flow curve of the cardiac cycle. Using new MRI development (EPI-PCMRI) it is now possible to quantify flows curve in real-time! What is the EPI-PCMRI contribution to investigate cerebral blood flow?

**Methods:** Cerebral blood flows of five hydrocephalus patients were investigated in a 3T MRI. Internal carotid, vertebral and jugular vein vessels were explored by PCMRI with a good spatial resolution, to acquire only one mean curve accurate flow of 32 points from the 2 min acquisition time. Epi-PCMRI was also done with a low spatial resolution but able to quantify directly flow every 0.1 s during 1 min. A dedicated post processing software quickly calculated all the key flow parameters. A spearman correlation test evaluated EPI-PCMRI in front of PCMRI.

Frequential analysis evaluated respiratory component in the flows.

**Results:** Blood flows were calculated in nine internal carotids arteries, six vertebral arteries and four jugular veins by the two techniques. The spearman correlation was pretty good (r = 0.94, p < 10–5). the Blood flow shape changed. Respiratory frequency amplitude was present in all the flows, its impact was more important in the jugular flows.

**Conclusions:** By combining flow accuracy and high spatial resolution from PCMRI with the high temporal of EPI-PCMRI we have shown that arterial and venous blood flow change due to breathing. These new investigations applied to CSF should help to better understand the hydrocephalus mechanism.

## O15 Do we correctly manage urinary symptoms in idiopathic normal pressure hydrocephalus?

### Elsa Bey^1^, Olivier Casez^2^, Loic Le Normand^3^, Romain Manet^4^

#### ^1^Department of Urology, Hôpital Nord, University of Grenoble, France; ^2^Department of Neurology, Hôpital Nord, University of Grenoble, France; ^3^Department of Urology, Hôtel Dieu, University of Nantes, France; ^4^Department of Neurosurgery B, Hôpital neurologique Wertheimer, University of Lyon, France

##### **Correspondence:** Elsa Bey

*Fluids and Barriers of the CNS* 2018, **15(Suppl 2):**O14

**Introduction:** Urinary symptoms in idiopathic normal pressure hydrocephalus (iNPH) has received little attention from the scientific and medical community.

**Materials and methods:** A literature review of MedLine publications concerning urinary incontinence in iNPH was conducted, including prospective and retrospective studies as well as previous reviews.

**Results:** *Pathophysiology*: Urinary symptoms in iNPH are mainly represented by overactive bladder, explained by a dysregulation of the spino-bulbo-spinal micturition reflex, in relation with a right frontal hypoperfusion. Isolated overactive bladder is more frequent (64%) than urinary incontinence (57%). Detrusor overactivity is seen in 95.2% of the cases. *Assessment*: The International Society for Hydrocephalus and CSF Disorders recommends to realize a micturition calendar and a bladder ultrasonography to measure post void residual to all patients presenting iNPH. The interest of urodynamics analysis in this situation is considered low. *Efficacy of surgical treatment*: shunt surgery is efficient on urinary symptoms in 61.5% of the patients. This recovery could result from an increased perfusion and a functional restoration of the mid-cingulate, that normally inhibits the micturition reflex. Overall, this clinical improvement is considered higher than on the cognition aspect but lower than on gait disturbance. *Medical options*, added or not to surgery, they include specific anticholinergic drugs that do not pass through the blood–brain barrier, transcutaneous electrical nerve stimulation and sacral neuromodulation.

**Conclusion:** This article highlights the importance of a harmonization of neuro-urological practices in the pre-therapeutic and follow-up evaluations of patients suffering from iNPH.

## O16 Cognit automated computerized neuropsychological test battery is responsive to cognitive change after shunt surgery in INPH

### Anders Behrens^1,2^, Eva Elgh^3^, Göran Leijon^4^, Bo Kristensen^5^, Anders Eklund^6,7^, Jan Malm^2^

#### ^1^Department of Neurology, Blekinge Hospital, Karlskrona, Sweden; ^2^Department of Pharmacology and Clinical Neuroscience, Umeå University, Sweden; ^3^Department of Psychology, Umeå University, Sweden; ^4^Department of Clinical and Experimental Medicine, Linköping University Hospital, Sweden; ^5^Department of Neurology, Aalborg University Hospital, Denmark; ^6^Centre for Biomedical Engineering and Physics, Umeå University, Sweden; ^7^Department of Radiation Science, Umeå University, Sweden

##### **Correspondence:** Anders Behrens

*Fluids and Barriers of the CNS* 2018, **15(Suppl 2):**O15

**Introduction:** The Computerized General Neuropsychological INPH Test (CoGNIT), has been developed for standardized and accessible cognitive assessments in patients with Idiopathic Normal Pressure Hydrocephalus (INPH). Tests are self-administered with animated instructions, can be administered by a nurse and include tests of memory, executive functions, attention, manual dexterity and psychomotor speed. The aim of this study was to evaluate CoGNIT’s responsiveness to cognitive change after shunt surgery in INPH patients.

**Methods:** CoGNIT was administered to 41 INPH patients preoperatively and at a postoperative follow up 4 months after shunt surgery. Scores were compared to those of 44 healthy elderly.

**Results:** Improvement after shunt surgery was seen in all administered cognitive domains: attention (Two choice reaction test, p < 0.01), executive functions (Stroop incongruent test, p < 0.01), memory (Ten-word list test, p < 0.01), psychomotor speed (Stroop congruent test, p < 0.01) and manual dexterity (Four-finger tapping, p < 0.01). A composite score showed improvement in 56% of the patients. Preoperative INPH test scores were significantly impaired compared to healthy elderly (P < 0.001 for all tests).

**Conclusions:** CoGNIT is sensitive to cognitive impairment at baseline, and responsive to postoperative improvement in INPH patients. CoGNIT is a practical, self-administrated, validated computerized test battery with a potential to be useful in the cognitive assessments of INPH patients in both clinic and research.

## O17 Impact of the cranial and spinal compliance on CSF hydrodynamics regarding normal pressure hydrocephalus

### Anne Benninghaus^1^, Armell Lokossou^2^, Olivier Balédent^2^, Steffen Leonhardt^3^, Klaus Radermacher^1^

#### ^1^Chair of Medical Engineering, RWTH Aachen University, Germany; ^2^BioFlow Image Processing, University Hospital of Picardy Jules Verne, Amiens, France; ^3^Philips Chair for Medical Information Technology, RWTH Aachen University, Germany

##### **Correspondence:** Anne Benninghaus

*Fluids and Barriers of the CNS* 2018, **15(Suppl 2):**O16

**Introduction:** The etiology of Normal Pressure Hydrocephalus (NPH) is still not clear. However, it is known, that a reduced compliance of the craniospinal system could be one key factor explaining typical NPH symptoms such as high intracranial pressure (ICP) amplitudes and a decreased cerebrospinal fluid (CSF) flow in the spinal canal. Nonetheless, so far, the impact of the cranial or spinal compliance is on the fluid dynamics is still unclear.

**Methods:** An in vitro model of the craniospinal system, including ventricles in a parenchyma, a cranial subarachnoid space connected to a first compliance chamber and a spinal canal including a second compliance, was used to investigate the impact of cranial or spinal compliance respectively. The ICP and the spinal CSF flow were measured.

**Results:** The reduction of the compliance from 1.14 to 0.36 mL/mmHg increased the ICP amplitudes 11.6%, regardless the regional distribution (cranial/spinal). However, a pathological CSF flow (decreased compared with PC-MRI flow data) could only be observed when reducing the spinal compliance to less than 1.89 mL/mmHg. A reduction in cranial compliance had the opposite effect.

**Conclusions:** NPH patients with a reduced spinal CSF flow are likely to have a reduced spinal compliance. Whereas increased ICP amplitudes result from a decreased overall compliance. However, the study only investigates static compliances. Implementing a dynamic (time-dependent) compliance is a major objective of our ongoing research.

## O18 Preclinical testing of pharmaceutical treatments for hydrocephalus

### Bonnie Blazer-Yost^1^, Daniel Preston^1^, Stefanie Simpson^1^, Alexandra Hochstetler^1^

#### ^1^Department of Biology, Indiana University Purdue University Indianapolis, Unites States

##### **Correspondence:** Bonnie Blazer-Yost

*Fluids and Barriers of the CNS* 2018, **15(Suppl 2):**O17

**Introduction:** Effective pharmaceutical treatments for hydrocephalus represent a serious unmet need. Drug development has been hampered by a dearth of in vitro and in vivo models that accurately reflect the physiological disease presentation.

**Methods**: We characterized a genetic rat model which is homologous to Meckel Gruber Syndrome III. The homozygous animals (TMEM67−/−) have severe hydrocephalus and survive to post-natal day 18–20. The heterozygous animals (TMEM67±) have a mild hydrocephalus present before weaning but not manifested as overt disease until 1 year of age. Animal studies are complemented by electrophysiological studies of ion transport in a choroid plexus epithelial cell line.

**Results**: In cultured cells an agonist of the transient receptor potential, vanilloid 4 (TRPV4) cation channel (GSK1016790A) stimulates a transepithelial ion flux consistent with cation secretion. The ion flux is undoubtedly coupled to secondary water secretion. Agonist-stimulated ion flux is blocked by pre-treatment with either of two TRPV4 antagonists, RN1734 or HC06747. In the homozygous animals, treatment with RN1734, 4 mg/kg BW i.p. daily from day 7 to day 15 substantially reduces the degree of hydrocephalus measured by MRI quantification of cerebral ventricular volume.

**Conclusions**: TRPV4, a channel that can be activated by inflammatory mediators or changes in pressure or osmotic balance, may be a hub protein in the choroid plexus epithelia allowing the cells to respond to various stimuli. Regulation of this transporter may be an important target for the development of pharmaceutical agents to treat all forms of hydrocephalus.

## O19 Experimental intraventricular hemorrhage directly disrupt cell junctions and ventricular trough ADAM 10 activity

### Leandro Castaneyra-Ruiz^1^, James (Pat) McAllister^1^, Diego M Morales^1^, Albert Isaacs^1^, Sonika M Dahiya^2^, Steven L Brody^3^, Juliane Bubeck-Wardenburg^4^, David D Limbrick Jr^1^

#### ^1^Department of Neurosurgery, Division of Pediatric Neurosurgery, Washington University School of Medicine, St. Louis, United States; ^2^Department of Pathology and Immunology, Washington University School of Medicine, St. Louis, United States; ^3^Department of Medicine, Division of Pulmonary and Critical Care Medicine, Washington University School of Medicine, St. Louis, United States; ^4^Department of Pediatrics, Division of Pediatric Critical Care, Washington University School of Medicine, St. Louis, St. Louis, United States

##### **Correspondence:** Leandro Castaneyra-Ruiz

*Fluids and Barriers of the CNS* 2018, **15(Suppl 2):**O18

**Introduction:** Intraventricular hemorrhage (IVH) remains the most frequent and severe neurological complication of premature birth. Post-hemorrhagic hydrocephalus (PHH) occurs in up to one-half of infants who develop IVH and represents the most common cause of pediatric hydrocephalus. Selective mechanistic triggers downstream of IVH likely mediate the pathogenesis of PHH. We have reported that PHH is associated with disruption of N-cadherin-based adherens junctions and ventricular zone (VZ) damage. We hypothesize that ADAM10-mediated proteolysis of N-cadherin underlies VZ disruption in PHH and that ADAM10 inhibition may abrogate the propagation of PHH following IVH.

**Methods:** In our in vitro IVH model we assayed ADAM10’s role in IVH-mediated VZ disruption by quantifying ADAM10-dependent cleavage of N-cadherin. Western Blot and immunohistochemistry was performed after three different treatments of mouse VZ cell cultures with; (1) Vehicle control (DMSO); (2) Syngeneic blood and (3) GI 254023X (inhibitor of ADAM 10) + syngeneic blood. N-cadherin immunohistochemistry on human IVH/PHH post-mortem brain specimens was performed to co-validate the in vitro experiments.

**Results:** In vitro blood treatment was associated with significant disruption of N-cadherin expression (p < 0.05), reduction in the percentage of multiciliated ependymal cells, and increased astroglial activation (p < 0.01) when compared with controls. ADAM10 inhibition preserved the cytological structure of the blood-treated cells; i.e. N-cadherin expression was similar to controls. Examination of the human IVH/PHH tissue showed similar N-cadherin based VZ perturbations as was observed in vitro.

**Conclusions:** These findings suggest that IVH causes VZ disruption via ADAM10-induced impairment of N-cadherin-based junctions and ADAM10 inhibition mitigates this cytopathological condition.

## O20 Test-bench setup for bioimpedance-based monitoring of ventricular dilation

### Carlos Castelar^1^, Fabian Flürenbrock^1^, Anne Benninghaus^2^, Klaus Radermacher^2^, Steffen Leonhardt^1^

#### ^1^Philips Chair for Medical Information Technology, RWTH Aachen University, Germany; ^2^Chair of Medical Engineering, RWTH Aachen University, Germany

##### **Correspondence:** Carlos Castelar

*Fluids and Barriers of the CNS* 2018, **15(Suppl 2):**O19

**Introduction:** It has been suggested that intracranial dynamics play an important role in the pathogenesis of normal pressure hydrocephalus (NPH). To investigate this further, a real-time monitoring of dynamic ventricular enlargement with a bioimpedance sensor has been proposed. The relative conductivity difference between brain parenchyma and cerebrospinal fluid (CSF) makes it possible. A test-bench is therefore necessary to perform in vitro validation of the bioimpedance prototypes.

**Methods:** The electrical properties of various silicone gels to be implemented as brain phantoms have been tested. Initial impedance measurements have been performed on five cylindrical silicone gel probes with an artificial CSF-filled hollow cavity (saline water, conductivity = 17.9 mS/cm) of discrete volumes [20, 40, 60, 80, 100 mL] representing a single ventricle. Using an Agilent LCR meter E4980, a 250 uA current with a 50 kHz frequency is injected through a pair of ring electrodes integrated onto the surface of a drainage catheter. The electrical impedance is measured between the inner electrodes.

**Results:** The measured impedance decreases linearly with increasing volume (58.8 O for a 20 mL ventricular cavity to 10.3 O for 100 mL). This trend is expected as observed in a finite element model (FEM) study.

**Conclusions:** Initial static measurements of discrete volumes show that the validation of a bioimpedance sensor for ventricular size might be possible on a test-bench. Current work is the extension of the setup to an electronically controlled Hardware-in-the-Loop model which portrays intracranial dynamics, for which a Matlab/Simulink model of pulsatile CSF with cardiovascular coupling is available.

## O21 Shunt to recovery: a multidisciplinary approach in severe acquired brain injury rehabilitation

### Giovanna B Castellani^1^, Paolo Mantovani^2^, Giorgio Palandri^2^, Gian Piero Belloni^1^, Pamela Salucci^1^, Valentina Colombo^1^, Francesca C Cava^1^, Ludovica Scuto^1^, Roberto Piperno^3^

#### ^1^Severe Acquired Brain Injury Unit, Montecatone Rehabilitation Institute, Imola, Italy; ^2^Neurosurgery, IRCCS Istituto delle Scienze Neurologiche, Bologna, Italy; ^3^Rehabilitation Medicine and Neurorehabilitation Unit, AUSL Bologna, Italy

##### **Corresponding author:** Giovanna B Castellani

*Fluids and Barriers of the CNS* 2018, **15(Suppl 2):**O20

**Introduction:** Ventricular dilation in severe acquired brain injured patients (SABIPs) remains a great challenge. Despite numerous efforts, it remains difficult to achieve a neuroradiological and clinical diagnosis of hydrocephalus; furthermore, these patients belong to a fragile group, with high comorbidities and risks. It is therefore difficult to estimate the impact of shunt surgery.

**Methods:** We retrospectively reviewed 81 SABIPs admitted to our unit during a 9-year period (2008–2017) and managed in partnership with neurosurgeons of the Institute for Neurological Sciences of Bologna, analyzing the impact of hydrocephalus treatment. All patients underwent shunt surgery. The diagnosis of hydrocephalus was based on serial CT examinations and on clinical characteristics (clinical stagnation with or without hydrocephalus symptoms, cognitive improvement after lumbar puncture). We assessed outcome improvement with LCF (Levels of Cognitive Functioning) and DRS (Disability Rating Scale) scales.

**Results:** Patients showed an overall improvement after shunting. Hemorrhagic patients had shorter LOS (length of stay), early shunt implantation and higher LCF score. 12 patients had complications after shunt implantation; 9 out of 12 had severe complications and had worse outcomes in LCF scores.

Fixed valves had a significant higher relative risk ratio (4.16 p < 0.05) of severe complications.

A significant correlation with LOS was found: the longer the hospitalization, the worse the DRS score.

**Conclusions:** We investigated hydrocephalus SABIPs who presented arrested or slow recovery or neurological and/or cognitive deterioration. A thorough multidisciplinary approach is critical for shunt surgery indication. We could observe good recovery following shunting even in this extremely fragile group of patients.

## O22 MEMS (Micro-Electro-Mechanical-System) based passive hydrogel valve for hydrocephalus treatment

### Junseok Chae^1^, Ruth E. Bristol^2^

#### ^1^Electrical, Computer and Energy Engineering, Arizona State University, Tempe, Arizona, United States; ^2^Department of Neurosurgery, Phoenix Children’s Hospital, Phoenix, Arizona, United States

##### **Correspondence:** Junseok Chae

*Fluids and Barriers of the CNS* 2018, **15(Suppl 2):**O21

**Introduction:** Existing shunts suffer from complications including mechanical malfunctions, obstructions, infections, blockage, breakage, overdrainage, and/or underdrainage. Some of these complications may be attributed to the shunts’ physically large and lengthy course making them susceptible to external forces, siphoning effects, and risks of infection. Additionally, intracranial catheters artificially traverse the brain, and drain the ventricle rather than subarachnoid space.

**Methods:** A MEMS (Micro-Electro-Mechanical-System) based hydrogel check valve offers an alternative treatment approach targeting restoration of near natural CSF dynamics. Reconstruction of this route may potentially offer greater reliability and safety to current failure-prone shunts. The valve, being made of hydrogel, was manufactured via MEMS technology, which aims to regulate the CSF flow between the sub-arachnoid space and the superior sagittal sinus, in essence substituting for the obstructed arachnoid granulations.

**Results:** The benchtop measurements demonstrate the realization of targeted cracking pressures of 20–200 mmH_2_O and operation at − 800–600 mmH_2_O without observable degradation or reverse flow leakage, < − 10 μL/min. Hydrodynamic measurements and over-time tests under physically relevant conditions further demonstrate the valve’s operationally-reproducible properties.

**Conclusions:** The MEMS-based valve has been shown to operate with targeted hydro-static and dynamic specifications as a stand-alone passive unit for hydrocephalus treatment. Results of this work indicate the valve’s potential application in treating hydrocephalus in a safer and more robust manner than current treatment methods. Future work to ensure its reliability and ability to drain CSF in sentient brains will entail in vivo testing in animal models.

## O23 Surgical outcome of 717 patients with idiopathic normal pressure hydrocephalus treated by lumboperitoneal shunt

### Chia-Cheng Chang^1^, Yasutaka Jimi^2^, Kenji Sanpei^3^

#### ^1^Iyemasa Neurosurgical Clinic, Yokohama, Japan; ^2^Department of Neurosurgery, Noushinkeigeka higashiyokohama Hospital, Yokohama, Japan; ^3^Department of Neurosurgery, Nishiyokohama International Hospital, Yokohama, Japan

##### **Correspondence:** Chia-Cheng Chang

*Fluids and Barriers of the CNS* 2018, **15(Suppl 2):**O22

**Introduction:** The aim of this study was to investigate the surgical outcome of the patients with idiopathic normal pressure hydrocephalus (iNPH) treated by lumboperitoneal (LP) shunt using the Codman programmable valve.

**Methods:** 717 patients were managed between January 2011 and April 2018. The surgery was indicated for patients with the finding of disproportionately enlarged subarachnoid-space hydrocephalus or those positively responded to lumbar tap. The Codman Hakim programmable valve (CHPV) was utilized in 530 patients and the CODMAN CERTAS Plus valve (CCPV) in 187. The valve pressure was set at 130–200 mmH_2_O or setting 7 initially, and readjusted by 10–20 mmH_2_O or one setting level interval. Valve pressure was raised to the maximum of 200 mmH_2_O or Setting 8, when subdural hematoma (SDH) was detected.

**Results:** A favorable response to the surgery within 3 months was detected in 96.5%. Shunt malfunction was observed in 37 (5.2%) patients. Lumbar catheter-related; displacement in 19, disconnection in 5, and obstruction in 2. Peritoneal catheter-related; disconnection in 7 and displacement in one. Obstruction of the valve was found in 2. Complications; surgery-required SDH due to overdrainage was found in 10 (1.4%) patients treated by the CHPV, lumbar pain followed by catheter removal in 6. Shunt infection was found in 3 (0.4%) patients; meningitis in 2, operative wound infection in one.

**Conclusions:** It is safe and useful to treat iNPH by using the LP shunt incorporating with CHPV or CCPV. The use of the CCPV seems to be more helpful to reduce the incidence of surgery-required SDH.

## O24 CSF drainage increases brain parenchymal oxygen tension after subarachnoid haemorrhage

### Claudia L Craven^1^, Ugan Reddy^1^, Lewis T Thorne^1^, Laurence D Watkins^1^, Ahmed K Toma^1^

#### ^1^National Hospital for Neurology and Neurosurgery, Queen Square, London, United Kingdom

##### **Correspondence:** Claudia L Craven

*Fluids and Barriers of the CNS* 2018, **15(Suppl 2):**O23

**Introduction:** CSF diversion with external ventricular (EVD) or lumbar drain (LD) is common following aneurysmal subarachnoid haemorrhage (aSAH). At this single centre, invasive tri-modal monitoring of ICP, temperature and direct brain tissue oxygen tension (PbtO2) is used to guide management for delayed cerebral ischaemia. In this study we observe the immediate effect of CSF drainage on PbtO2.

**Methods:** Inclusion: Patients with aSAH who underwent over 24-h of multi-modal PbtO2, temperature and intracranial pressure (ICP) monitoring via a Raumedic NEUROVENT-PTO^®^probe, during insertion of either EVD or LD. PbtO2 values are presented as mmHg (mean ± SD).

**Results:** Seven patients underwent CSF diversion (2 LD and 4 EVD) inserted, with simultaneous tri-modal monitoring. LD or EVD insertion resulted in a significant decrease in ICP of 11.3 ± 2.75 mmHg over the first 5 min (p = 0.034). A simultaneous mean increase of 7.6 ± 2.94 mmHg in PbtO2 was observed over the same time period (p = 0.002). In all seven patients the global reduction in ICP and increase in PbtO2 was sustained. The greatest increase was observed in the two patients who underwent LD, whom had PbtO_2_ increases of 17 mmHg and 16 mmHg respectively.

**Conclusions:** CSF diversion following acute aSAH can reduce ICP and simultaneously increase PbtO2, potentially addressing both hydrocephalus and delayed cerebral ischemia. Further research into the effect of caudal vs. cranial diversion may demonstrate a superior modality.

## O25 Shunting slit ventricles: a comparison of the Parieto-Occipital vs. Frontal approach

### Claudia L Craven^1^, Linda D’Antona^1^, Simon Thompson^1^, Joana Ramos^1^, Lewis T Thorne^1^, Laurence D Watkins^1^, Ahmed K Tomad^1^

#### ^1^National Hospital for Neurology and Neurosurgery, Queen Square, London, United Kingdom

##### **Correspondence:** Claudia L Craven

*Fluids and Barriers of the CNS* 2018, **15(Suppl 2):**O24

**Introduction:** Slit ventricles can be a challenging target during shunt catheter insertion. Traditionally, the frontal approach has been considered optimial. At this centre, routine use of electromagnetic (EM) stereotatic guidance (Stealth™, Medtronic) has enabled a parieto-occipital burr hole approach to the frontal horns. We compare shunt placement and revisions required for patients with slit ventricles who had shunts inserted via a parieto-occipital (P-O) approach vs. frontal shunt.

**Methods:** Retrospective cohort of patients with slit ventricles and a ventricular shunt inserted using EM guidance between 2012 and 2018. Slit-like ventricles were defined as the widest point of the lateral ventricle < 3 mm.

Outcome measures included placement accuracy and survival using Kaplan–Meier curve. Optimal final catheter tip location was considered to be the frontal horn of the ipsilateral lateral ventricle.

**Results:** 82 patients (77F:5M) aged 34.9 ± 10.8 years (mean ± SD) had ventricular shunts inserted for IIH (n = 63), chiari/syrinx (n = 8), congenital (n = 10), pseudomeningocoele (n = 1). Of the those indentified, 35 had primary P-O shunts and 46 had frontal shunts.

Overall, 94% of cases had the catheter tip sitting in the frontal horn. The P-O approach was just as accurate as the frontal approach. Eight P-O shunts and nine frontal shunts required revision over a 60-month periods. There was no significant different in shunt survival between the two approaches (p = 0.99).

**Conclusions:** EM guided placement has enabled the P-O approach to be as safe and with equivalent survival to frontal approach. The accuracy of shunt placement between the two approaches was equivocal.

## O26 Principles of testing hydrocephalus shunts in vivo using infusion test

### Zofia Czosnyka^1^, Afroditi Lalou^1^, Matthew Garnett^1^, Eva Nabbanja^1^, Romain Manet^2^, Laurent Gergelé^3,4^, Marek Czosnyka^1^

#### ^1^Division of Neurosurgery, University of Cambridge, CB2 1TN, UK; ^2^Department of Neurosurgery B, Neurological Hospital Wertheimer, University of Lyon, France; ^3^Department of intensive care, Ramsay Générale de Santé, Hôpital privé de la Loire, Saint Etienne, France; ^4^Division of Neurosurgery, Department of Clinical Neuroscience, Addenbrooke’s Hospital, University of Cambridge, United Kingdom

##### **Correspondence:** Zofia Czosnyka

*Fluids and Barriers of the CNS* 2018, **15(Suppl 2):**O25

**Objective:** Hydrocephalus shunts may fail after implantation. The quantitative evidence and comparative analysis are needed to justify revision. Our previous study in mixed population of patients suspected of shunt malfunction who underwent infusion test to assess the shunt performance in vivo, showed that in approximately 50% cases shunt malfunction was not confirmed. These patients, if revised surgically, would not benefit from surgery.

**Methods:** Infusion study can be performed via two 25G butterfly needles inserted into shunt prechamber. Free aspiration of CSF is important marker of ventricular inlet patency.

The results of infusion study are analysed and interpreted with consideration of the hydrodynamic properties of the valve in situ (results of Cambridge Shunt Evaluation Program). After the infusion the tilting test can be performed.

**Results:** Around 2000 tests were performed in years 1993–2014 in shunted patients (10% paediatric patients). Following principles were formulated:

1. Presence of detectable pressure pulse waveform is essential marker for patency of ventricular drain.
2. Pressure increase during infusion above hunt’s critical pressure is a reliable indicator of shunt underdrainage or blockage.
3. Abnormally elevated abdominal pressure can result in underdrainage despite a shunt system being patent.
4. If during the ‘tilting test’, pressure decreases below − 10 mmHg overdrainage is possible.
5. Test is safe, with sterile mode of preparation of skin and tubing/needles, rate of infection is less than 1%

**Conclusion**: Infusion test allows to assess the shunt system in vivo (blockage: proximal or distal, overdrainage) avoiding unnecessary shunt revision.

## O27 Components of ICP observed in CSF disorders

### Zofia Czosnyka^1^, Afroditi Lalou^1^, Eva Nabbanja^1^, Matthew Garnett^1^, Romain Manet^2^, Laurent Gergelé^3,4^, Marek Czosnyka^1^

#### ^1^Division of Neurosurgery, University of Cambridge, UK; ^2^Department of Neurosurgery B, Neurological Hospital Wertheimer, University of Lyon, France; ^3^Department of intensive care, Ramsay Générale de Santé, Hôpital privé de la Loire, Saint Etienne, France; ^4^Division of Neurosurgery, Department of Clinical Neuroscience, Addenbrooke’s Hospital, University of Cambridge, United Kingdom

##### **Correspondence:** Zofia Czosnyka

*Fluids and Barriers of the CNS* 2018, **15(Suppl 2):**O26

**Objective:** Different mechanisms can contribute to abnormal ICP in CSF disorders. Generally, they can be classified as those related to abnormal CSF circulation, abnormal arterial blood inflow and venous blood outflow. They are dominant in various types of pathologies, and their recognition may influence the way a patient should be managed.

**Methods:** This is an observational study, summarizing our experience of 5000 infusion tests and 400 overnight ICP monitoring, over the period 1992–2017, in patients suffering from various CSF disorders.

**Results:** Apart from pulse and respiratory components, arterial blood inflow fluctuates with a frequency 0.005 to 0.05 Hz, transferred to ICP in a form of ‘B waves. These waves increase mean ICP during REM phase of sleep. If magnitude of ICP increases above 25 mmHg, they are clearly pathological. In NPH (less than 2%), they signify state of dynamic decompensation, requiring shunt placement.

Components related to CSF circulation are less frequently observed in NPH, but dominant in acute hydrocephalus. They can be identified as elevated baseline pressure, and in infusion test as elevated resistance to CSF outflow (> 15 mmHg/(mL/min)).

IIH is related to impairment of venous blood outflow: ICP is elevated (> 20 mmHg) mainly because of elevation of sagittal sinus pressure (SSP). This may have a dynamic nature, due to positive coupling between ICP and SSP. Transverse sinus stenting and/or VP shunting is generally required.

**Conclusion:** ICP may be elevated due to different mechanisms. Recognition of them may be helpful in optimizing management of our patients.

## O28 Correlation of lumbar puncture opening pressure with 24 h intraparenchymal ICP monitoring: the effects of position on ICP

### Linda D’Antona^1^, Claudia L Craven^1^, Simon D Thompson^1^, Hasan Asif^1^, Jonathan Funnell^1^, Saniya Mediratta^1^, Joana Ramos^1^, Suraj Sennik^1^, Lewis Thorne^1^, Ahmed K Toma^1^, Laurence D Watkins^1^

#### ^1^Victor Horsley Department of Neurosurgery, National Hospital for Neurology and Neurosurgery, London, United Kingdom

##### **Correspondence:** Linda D’Antona

*Fluids and Barriers of the CNS* 2018, **15(Suppl 2):**O27

**Introduction:** Lumbar puncture opening pressure in lateral decubitus has been considered the gold standard method of intracranial pressure (ICP) measurement for many years. The use of continuous intraparenchymal ICP monitoring is more recent and there is no consensus regarding what can be considered normal ICP with this method of measurement.

A conversion factor between lumbar puncture opening pressure and 24 h ICP monitoring could provide a better insight on the interpretation of ICP.

This study investigates the differences between 24 h ICP and ICP in lumbar puncture position.

**Methods:** Single centre prospective study.

Patients investigated with 24 h ICP monitoring who underwent a short exercise battery during the monitoring period were included.

The exercise battery was standardised; patients were asked to stay in a supine, sitting, standing and lumbar puncture position for 2 min each. Mean ICP and pulse amplitude were calculated for each position.

**Results:** Fifty-four patients (42F:12M, mean age 38 ± 12 years) were included.

The mean 24 h ICP was 4.9 mmHg (± 6.9SD) and the mean ICP in lumbar puncture position was 14.1 mmHg (± 8.9SD).

The average increase in lumbar puncture position was 9.1 mmHg (± 5.9SD).

Patients with normal lumbar puncture position ICP (< 12 mmHg) had an average 24 h ICP of 1.43 mmHg (± 2.81 SD).

**Conclusions:** Our results suggest that ICP measured in lumbar puncture position is on average 9.1 mmHg higher than 24 h ICP results. Larger studies will be needed to confirm these findings.

## O29 Telemetric ICP monitoring after neuroendoscopic treatment of hydrocephalus: a valuable tool to recognize treatment failure

### Linda D’Antona^1^, Claudia L Craven^1^, Simon D Thompson^1^, Suraj Sennik^1^, Hasan Asif^1^, Saniya Mediratta^1^, Jonathan Funnell^1^, Joana Ramos^1^, Lewis Thorne^1^, Laurence D Watkins^1^, Ahmed K Toma^1^

#### ^1^Victor Horsley Department of Neurosurgery, National Hospital for Neurology and Neurosurgery, London, United Kingdom

##### **Correspondence:** Linda D’Antona

*Fluids and Barriers of the CNS* 2018, **15(Suppl 2):**O28

**Introduction:** The clinical and imaging improvement of hydrocephalic patients treated with neuroendoscopy can often be delayed. In these cases, the suspicion of treatment failure could mislead the clinician and trigger the unnecessary conversion to a cerebrospinal fluid (CSF) drainage shunt system.

This study investigates the utility of post-neuroendoscopy intracranial pressure (ICP) monitoring employing a new generation non-invasive ICP measuring reservoir.

**Methods:** This is a single centre prospective observational study conducted since August 2017.

Consecutive patients treated for hydrocephalus with neuroendoscopy and the implantation of a telemetric ICP measuring reservoir were included.

Information regarding clinical outcomes, imaging outcomes and post-operative ICP were collected.

**Results:** Five patients (3F, 1M) with a mean age of 28 years (± 8 SD) were included.

ICP measurements in the post-operative period allowed to confirm the success of neuroendoscopic treatment in two patients who presented clear clinical and imaging improvement.

Two patients had persisting symptoms and no imaging improvement on brain MRI and CSF flow studies. In these cases, post-operative ICP measurements were persistently raised and confirmed neuroendoscopic treatment failure, therefore patients underwent the conversion to CSF drainage shunts.

Finally, normal post-operative ICP in a patient with new post-operative speech disturbances permitted to exclude ETV failure and avoided further unnecessary treatment.

**Conclusions:** Non-invasive ICP monitoring after neuroendoscopic procedures can be performed through the use of a new generation telemetric ICP measuring device and represents a valuable tool for the recognition of patients requiring conversion to a CSF drainage shunt.

## O30 Spontaneous retinal venous pulsation: towards non-invasive assessment of ICP

### Linda D’Antona^1^, James McHugh^2^, Claudia L Craven^1^, Hasan Asif^1^, Simon D Thompson^1^, Suraj Sennik^1^, Joana Ramos^1^, Lewis Thorne^1^, Laurence D Watkins^1^, Fion Bremner^2^, Ahmed K Toma^1^

#### ^1^Victor Horsley Department of Neurosurgery, National Hospital for Neurology and Neurosurgery, London, United Kingdom; ^2^Ophthalmology Department, National Hospital for Neurology and Neurosurgery, London, United Kingdom

##### **Correspondence:** Linda D’Antona

*Fluids and Barriers of the CNS* 2018, **15(Suppl 2):**O29

**Introduction:** The absence of spontaneous retinal venous pulsation (SVP) is considered a sign of raised intracranial pressure (ICP) and could represent a useful tool for the non-invasive assessment of ICP. However, this correlation has not been confirmed before and this sign is not currently used in the clinical practice.

This study investigates the correlation between absence of SVP and ICP with a novel approach that employs simultaneous infrared retinal video recordings and intraparenchymal ICP monitoring.

**Methods:** This is a single centre prospective observational study.

Patients who received an ophthalmology assessment simultaneously with continuous ICP monitoring were selected.

Infrared retinal videos were recorded through the use of an Optical Coherence Tomography (OCT) machine. The videos were assessed by two ophthalmologists blinded to the ICP results. The ICP monitoring data were analysed following a standardised local protocol.

A linear regression analysis was conducted to investigate the correlation between absence of SVP and simultaneous mean ICP. Sensitivities and specificities of the absence of SVP at different ICP cut-offs were calculated.

**Results:** 105 patients (79F:26M, age 39 ± 13 years) were included.

Seventeen patients had no SVP and a mean ICP of 12 mmHg (± 12 SD). The remaining 88 patients had SVP and a mean ICP of 1 mmHg (± 5 SD). A linear regression showed a statistically significant correlation between ICP and absence of SVP (P = 0.01, 95% confidence interval − 11.2 to − 1.6).

**Conclusions:** Our results suggest that the absence of SVP is an indirect sign of raised ICP.

## O31 The utility of continuous ICP monitoring in patients with suspected CSF leaks

### Linda D’Antona^1^, Lewis Thorne^1^, Laurence D Watkins^1^, Ahmed K Toma^1^, Manjit S Matharu^2^

#### ^1^Victor Horsley Department of Neurosurgery, National Hospital for Neurology and Neurosurgery, London, United Kingdom; ^2^Department of Neurology, National Hospital for Neurology and Neurosurgery, London, United Kingdom

##### **Correspondence:** Linda D’Antona

*Fluids and Barriers of the CNS* 2018, **15(Suppl 2):**O30

**Introduction:** Spontaneous and iatrogenic cerebrospinal fluid (CSF) leaks present with debilitating orthostatic headaches. Despite the availability of multiple imaging techniques, a CSF leak can be difficult to identify in a significant proportion of patients. Moreover, recent studies have highlighted how lumbar puncture opening pressure can often be normal (rather than low) and this contributes to make the diagnosis of these conditions more complicated. This study aims to describe the utility of continuous intracranial pressure (ICP) monitoring in the management of patients with suspected CSF leaks.

**Methods:** This is a single centre retrospective study. Patients affected by orthostatic headache who underwent ICP monitoring for a suspected spontaneous or iatrogenic CSF leak were included.

**Results:** Fifty-seven patients with orthostatic headache (36F:21M, mean age 45 ± 14 years) underwent continuous 24 h ICP monitoring for a suspected CSF leak. The ICP results confirmed the presence of a low-pressure state in 20 of the selected patients (35%) despite the absence of an identifiable CSF leak on imaging. Seven patients (12%) demonstrated raised ICP in keeping with a diagnosis of idiopathic intracranial hypertension.

**Conclusions:** Continuous ICPM can be a useful additional method of investigation for patients affected by long-standing orthostatic headache and it should be considered when a suspected CFS pressure syndrome cannot be confirmed or excluded with more conventional investigations.

## O32 Hypoxia contributes to the origin of age-related hydrocephalus by a process that depends on AQP4

### José Luis Trillo Contreras^1,2^, Reposo Ramírez-Lorca^1,2^, Laura Hiraldo González^1,2^, Ismael Sánchez-Gomar^1^, Ana Galán-Cobo^1^, Nela Suárez-Luna^1,2^, Eva Sánchez de Rojas-de Pedro^1^, Magdalena Olivares Blanco^1,4^, María Oliver^1,4^, Emilio Franco Macías^1,4^, María Bernal^145^, Juan José Toledo Aral^1,2,3^, Javier Villadiego^1,2,3^, Miriam Echevarría^1,2^

#### ^1^Instituto de Biomedicina de Sevilla-IBiS, HUVR/CSIC/Universidad de Sevilla, Sevilla, Spain; ^2^Departamento de Fisiología Médica y Biofísica, Universidad de Sevilla, Sevilla, Spain; ^3^Centro de Investigación Biomédica en Red sobre Enfermedades Neurodegenerativas (CIBERNED), Spain; ^4^Unidad de Gestión Clínica de Neurociencias, Servicio de Neurocirugía del Hospital Universitario Virgen del Rocío, Sevilla, Spain

##### **Correspondence:** Miriam Echevarría

*Fluids and Barriers of the CNS* 2018, **15(Suppl 2):**O31

**Introduction:** AQP4 present in ependymal cells, glia limiting membranes and pericapillary astrocytes foot processes; and AQP1 expressed in choroid plexus epithelial cells are believed to play an important role in the cerebrospinal fluid (CSF) production and may be involved in the pathophysiology of age-dependent hydrocephalus. The finding that brain AQPs expression is regulated by low oxygen tension and ageing led us to analyze how hypoxia and elevated levels of cerebral AQPs may increase the CSF production that could be associated with the hydrocephalus onset.

**Methods:** Here we have explored in young and aged mice exposed to hypoxia whether expression levels of AQP4 and AQP1 were affected. Choroid plexus, striatum, cortex and ependymal tissue were analyzed separately both for mRNA and protein levels. Moreover, parameters such as intraventricular pressure (IVP), outflow rate of CSF and ventricular compliance measured by intra ventricular recordings in live animals, as well as total ventricular volume, measured by resonance magnetic images (RMI), were estimated. Experiments were done using WT, AQP4^−/−^ and AQP1^−/−^ mice.

**Results:** Our data demonstrate that hypoxia participates in the origin of hydrocephalus by a process that depends on AQP4 as a main route for CSF movement. Significant increases in AQP4 expression that occur along animal’s aging contribute to produce a considerably worse hydrocephalus situation related with hypoxic events, with impairment of the cognitive function.

**Conclusions:** We propose that physiological events and/or pathological situations coursing with brain hypoxia/ischemia, along live span would contribute to development of chronic adult hydrocephalus.

## O33 Outcomes and complications of different surgical treatments for idiopathic normal pressure hydrocephalus: a systematic review and meta-analysis

### Enrico Giordan^1^, Giorgio Palandri^2^, Giuseppe Lanzino^1^, Mohammad Hassan Murad^3^, Benjamin D. Elder^1^

#### ^1^Department of Neurosurgery, Mayo Clinic, Rochester, Minnesota, United States; ^2^Department of Neurological and Visual Sciences, Universita di Verona, Verona, Italy; ^3^Evidence-based Practice Center, Mayo Clinic, Rochester, Minnesota, United States

##### **Correspondence:** Guiseppe Lanzino

*Fluids and Barriers of the CNS* 2018, **15(Suppl 2):**O32

**Purpose:** Different CSF diversion procedures (ventriculoperitoneal, ventriculoatrial, and lumboperitoneal shunting) have been utilized for treatment of idiopathic normal pressure hydrocephalus. More recently, endoscopic third ventriculostomy (ETV) has been suggested as a reasonable alternative in some studies. The purpose of this study was to perform a systematic review and meta-analysis to assess overall rates of favorable outcomes and adverse events for each of these treatments. An additional objective was to determine the outcomes and complication rates in relation to the type of valve utilized (fixed versus programmable).

**Methods:** Multiple databases (PubMed, Epub Ahead of Print, Ovid Medline In-Process & Other Non-Indexed Citations, Ovid MEDLINE, Ovid EMBASE, Ovid Cochrane Central Register of Controlled Trials, Ovid Cochrane Database of Systematic Reviews, and Scopus EMBASE) were searched for studies involving patients with idiopathic ventriculomegaly, no secondary cause of hydrocephalus, opening pressure < 25 mmHg on high volume tap or drainage trial, and age > 60 years. Outcomes included the rate of patients who showed improvement in gait, cognition, and bladder function. Adverse events considered in the analysis included postoperative ischemic/hemorrhagic complications, subdural fluid collections, seizures, need for revision surgery, and infection.

**Results:** A total of 33 studies, encompassing 2461 patients, were identified. Approximately > 75% of patients improved after shunting, without significant differences among the different techniques utilized. Overall, gait improvement was observed in 75% of patients, cognitive function improvement in more than 60%, and incontinence improvement in 55%. Adjustable valves were associated with a reduction in revisions (12% vs 32%) and subdural collections (9% vs 22%) when compared to fixed valves.

**Conclusions:** Outcomes did not differ significantly among different CSF diversion techniques and overall improvement was reported in more than 75% of patients. The use of programmable valves decreased the incidence of revision surgery and of subdural collections after surgery, potentially justifying the higher initial cost associated with these valves.

## O34 Diffusion tensor imaging in idiopathic normal pressure hydrocephalus

### Andreas Eleftheriou^1^, Ida Blystad^2^, Anders Tisell^3,4^, Johan Wallmark^5^, Fredrik Lundin^1^

#### ^1^Department of Neurology and Department of Clinical and Experimental Medicine, Linköping University, Linköping, Sweden; ^2^Department of Radiology, and Department of Medical and Health Sciences, Linköping University, Linköping, Sweden; ^3^Department of Radiation Physics, and Department of Medical and Health Sciences, Linköping University, Linköping, Sweden; ^4^Center for Medical Image Science and Visualisation (CMIV), Linköping University, Linköping, Sweden; ^5^Department of Internal Medicine, and Department of Medical and Health Sciences, Linköping University, Norrköping, Sweden

##### **Correspondence:** Andreas Eleftheriou

*Fluids and Barriers of the CNS* 2018, **15(Suppl 2):**O33

**Introduction:** A promising imaging tool, both for differentiating iNPH from other neurodegenerative diseases and evaluating reversibility, is diffusion tensor imaging (DTI). DTI is an MRI-based, noninvasive technique quantifying white mater lesions. We used DTI in iNPH to investigate possible reversibility as well as apparent diffusion coefficient (ADC) differences between patients and healthy controls (HI).

**Methods:** Thirteen iNPH-patients, (6 m/7f), mean age (MA) 77 (49–81) years were included. The patients underwent a pre- and post-operative clinical work-up: 10 m walk time (w10mt) steps (w10ms), TUG-time (TUGt) and steps (TUGs); for cognitive function MMSE. 9 HI, (4m/5f), MA 77 (70–90) years were included. DTI was performed before and 3 months after surgery, HI underwent DTI once. ADC differences analyzed by manually placing regions-of-interest (ROIs) along corpus callosum (CC) (genu and splenium), internal capsule right (Cidx) and left (Cisin), centrum semiovale right (Csdx) and left (Cssin), two in frontal white matter right (FWMdx) and two in left (FWMsin), thalamus right (THdx) and left (THsin).

**Results:** In patients motor function (w10mt, TUGt) was significantly improved (p = 0.03 respectively p = 0.01) after surgery. Significantly ADC-differences values were found between patients and HI in FWMdx (p = 0.02) and almost significant (p = 0.053) pre- vs postoperatively. There was also a significant change in the CC-splenium area (p = 0.01) between HI and iNPH (postop).

**Conclusion:** Significant ADC-changes in the frontal periventricular area between HI and patients indicate possible disease-related pathology. The almost significant changes pre-postop suggest reversibility of this pathology. This could be of interest to study further with the aim to improving diagnostics in iNPH.

## O35 A new tool for gait analysis in INPH patients

### Michael J. Fritsch^1^, Wasim Arouk^1^

#### ^1^Department of Neurosurgery, DBKNB, Neubrandenburg, Germany

##### **Correspondence:** Michael J. Fritsch

*Fluids and Barriers of the CNS* 2018, **15(Suppl 2):**O34

**Objective:** Tap test with gait analysis is considered to be the Gold standard for testing patients with suspected iNPH. Parallel to our standard evaluation algorithm (walking 10 m, counting the number of steps and taking the time for distance) we evaluated a new tool, the “RehaGait”. This is a professional mobile gait analysis tool, mainly utilized in rehabilitation facilities. Different parameters, such as the duration, frequency, stride length and number of steps, as well as gait, cycle variability, speed, the angle of the foot, ground clearance and more can be measured.

**Materials and methods:** From April 1st 2017 to March 31st 2018 a number of 31 patients (20 male, 11 female) underwent evaluation using the RehaGait. Age range was between 56 and 87 years (mean 75). Patients had a follow up between 2 and 14 months (mean 6 mo).

**Results:** The tool was easy to use. It has several advantages and benefits. A reference data base allows assessment of the patients overall gait pattern [for example ataxtic (cerebellar) or parkinson gait]. Data can be stored on the device and allow to compare interindividuell (for example pre and post Tap test, after shunt, at follow up 1 year later). Based on the Tap test and the gait analysis using the ReahGait we opted for shunt surgery in 21 out of 31 patients.

**Discussion and Conclusion:** “RehaGait” is adding new informations to the testing of iNPH patients and the reliable opportunity to compare patients individually on long term follow up. It will allow new insights into prediction of shunt responsiveness in the future.

## O36 Adjustable valve versus adjustable gravitational unit in the therapy of idiopathic normal pressure hydrocephalus - a prospective randomized study

### Michael J. Fritsch^1^, Susann Pietz^1^

#### ^1^Department of Neurosurgery, DBKNB, Neubrandenburg, Germany

##### **Correspondence:** Michael J. Fritsch

*Fluids and Barriers of the CNS* 2018, **15(Suppl 2):**O35

**Objective:** It is a controversial issue whether it is of greater importance to implant vp-shunts with adjustable valves or adjustable gravitational units. The intention of this trial is to compare two groups of iNPH-patients in regard to valve adjustments, complications and clinical outcome.

**Methods:** Patients with iNPH were randomized to receive a VP-Shunt either with an adjustable valve (pressure setting 05/20) or an adjustable gravitational unit (pressure setting 05/20). After surgery the patients underwent follow-up examinations after 3, 6, 9 and 12 months and the outcome was assessed through specific iNPH grading scales like Kiefer Score, NPH Recovery Rate and Black Grading Scale.

**Results:** From March 2014 to May 2017 40 patients were included in the study. In the adjustable gravitational unit cohort 35% of the patients were evaluated with excellent und 20% with good results. Currently only one patient of the adjustable valve group aimed excellent results (5%) but 35% achieved a good clinical outcome measured by Black Grading Scale. The rate of revision operations because of catheter dysfunction or subdural hematoma was higher in the group with adjustable valves with 15% in contrast with 5% in the adjustable gravitational unit group. The frequency of overdrainage complications was also higher among the patients with adjustable valves with 10% vs. 5%. The necessity of valve adjustments due to underdrainage was fairly equal with 40% in both groups.

**Conclusion:** The results of this study reveal the supremacy of adjustable gravitational units over adjustable valves for patients with iNPH.

## O37 Treatment implications of Parkinson’s disease in normal pressure hydrocephalus

### Jonathan P Funnell^1,2^, Claudia L Craven^1^, Linda D’Antona^1^, Lewis Thorne^1^, Laurence D Watkins^1^, Ahmed K Toma^1^

#### ^1^Victor Horsley Department of Neurosurgery, National Hospital for Neurology and Neurosurgery, London, United Kingdom; ^2^UCL Medical School, University College London, London, United Kingdom

##### **Correspondence:** Jonathan P Funnell

*Fluids and Barriers of the CNS* 2018, **15(Suppl 2):**O36

**Introduction:** Patients with idiopathic Parkinson’s Disease (PD) presenting with worsening gait and ventriculomegaly could have underlying normal pressure hydrocephalus (NPH). We aim to identify features of concurrent PD and NPH, assess investigation methods and benefits of intervention.

**Methods:** Retrospective single-centre cohort of patients diagnosed with PD and NPH (2004–2018). Medical records were studied for demographic information, symptom progression, and response to ventriculoperitoneal (VP) shunting. Chi square test was used to compare frequency of post-operative symptoms against a local database of NPH patients.

**Results:** 24 patients (20M: 4F) with concurrent PD and NPH, mean age 74.5 ± 6.49 years (mean ± SD). 22 patients were diagnosed with PD prior to NPH diagnosis. Gait disturbances were present in all patients, cognitive impairment in 21/24, and urinary incontinence in 18/24.

19 patients underwent VP shunt insertion; five patients were not suitable having failed a trial of lumbar drainage. Patients with PD and NPH improved in walking test outcomes and in urinary continence similarly to other NPH patients in response to VP shunting. Cognitive impairment did not respond well to VP shunting in patients with concurrent PD, significantly less than NPH patients without PD (p < 0.01).

**Conclusions:** Diagnosis of NPH in patients with PD is a complex clinical problem due to frequent overlap of symptoms. Benefits may be gained if this subset of patients do reach neurosurgical services and receive intervention, however expectations may need to be managed regarding cognitive impairment.

## O38 Outcomes of surgical intervention in IIH without papilloedema

### Jonathan P Funnell^1,2^, Linda D’Antona^1^, Claudia L Craven^1^, Simon D Thompson^1^, Aswin Chari^1^, Lewis Thorne^1^, Laurence D Watkins^1^, Ahmed K Toma^1^

#### ^1^Victor Horsley Department of Neurosurgery, National Hospital for Neurology and Neurosurgery, London, United Kingdom; ^2^UCL Medical School, University College London, United Kingdom

##### **Correspondence:** Jonathan P Funnell

*Fluids and Barriers of the CNS* 2018, **15(Suppl 2):**O37

**Introduction:** Surgical intervention for IIH without papilloedema (IIHWOP) is controversial and its effect on symptoms is poorly understood. This study aims to evaluate which interventions are used in patients with IIHWOP and the effect this has on their symptoms.

**Methods:** Retrospective study of patients undergoing 24-h ICP monitoring for the investigation of IIH (September 2006–March 2018). Patients were selected by diagnosis from a local database of patients undergoing 24-h ICP monitoring with no prior neurosurgical intervention. Clinical records were reviewed for records of symptoms, investigations and interventions.

**Results:** 19 patients diagnosed with IIHWOP (18F: 1M) with a mean age 32.1 ± 7.8 years (mean ± SD). All patients complained of chronic daily headache; 9/19 reported visual disturbances.

15 patients were treated surgically for IIH; 6 with venous sinus stenting, 8 by ventriculoperitoneal (VP) shunt, and 1 by lumboperitoneal shunt.

Patients undergoing venous stenting responded well to treatment with 6/6 reporting an improvement in headache symptoms; in severity and/or duration affected. 4/8 of patients undergoing VP shunting noticed an improvement in headaches, as did the patient who underwent an LP shunt. These differences were not statistically significant. VP shunting was associated with higher rates of further intervention than venous stenting, during mean follow up of 29.6 ± 19.4 months (mean ± SD).

**Conclusions:** Patients with IIH in the absence of papilloedema may benefit from surgical interventions and report an improvement in their symptoms. Venous stenting in particular was associated with improvements in symptoms and few further interventions needed.

## O39 External hydrocephalus after traumatic brain injury: retrospective study of 143 patients

### Laurent Gergelé^1,2^, Romain Manet^3^, Angelos Kolias^2^, Zofia Czosnyka^2^, Afroditi Lalou^2^, Peter J Hutchinson^2^, Marek Czosnyka^2^

#### ^1^Department of intensive care, Ramsay Générale de Santé, Hôpital privé de la Loire, Saint Etienne, France; ^2^Division of Neurosurgery, Department of Clinical Neuroscience, Addenbrooke’s Hospital, University of Cambridge, United Kingdom; ^3^Department of Neurosurgery B, Neurological Hospital Wertheimer, University of Lyon, France

##### **Correspondence:** Laurent Gergelé

*Fluids and Barriers of the CNS* 2018, **15(Suppl 2):**O38

**Introduction:** External hydrocephalus (EH) refers to a CSF flow impairment with an enlargement of the subarachnoid spaces concomitant to raised ICP and have not to be confused with a subdural hygroma. We studied the frequency and the consequences of the EH in an adult’s TBI population.

**Materials and methods:** Retrospective analysis of 143 successive TBI patients recorded between 02/2014 and 01/2017 in Cambridge. The inclusion criteria were TBI admitted in ICU with ICP monitoring and at least three CT-scans in the first 21 days. The subarachnoid spaces (SAS) were quantified individually in each CT-scan. A double check of the EH CT-scan was confirmed by independent investigator. The ICP data were also analyzed with ICM + software. The short-time and 6-month evolution were scrutinized. The EH group was compared with the non-EH group.

**Results:** 41/143 patients underwent craniectomy and withdrawal from the analysis. 31/102 patients (30.4%) developed EH. There was no difference in the initial Glasgow Coma Score between the both groups. The principal risk factor to develop EH was subarachnoid hemorrhage. EH group had longer period of mechanic ventilation, more tracheostomies, and longer ICU stay. EH patients had increased pulsatility of ICP. The long-term outcome was also worse in EH group: more secondary hydrocephalus with more shunt necessity (OR = 7.1) and lower mean GOS-E in the survivors: 4.6 vs 5.9.

**Conclusions:** EH is frequent in our study, with significant clinical consequences. The diagnosis is complex. It could be improved by an automated CT-scan temporal analysis to differentiate EH from a subdural hygroma.

## O40 Quality indicators in a normal pressure hydrocephalus excellence center

### Kemel A. Ghotme^1^, Fernando Hakim^1^, Juan F. Ramon^1^, Juan A. Mejia^1^, Diego Gomez^1^, Maria F. Cardenas^1^, Maria T. Dominguez^1^, Sandra Soler^1^, Marcela Daza^1^, Diana Castelblanco^1^, Martha Mora^1^

#### ^1^Department of Neurosurgery, Fundacion Santafe de Bogota, Colombia

##### **Correspondence:** Kemel A. Ghotme

*Fluids and Barriers of the CNS* 2018, **15(Suppl 2):**O39

**Introduction:** Quality indicators are needed to measure performance in the delivery of health care to patients and respond to the need for multidimensional, accessible, interdisciplinary and humane care. There is scarce information regarding design and implementation of quality indicators in excellence centers providing care to patients with normal pressure hydrocephalus.

**Methods:** The design and implementation process of quality indicators at the Normal Pressure Hydrocephalus Excellence Center at Fundacion Santafe de Bogota in Colombia is presented, including the identification of potential indicators, their definition, referents, baselines, methodology and scope. The integral process was validated by an external multinational evaluator (Joint Commission International).

**Results:** We show the full process, list and categories of indicators, as well as the results for the first 2 years of the application of structure, process and results indicators of the Normal Pressure Hydrocephalus Excellence Center at Fundacion Santafe de Bogota, as well as an analysis of the process, interdisciplinary approach, and main limitations of the use of quality indicators to properly assess and improve delivery of health care to NPH patients.

**Conclusion:** Quality indicators allow diagnosis, continuous assessment and improvement of the services provided in a Normal Pressure Hydrocephalus Excellence Center, as confirmed by the results and the external verification. They are useful to gage performance in health care delivery to NPH patients and promote a culture of quality and continuous improvement.

## O41 Patient experiences of awake ICP monitoring

### Aimee Goel^1^, Hasan Asif^2^, Claudia L Craven^2^, Simon Thompson^2^, Joana Ramos^2^, Ahmed K Toma^2^, Laurence D Watkins^2^, Lewis Thorne^2^

#### ^1^University College London Medical School, Gower Street, London, United Kingdom; ^2^National Hospital for Neurology and Neurosurgery, Queen Square, London, United Kingdom

##### **Correspondence:** Hasan Asif

*Fluids and Barriers of the CNS* 2018, **15(Suppl 2):**O40

**Introduction:** Elective intracranial pressure monitoring is useful for the purpose of diagnosing and treating disorders of CSF dynamics and hydrocephalus. We aim to survey individual patient experiences of ICP monitoring bolt insertion and removal.

**Methods:** Prospective consecutive series of patients with elective diagnostic ICP bolt operations in a single centre between June and October 2017. Review of patient completed questionnaires with white space, yes–no and 5-point Likert scale questions.

**Results:** Fifty-two patients (22M:30F, mean age 42.9 ± 16.7), 36 fitted with Spiegelberg and 16 with Raumedics bolts. Duration of procedure was explained in 84.6% and procedural steps explained in 96.2%. Overall insertion experience was 5/5 compared to 3/5 for removal (p = 0.0058). No difference in insertion experience for either bolt, median 5/5 (p = 0.6929). No difference in removal experience for either bolt, median 3/5 (p = 0.3211). Thirty-three patients would have preferred local anaesthetic for removal. Best part of insertion was that it was painless, worst part was the drilling. Best part of removal was having it out and worst was pain and slowness.

**Conclusion:** Patients find bolt removal more unpleasant due to more pain and slowness compared to insertion. There was no difference in bolt insertion or removal experiences between either bolt types. Future interventions could include the use of local anaesthetic and further training of junior staff to remove bolts painlessly and effectively.

## O42 Ventriculoatrial shunt as the first option. Our experience in the management of hydrocephalus in adults

### Diego Gomez^1^, Maria Cardenas^1^, Maria Dominguez^1^, Juan Davidson^1^, Lina Gómez^1^, Juan Ramon^1^, Kemel Ghotme^1^, Juan Armando Mejia^1^, Enrique Jimenez^1^, Fernando Hakim^1^

#### ^1^Neurosurgery Section, Department of Surgery, Hospital Universitario Fundación Santa Fe de Bogotá, Bogotá, Colombia

##### **Correspondence:** Diego Gomez

*Fluids and Barriers of the CNS* 2018, **15(Suppl 2):**O41

**Introduction:** Ventricular shunts represent the axis of treatment of disorders of the cerebrospinal fluid (CSF). The literature shows that the preferred technique around the world is the ventricular peritoneal shunt (VP), which is technically more simple, reproducible and represents a permanent and lasting CSF drainage. In our institution ventriculoatrial derivation is the first choice in the treatment of hydrocephalus in adults, results are equivalent to the technique of choice or even better.

**Methods:** A retrospective review of patients with hydrocephalus who were treated with ventriculoatrial shunting at a single institution, from 2007 to 2017, was performed.

**Results:** We included a total of 106 patients who met the inclusion criteria. 56 (58%) women and 50 (42%) men with an average age of 58.3 years. The dysfunction of the system was presented in 9 (8.5%) patients. 7 of these during the first year, all of them requiring reintervention. Infection was identified in 8 (7.6%) of the patients. 57.5% of patients completed at least 1 year of follow-up.

**Conclusions:** Ventriculoatrial shunt is a valid option in the management of hydrocephalus, in our experience is a safe, reproducible and effective technique. We consider that a DVA shunt is more physiological than traditional technic and less shunt malfunction in the follow up.

## O43 The use of a modified canine ventriculitis model to test a novel polymer-based system of coating shunt catheters with an antimicrobial agent

### Noah L. Gorelick^1^, Rajiv R. Iyer^1^, Karen Carroll^2^, Ari Blitz^3^, Sarah Beck^4^, Betty Tyler^1^, Horst von Recum^5^, Mark G. Luciano^1^

#### ^1^Department of Neurosurgery, Johns Hopkins University School of Medicine, Baltimore, Maryland, United Sates; ^2^Department of Infectious Disease, Johns Hopkins University School of Medicine, Baltimore, Maryland, United Sates; ^3^Department of Radiology, Johns Hopkins University School of Medicine, Baltimore, Maryland, United Sates; ^4^Department of Molecular and Comparative Pathobiology, Johns Hopkins University School of Medicine, Baltimore, Maryland, United Sates; ^5^Department of Biomedical Engineering, Case Western Reserve University, Cleveland, Ohio, United Sates

##### **Correspondence:** Mark G. Luciano

*Fluids and Barriers of the CNS* 2018, **15(Suppl 2):**O42

**Introduction:** Ventricular shunt infections pose a threat to patients with cerebrospinal fluid (CSF) disorders who rely on long-term functioning of implanted flow diversion hardware. To better understand mechanisms of shunt infection and prevention we adapted an existing canine model of ventricular shunting to induce ventriculitis. This model was used to evaluate a novel method of treating shunt catheters with a polymer-based antibiotic delivery system.

**Methods:** Bilateral shunt catheters were introduced into the lateral ventricles of three dogs and then immediately inoculated with methicillin-susceptible *Staphylococcus aureus* (MSSA). Two animals were given catheters pretreated with a modified polymer that contained vancomycin-loaded chemical “pockets”. The third animal’s catheters were coated with a similar polymer yet without vancomycin. Subjects were closely monitored and, upon clinical decline, high-resolution MRI images were obtained, catheters explanted, and brain tissue harvested for histological analysis. Recovered catheters underwent microbiological culturing and scanning electron microscopy.

**Results:** All animals underwent successful ventricular catheter implantation bilaterally without immediate neurological sequelae. By day 10 all animals began clinical deterioration with MRI evidence of ventriculitis and cerebral edema. Growth of MSSA following culture of sonicated catheters was limited to only one of the two implanted catheters among each of the experimental subjects, while both catheters in the control animal produced positive growth.

**Conclusion:** This demonstrates a reliable canine ventriculitis model that can be verified clinically and radiologically, and evaluated microbiologically and histologically. This model has utility in the evaluation of future shunt catheter modifications including the polymer-based antibiotic-coated shunts described.

## O44 Frontal horn/whole brain volume ratio is a better biological measure than Evan’s index of ventricle size

### Neill R Graff-Radford^1^, Julia E Crook^1^, Jeffrey L Gunter^1^, Colleen S Thomas^1^, David T Jones^1^, Jonathan Graff-Radford^1^, Bradley F Boeve^1^, David S Knopman^1^, Clifford R Jack Jr. ^1^, Ronald C Petersen^1^

#### ^1^Mayo Clinic, Jacksonville, Florida & Rochester, Minnesota, United States

##### **Correspondence:** Neill R Graff-Radford

*Fluids and Barriers of the CNS* 2018, **15(Suppl 2):**O43

**Introduction:** The Evans Index (frontal horn/inner table of the skull ratio, EI) has been used as a measure of ventricle size but ventricular volume measures may have stronger biological associations with clinical outcomes. In this study we compare how the EI versus frontal horn/total brain volume ratio (FH) relate to cross sectional measures and longitudinal changes in gait and cognition.

**Methods:** We examined 1774 initial MRI scans in persons over 70 entering the longitudinal population-based Mayo Clinic Study of Aging. We measured EI manually and the computer calculated FH. We document high tight sulci, enlarged sylvian fissures and entrapped fluid pockets. These are features of disproportionate enlarged subarachnoid space hydrocephalus (DESH). A program measuring sulcal size confirmed DESH features. Every 15 months participants undergo neuropsychology testing evaluating executive function, language, memory and visuospatial. Combined scores create a global cognitive score. They also complete a timed walk of 25 feet.

**Results:** With covariate adjustments, EI was cross sectionally associated with gait speed (p = 0.011) but not longitudinally (P = 0.88), while FH was associated both cross sectionally (p < 0.001) and longitudinally (p = 0.034). EI showed some evidence of association with cognition cross sectionally (p = 0.09) and longitudinally (p = 0.004) yet FH showed much stronger evidence of association (p < 0.001 both cross sectionally and longitudinally). Persons with large ventricles (top 30%) who had DESH features were estimated to have a faster decline in gait than those without DESH.

**Conclusion:** FH is a better marker than EI of cross-sectional measures and longitudinal changes of both gait speed and global cognition.

## O45 Automated DESH pattern detection: longitudinal stability and robustness to MRI acquisition variability

### Jeffrey L Gunter^1^, Jonathan Graff-Radford^2^, David Jones^2^, David Knopman^2^, Neill Graff-Radford^3^, Clifford Jack, Jr.^1^

#### ^1^Aging and Dementia Imaging Research Lab, Mayo Clinic, Rochester, Minnesota, United States; ^2^Department of Neurology, Mayo Clinic, Rochester, Minnesota, United States; ^3^Department of Neurology, Mayo Clinic, Jacksonville, Florida, United States

##### **Correspondence:** Jeffrey L Gunter

*Fluids and Barriers of the CNS* 2018, **15(Suppl 2):**O44

**Introduction:** Disproportionately Enlarged Subarachnoid Hydrocephalus (DESH) is included in the Japanese NPH management guideline. Automated machine learning DESH imaging pattern detection (~ 95% sensitivity) on high resolution T1-weighted MRI (N. Gunter et al. 10.1016/j.jalz.2017.06.2345) generates a “computational DESH” (CDESH) score. We estimate reproducibility (a necessity for any possible biomarker) in images from the Mayo Clinic Study of Aging (MCSA) and the 57-site Alzheimer’s Disease NeuroImaging Initiative (ADNI).

**Methods:** CSF volumes in 28 selected sulci, lateral ventricle frontal horn volume and the total intracranial volumes are used to calculate the CDESH score using a support vector machine. The SVM decision margin is, by definition, 2 units wide, providing a comparison scale. In MCSA, the scores within subject at multiple time points provide estimated variability. Scores from ADNI back-to-back identically acquired (BTB-I) scans were evaluated (best-case variability). ADNI back-to-back 2×-accelerated and un-accelerated images (BTB-A) were also assessed.

**Results:** 395 MCSA subjects had more than three good-quality images (single 3T scanner manufacturer); ignoring possible biological change we estimate a standard deviation within each subject’s scores: median standard deviation 0.44 units compared with a standard deviation of all scores from 4180 images from 1597 subjects of 2.28 units and a 2-unit decision margin. 4538 ADNI-BTB-I pairs were highly correlated (slope = 0.99, r^2^ = 0.80, IQR 0.45 units). Linear regression of 4621 ADNI-BTB-A pairs: slope = 0.97, offset = 0.28, r^2^ = 0.90, IQR 0.78, with un-accelerated scores > accelerated.

**Conclusions:** The measurement precision across identically acquired images approximately one-quarter of the SVM margin width. Scores are sensitive to differences in images due to acquisition acceleration.

## O46 Quantification and differentiation of periventricular white matter injury in post-hemorrhagic hydrocephalus using diffusion basis spectrum imaging (DBSI)

### Albert Isaacs^1^, James (Pat) McAllister^1^ Leandro Castaneyra-Ruiz^1^, Diego Morales^1^, Harri Merisaari^1^, Tsen-Hsuan Lin^1^, Travis Crevecoeur^1^, Joel Brown^1^, Alexis Hartmann^1^, Jennifer Strahle^1^, Sheng-Kwei Song^1^, David Limbrick^1^

#### ^1^Department of Neurology, Washington University School of Medicine, St. Louis, Missouri, United States

##### **Correspondence:** Albert Isaacs

*Fluids and Barriers of the CNS* 2018, **15(Suppl 2):**O45

**Introduction:** Periventricular white matter (PVWM) disruption is a dominant pathology in post-hemorrhagic hydrocephalus (PHH), and accounts for long-term morbidity. Diffusion Tensor Imaging (DTI), which is frequently used to assess PVWM integrity, is unable to differentiate complex cellular pathologies such as edema, inflammation and axonal loss. We used Diffusion Basis Spectrum Imaging (DBSI), which has been validated to be more effective for assessing complex WM pathologies in multiple sclerosis, spinal cord injury and brain tumors to address the PVWM pathology associated with PHH.

**Methods:** PHH was induced in 20-day-old ferrets by intraventricular injection of autologous blood (n = 7). Controls (n = 6) received intraventricular PBS. About 50-days-old, their brains were harvested and fixed in 4% PFA, then scanned ex vivo in a Varian^®^ 4.7T MRI for T2-weighted, multi-echo, spin-echo, and diffusion weighted sequences in 99 directions (TR 3000 ms, TE 60 ms, max-*b*-value = 3000 s/mm^2^). Regions of Interest and voxel intensities for corpus callosum (CC), anterior and (ALIC) posterior (PLIC) limbs of the internal capsule were statistically analyzed using Python v2.7 and R package v3.4.1 scripts. Immunohistochemistry was done to assess PVWM.

**Results:** The PHH group had 68% (*p *< 0.005) proportional increase in hindered fraction: 120% (*p *< 0.005) and 51% (*p *< 0.005) in the CC and ALIC, respectively. CC and ALIC demonstrated proportionally decreased fiber density by 7% (*p *< 0.05) and 10% (*p *< 0.05), respectively. ALIC demonstrated axonal injury with decreased axial diffusivity of 8% (p < 0.05). While similar trends were observed in the PLIC, none were statistically significant. Immunohistochemistry demonstrated significantly higher PVWM in PHH than in controls.

**Discussion:** DBSI demonstrated marked edema, neuroinflammation, axonal injury and axonal loss in the PVWM of the PHH cohort, as was seen on immunohistochemistry. DBSI is a versatile tool for differentiating and quantifying the different components of WM disruption, and can be used as a novel non-invasive biomarker for PVWM integrity in PHH.

## O47 Gait analysis in idiopathic normal pressure hydrocephalus on timed-up-and-go-test using free smartphone application

### Masatsune Ishikawa^1,2^, ShigekiYamada^2,3^, Kazuo Yamamoto^3,^ Yukihio Aoyagi^4^, Rokuwa Villa Ilios^1^

#### ^1^Normal pressure Center, Rakuwa Villa Ilios, Kyoto, Japan; ^2^Normal Pressure Hydrocephalus Center, Rakuwakai Otowa Hospital, Kyoto, Japan; ^3^Department of Neurosurgery, Rakuwakai Otowa Hospital, Kyoto, Japan; ^4^Department of administration, Digital Standard Co., Osaka, Japan

##### **Correspondence:** Masatsune Ishikawa

*Fluids and Barriers of the CNS* 2018, **15(Suppl 2):**O46

I**ntroduction:** Gait disturbance is the most common symptom in idiopathic normal pressure hydrocephalus (NPH). In this study, clinical assessment of quantitative measurement on six components of timed-up-and-go test (TUG) was examined in iNPH using the free iPhone application (Senior Quality) (iTUG).

**Methods:** Thirty-two patients with probable iNPH including 19 shunt responsive patients and 87 age-matched control subjects were enrolled. TUG was done twice. Data were automatically collected during iTUG, and the time for the six components were automatically segmented (Stand, Go, Turn1, Come, Turn2 and Sit). The item cluster analysis (ICLUST) was performed to explore underlying structures of iTUG.

**Result**s: In the age-matched control, some components in the second session were shorter than the first one. There were no statistical differences in the NPH. The ICLUST revealed that pairs of the Go and Come, and the Turn1 and Turn2, were two major clusters in the control. This pattern was deranged in the NPH and a pair of the Turn1 and Come became the first cluster. At the tap test, a pair of the Go and Turn2 became the first cluster. After the shunt surgery, the pattern as in the control was regained.

**Conclusions:** The iTUG enables quantitative assessment of six components on TUG. They showed that derangement of the relationship among the components of TUG in iNPH. This derangement was regained after the surgical treatment. This provides further insight into the patho-physiology of gait disturbances in various disorders.

## O48 A panel of CSF biomarkers can distinguish between idiopathic normal pressure hydrocephalus and subcortical ischemic vascular dementia-pathophysiological implications?

### Anne Jeppsson^1^, Maria Bjerke^2^, Per Hellström^1^, Petronella Kettunen^2^, Carsten Wikkelsø^1^, Anders Wallin^2^, Mats Tullberg^1^

#### ^1^Hydrocephalus Research Unit, Institute of Neuroscience and Physiology, The Sahlgrenska Academy, University of Gothenburg, Gothenburg, Sweden; ^2^Clinical Neurochemistry Laboratory, Department of Psychiatry and Neurochemistry, Institute of Neuroscience and Physiology, the Sahlgrenska Academy, University of Gothenburg, Mölndal, Sweden; ^3^Department of Psychiatry and neurochemistry, Institute of Neuroscience and Physiology, The Sahlgrenska Academy, Gothenburg, University of Gothenburg, Sweden

##### **Correspondence:** Anne Jeppsson

*Fluids and Barriers of the CNS* 2018, **15(Suppl 2):**O47

**Introduction:** Patients with subcortical ischemic vascular dementia (SIVaD) exhibit a similar subcortical cognitive impairment as patients with iNPH and can show similar gait, balance and urinary dysfunction. Further, white matter lesions on MRI and enlargement of the brain ventricular system may be present, probably due to brain atrophy. Hence, distinguishing between iNPH and SIVaD, which has a large impact on treatment options, constitutes a major clinical challenge. We aimed at comparatively examine the CSF biomarker expression in a group of iNPH-patients, one group of SIVaD patients and healthy individuals (HI) to enhance the differential diagnostic accuracy.

**Methods:** The study included 52 patients with iNPH (aged 72 ± 7 years, 29 men and 23 women). 17 SIVaD (aged 71 ± 7 years, 5/12) and 28 HI (68 ± 4, 18/9). We analyzed a panel of APP-derived proteins, i.e. Aβ38, Aβ40, Aβ42 and soluble forms sAPPα, sAPPβ, as well as markers of white matter damage NFL, GFAP and MBP.

**Results:** Patients with iNPH exhibited lowered levels of APP-derived proteins in comparison with SVD. Both groups had lowered levels in comparison with HI. NFL, GFAP and MBP was elevated in iNPH and SVD compared to HI. NFL was higher in SIVaD and levels of MBP and GFAP higher in iNPH.

**Conclusions:** The biochemical changes in CSF of patients with iNPH and SIVaD seems to share common features but the levels could be used to differentiate between the conditions. This might implicate common pathological mechanisms in iNPH and SIVaD.

## O49 Bolus lumbar infusion studies in the diagnosis of normal pressure hydrocephalus

### Sarah C. Jernigan^1^, C. Scott Mclanahan^1^, Julie Smith^1^, Lisa Schloeder^1^, Kyley Fisher^1^

#### ^1^Carolina Neurosurgery and Spine Associates, Charlotte, North Carolina, United States

##### **Correspondence:** Kyley Fisher

*Fluids and Barriers of the CNS* 2018, **15(Suppl 2):**O48

This is a retrospective review of 100 consecutive lumbar infusion studies in patients being evaluated for the surgical treatment of symptoms consistent with normal pressure hydrocephalus. Initial evaluations of these patients consisted of a clinical assessment along with an abbreviated scoring system to provide a baseline measurement of symptoms falling within the NPH triad. Patients and their families were then offered further studies including bolus lumbar infusion studies as described by Marmarou, along with a family assessed CSF volume tap test, and other studies as indicated by the results of the LP studies. Follow up studies, including scoring, will be presented and correlated with preop measurements.

While we are aware of the limitations of lumbar infusion studies and family-assessed CSF tap tests, we believe that they can be performed safely and efficiently in an outpatient setting and do provide additional valuable data that is useful in determining which of these patients will be successfully treated with CSF shunt surgery.

## O50 Towards iNPH multimorbidity score system: do we need to exclude patients from surgical treatment?

### Linda D’Antona^1^, Sandra Blamey^2^, Simon D Thompson^1^, Joana Ramos^1^, Claudia L Craven^1^, Suraj Sennik^1^, Hasan Asif^2^, Aida Kafai^2^, Lewis Thorne^1^, Laurence D Watkins^1^, Val Luoma^2^, Ahmed K Toma^1^

#### ^1^Victor Horsley Department of Neurosurgery, National Hospital for Neurology and Neurosurgery, London, United Kingdom; ^2^Anaesthesia and Intensive Care, National Hospital for Neurology and Neurosurgery, London, United Kingdom

##### **Correspondence:** Linda D’Antona

*Fluids and Barriers of the CNS* 2018, **15(Suppl 2):**O49

**Introduction:** It is common practice to exclude severely morbid patients from surgical intervention due to the high risk of complications. Normal Pressure Hydrocephalus (NPH) patients are often affected by multiple comorbidities and the safety of surgical treatment in this group has been debated. This study investigates the safety of surgery in NPH through the use of a post-operative morbidity survey.

**Methods:** Single centre prospective study.

NPH patients referred for lumbar drainage (LD) protocol or ventriculo-peritoneal shunt (VPS) since May 2017 were included. All patients were assessed in a multidisciplinary pre-assessment clinic.

A standardised post-operative morbidity survey was conducted for all patients on day 4, 7 and 10 post-op.

**Results:** 73 consecutive NPH patients (21M, 53F) with a mean age of 74 years (± 7 SD, range 54–90) were referred for surgical treatment. All patients had surgery and none of them was excluded. 33 patients underwent VPS insertion and 40 underwent LD protocol.

Of the 33 patients who underwent VPS insertion: 79% were back to baseline within 3 days, 18% presented a moderate worsening in mobility and 3% a urinary tract infection (UTI).

Of the 40 patients who underwent LD protocol: 90% were back to baseline within 3 days, 7.5% presented a moderate worsening in mobility and 2.5% had a UTI.

All patients who reported a worsening mobility returned to their baseline with physiotherapy before discharge. There was no mortality.

**Conclusions:** Multidisciplinary preadmission assessment and optimisation of NPH patients ensure safety of surgery and avoid denying NPH patients the benefits of surgical treatment.

## O51 Normal pressure hydrocephalus patients: improved selection for shunting – a questionnaire to detect patients with NPH despite negative spinal tap test

### Uwe Kehler

#### Department of Neurosurgery, Asklepios Klinik Hamburg Altona, Neurochirurgie, Hamburg, Germany

##### **Correspondence:** Uwe Kehler

*Fluids and Barriers of the CNS* 2018, **15(Suppl 2):**O50

**Objective:** Patients often experience subjective gait improvement after spinal tap test (STT) although no objective findings are shown in gait tests. Reasons might be the insensibility of tests like TUG, gait velocity or a too early or late investigation after the STT. To overcome both shortcomings a questionnaire was developed to protocol the subjective improvements at different times after the STT.

**Methods:** In the questionnaire the subjective amount of gait improvement after STT had to be protocolled by patients or their relatives hourly on the first day and then once a day for a week. The first questionnaires were evaluated in terms of successful completing by the patients and if the results might help in the indication for shunting.

**Results:** Most but by far not all patients understood how to fill out questionnaire. If not explicitly stated in the questionnaire at what exact time (e.g.: 15:00 instead of 3 h after STT) the gait had to be protocolled, more mistakes were observed. Although some patients did not show any improvement in gait testing the questionnaire demonstrated at least some improvement. Improvement was seen most often in-between 1 h, usually the improvement was gone after 12 h.

**Discussion:** The questionnaire may help to detect NPH patients even if the improvement is under the threshold detectable by gait tests and even if they are not tested at the time of maximum improvement after STT. These advantages must be proven in a prospective study. The questionnaire might reduce the number of patients being withhold from beneficial shunting.

## O52 The modified frailty index to predict morbidity and mortality for shunt surgery in idiopathic normal pressure hydrocephalus

### Teruo Kimura^1^, Adam Tucker^1^, Seiya Fujikawa^1^, Nobuyuki Mitsui^1^, Kazuo Takasugi^1^, Nozomi Suzuki^1^, Shigeo Yoshida^2^

#### ^1^Department of Neurosurgery, Kitami Red Cross Hospital, Hokkaido, Japan; ^2^Department of Internal Medicine, Kitami Red Cross Hospital, Hokkaido, Japan

##### **Correspondence:** Teruo Kimura

*Fluids and Barriers of the CNS* 2018, **15(Suppl 2):**O51

**Introduction:** Frail patients are known to have poor perioperative outcome. We hypothesized that our modified frailty index (mFI) may be a predictor of morbidity and mortality following shunt surgery in idiopathic normal pressure hydrocephalus (iNPH).

**Methods**: A retrospective cohort study was conducted among patients diagnosed with probable iNPH according to the 2004 Guideline for iNPH, who underwent shunt surgery, and were followed 3 years postoperatively between 2005 and 2014. Our mFI, ranging from 0 to 1, was calculated as the proportion of 15 possible risk factors (including dementia, movement disorder, neoplasm and depression). We examined the associations among mFI, Charlson comorbidity index, and 3-year morbidity, mortality, neurological and medical complications by mRS and iNPH grading scale using univariate and multivariable analyses and compared the index to established risk stratification methods.

**Results**: A total of 82 patients were identified. Morbidity, mortality and severe medical complications increased incrementally with increasing level of frailty. Severe neurological complications were highest in those with low frailty. Multivariate logistic regression analysis showed that increased frailty increased the odds of all adverse outcome, including neurological complication. The mFI enhanced the ability to predict 3-years outcomes beyond available indices and was a reliable predictor of neurological complications.

**Conclusions**: This index may be a useful preoperative risk assessment method with implications for shared decision-making, perioperative planning, and risk adjusted outcomes measurement in iNPH patients.

## O53 Influence of cerebrospinal fluid movement on behavior of different substances in cerebrospinal and interstitial fluid

### Marijan Klarica^1^, Milan Radoš^1^, Ivana Jurjević^1^, Darkov Orešković^2^

#### ^1^Department of Pharmacology and Croatian Institute for Brain Research, School of Medicine University of Zagreb, Zagreb, Croatia; ^2^Ruđer Bošković Institute, Department of Molecular Biology, Zagreb, Croatia

##### **Correspondence:** Marijan Klarica

*Fluids and Barriers of the CNS* 2018, **15(Suppl 2):**O52

**Introduction:** Cerebrospinal fluid (CSF) movement is not a unidirectional circulation, but the permanent rhythmic systolic-diastolic pulsation of CSF volume. That kind of CSF movement represents the main force of substances distribution along the CSF system, and from CSF via peri- and paravascular routes into the central nervous system (CNS) parenchyma interstitial space.

**Methods:** Distribution of various substances (radioactive water, organic acids, inulin etc.) has been investigated in CSF and interstitial space after their application into different CSF compartments in freely moving large animals (dogs and cats).

**Results:** Our results suggest that the distribution of substances along the CSF system is observed in all directions (in the direction of imagined circulation from brain ventricles to subarachnoid space as well as in the opposite direction), and depends on the resident time of the substance, whereby longer resident time means longer path of distribution. Quick disappearance of organic anions from central CSF compartments, and very low concentrations of the tested substances in “peripheral” CSF compartments were observed in control condition. When active transport was blocked in animals by probenecid, the concentrations of monitored substances in CNS tissue and in “peripheral” CSF compartments were significantly increased in comparison to the control group.

**Conclusions:** Obtained results suggest that transport at the capillary endothelium has the most important role in CNS homeostasis and in removal of potentially toxic metabolites from the CNS tissue into blood circulation, and not, as was previously conceived, the unidirectional CSF circulation and its absorption through the dural sinuses.

## O54 Imaging in idiopathic normal pressure hydrocephalus: longitudinal changes of radiological signs

### Karin Kockum^1^, Johan Virhammar^2^, Katrin Riklund^3^, Lars Söderström^1^, Elna-Marie Larsson^4^, Katarina Laurell^1^

#### ^1^Department of Pharmacology and Clinical Neuroscience, Unit of Neurology, Östersund, Umeå University, Sweden; ^2^Dept of Neuroscience, Neurology, Uppsala University Hospital, Sweden; ^3^Department of Radiation Sciences, Diagnostic Radiology, Umeå University, Umeå, Sweden; ^4^Department of Surgical Sciences, Radiology, Uppsala University, Sweden

##### **Correspondence:** Karin Kockum

*Fluids and Barriers of the CNS* 2018, **15(Suppl 2):**O53

**Introduction:** The progression of iNPH symptoms is well established, whereas the knowledge of the development of neuroradiological signs over time is more restricted. The aim of this study was to investigate whether elderly with many symptoms of iNPH aggravate more in iNPH-associated radiological changes, compared to elderly with few symptoms of iNPH.

**Methods:** This study is part of a prospective, population-based prevalence study carried out between September 2014 and August 2015, previously described by our group. As a 2-year follow-up, the 168 participants were re-invited for a new CT scan of the brain, conducted between April and October 2017, where 118 completed both imaging and neurological examination. Symptom assessment at baseline was made according to the NPHscore by Hellström. (1) One radiologist, blinded to clinical data, scored the seven parameters summarized in the recently published iNPH Radscale score (2) including Evans’ index, temporal horn width, callosal angle, periventricular hypodensities, compressed sulci, focally dilated sulci and widening of the Sylvian fissures.

**Results:** Interim data of the first 88 consecutive investigations show that there was a small but statistically significant increase in iNPH Radscale score, comparing baseline and follow up. The association between the symptom score at baseline and the difference in radiological score was nonsignificant.

**Conclusion:** Radiological features of iNPH gently progress over time in elderly, independent symptom severity at baseline. The imaging findings need to be correlated to symptom progression to define its clinical relevance.

**References:**

1. Hellstrom P, Klinge P, Tans J, Wikkelso C. A new scale for assessment of severity and outcome in iNPH. Acta Neurol Scand. 2012;126(4):229–37.
2. Kockum K, Lilja-Lund O, Larsson EM, Rosell M, Soderstrom L, Virhammar J, et al. The idiopathic normal-pressure hydrocephalus Radscale: a radiological scale for structured evaluation. Eur J Neurol. 2018;25(3):569–76.

## O55 The role of third ventricle bowing in the success of endoscopic third ventriculostomy in pediatric and adult patients

### Tomáš Krejčí^1,2^, Ondřej Krejčí^1^, Radim Lipina^1,2^

#### ^1^Department of Neurosurgery, University Hospital Ostrava, Czech Republic; ^2^Faculty of Medicine, University of Ostrava, Czech Republic

##### **Correspondence:** Tomáš Krejčí

*Fluids and Barriers of the CNS* 2018, **15(Suppl 2):**O54

**Background:** The presence of third ventricle deformation, otherwise known as ‘bowing’, is an indicator of obstructive hydrocephalus and also the success of endoscopic third ventriculostomy (ETV). The aim of this study is to determine bowing’s influence in ETV success in both pediatric and adult patients and is the first study to evaluate this relationship in patients younger than 6 months old.

**Method:** 157 ETVs were performed on 157 patients with obstructive hydrocephalus between January 2008 and December 2016. The presence and extent of third ventricle floor bowing was determined by preoperative MR imaging. Only patients who had been followed up for at least 3 months or those in which it was possible to determine third ventricle bowing were included in the study. 135 patients (70 adults and 65 children of which 38 were younger than 6 months old) fulfilled the induction criteria. Additional factors were evaluated for their effects on ETV success.

**Results:** In patients older than 6 months, The ETV success rate was 91% in those presenting with bowing and 47.6% in those without. Among patients younger than 6 months old, ETV was successful in 37% of those with bowing and 36.4% of those without. We confirmed that the presence of bowing strongly indicates ETV success in patients older than 6 months (p < 0.0005), including those of 7 months of age and older (p 0.001). However, this relationship was not confirmed in pediatric patients up to 6 months old (p 1.000). Additionally, there is a greater risk of failure among this group (p 0.002). The extent of bowing, as opposed to its presence, does not influence ETV success (p 0.559). Bowing correction strongly correlates with ETV success (p < 0.0005), being observed in 96% of patients who successfully underwent ETV. While bowing more frequently occurs in severely premature infants (p 0.049), prematurity in itself does not affect ETV outcome (p 0.262). We confirmed the relationship between intraventricular hemorrhage and ETV failure (p < 0.0005) but among patients older than 6 months, ETV success was not found to be affected by the etiology of hydrocephalus (p 0.527), clinical problems (p 0.115) or clinical duration (0.546). 90% of ETV failures occurred within 12 weeks post operation whereas just 3.4% of ETV failures occurred more than 1 year post operation; ETV failure is significantly more likely in the first 12 postoperative weeks (p < 0.0005).

**Conclusions:** Our study is the largest published discussing bowing as an indicator of ETV success and is to date the only one to investigate this relationship in patients younger than 6 months old. While we confirmed significant correlation between bowing and ETV success in both adults and children over 6 months of age, this relationship was not determined in those younger than 6 months and therefore we do not recommend bowing as one of the ETV indication criteria for this patient cohort.

## O56 Cerebrospinal fluid pressure sagittal sinus pressure and Davson’s equation in idiopathic intracranial hypertension

### Afroditi Lalou^1^, Marek Czosnyka^1^, Zofia Czosnyka^1^, Deepa Krishnakumar^2^, John Douglas Pickard^1^

#### ^1^Academic Neurosurgery, Division of Neurosurgery, Department of Clinical Neurosciences, University of Cambridge, United Kingdom; ^2^Paediatric Neurology, Department of Paediatrics, Cambridge University Hospitals NHS Foundation Trust, Hills Road, Cambridge, United Kingdom

##### **Correspondence:** Afroditi Lalou

*Fluids and Barriers of the CNS* 2018, **15(Suppl 2):**O55

**Introduction**: We have previously demonstrated a coupling between CSF pressure (CSFp) and sagittal sinus pressure (SSp) in 10 IIH patients. When investigating CSF and venous circulation in IIH, little is known about the relationship between CSF and SSp. In this study, we aimed at investigating the CSF dynamics and physics behind this coupling. Davson’s equation describes the relationship between SSp and CSF circulation in physiological individuals, however requires investigation in CSF disorders. We are proposing a re-arrangement of Davson’s equation for IIH patients based on our current findings.

**Materials and methods:** After investigating the results of SSp and CSFp coupling in IIH, we sought to interpret its implications on Davson’s equation in these patients. We compared our IIH patients, with known SSp, with 10 normal controls. Based on our findings, we estimated the resistance to CSF outflow (Rout) using a correction derived from linear regression between the CSFp and SSp.

**Results:** The coupling between SSp and CSFp resolved after drainage of CSF below a specific CSFp, estimated to be the jugular venous pressure (JVP). Davson’s equation can therefore be rearranged based on these results as CSFp = Rout *If + a*CSFp + b where a calculated as 0.70 ± 0.14 for nine patients and b was calculated as 6.3 ± 3.53 mmHg and represents the intercept of this correlation, which physiologically should correspond to JVP.

**Conclusions:** During CSF drainage, both CSFp and JVP decrease until a certain point (most probably JVP) when CSFp can decrease further while SSp remains constant. Davson’s equation can be simplified in IIH using our proposed formula. Rout calculated during infusion test without measurement of SSp is overestimated, and does not have the same meaning like in hydrocephalus.

## O57 The value of computerised shunt infusion studies in suspected shunt malfunction in paediatric hydrocephalus - a two centre observational study

### Sandra Dias^1,2^, Afroditi Lalou^3^, Susanne R. Kerscher^1^, Karin Haas-Lude^4^, Matthew Garnett^3^, Helen Fernandes^3^, Marek Czosnyka^3^, Zofia Czosnyka^3^, Martin U. Schuhmann^1^

#### ^1^Division of Pediatric Neurosurgery, Department of Neurosurgery, University Hospital of Tübingen, Germany; ^2^Department of Neurosurgery, University Hospital of Zurich, Switzerland; ^3^Academic Neurosurgery Unit, University of Cambridge School of Clinical Medicine, Addenbrooke’s Hospital, Cambridge, United Kingdom; ^4^Department of Pediatric Neurology, University Children’s Hospital Tübingen, Germany

##### **Correspondence:** Afroditi Lalou

*Fluids and Barriers of the CNS* 2018, **15(Suppl 2):**O56

**Introduction:** Hydrocephalus shunt malfunction can—especially in children—occur insidiously without symptoms of raised intracranial pressure (ICP) or changes in ventricular size. It therefore imposes a diagnostic challenge. Xray-based “shuntograms” are, if at all, qualitative and imply radiation exposure. Computerised shunt-infusion-studies (SIS) enable quantitative shunt function assessment. We report on feasibility and results of this technique.

**Materials and methods:** Dedicated software (ICM+) containing the shunt’s resistance characteristics, calculates baseline and plateau ICP, ICP-amplitudes, elastance, outflow resistance and critical shunt pressure (CSP = maximum plateau pressure at normal shunt resistance). 192SIS (39 in Tubingen and 153 in Cambridge) were performed in 159 children. Functional shunts were defined by ICPplateau > 1 mmHg below CSP, non-functional > 1 mmHg above CSP and borderline in between. Overall, 38 (20%) of shunt were obstructed, 27 (14%) borderline and 127 (66%) were functional.

**Results:** Baseline ICP in obstructed shunts was significantly above shunt operating pressure. CSF outflow resistance and ∆ICP plateau were significantly elevated (n = 0.001) in obstructed shunts, with cut-off thresholds being 5.9 mmHg/mL/min and 8.5 mmHg, respectively. All obstructed shunts were revised. In about 50% of cases the ventricular size decreased postoperatively, with 50% of patients showing an improvement on clinical investigation or according to parent’s reports.

**Conclusions:** SIS is a feasible, elegant and radiation free technique for quantitative shunt assessment to rule out or prove shunt malfunction. Dedicated software containing shunt hydrodynamic characteristics is necessary and small children will need short term sedation. Due to his clinical and inherent economic advantages, we postulate that SIS should become routine in neurosurgery units.

## O58 Agreement between detection of shunt blockage: intraoperative versus infusion test results

### Aforditi Lalou^1^, Valzerda E Beqiri^1,2^, Marek Czosnyka^1^, Eva Nabbanja^3^, Matthew R Garnett^3^, Zofia Czosnyka^1^

#### ^1^Academic Neurosurgery, Division of Neurosurgery, Department of Clinical Neurosciences, University of Cambridge, United Kingdom; ^2^Department of pathophysiology and transplantation, University of Milan, Italy; ^3^Division of Neurosurgery, Department of Clinical Neurosciences, Cambridge University Hospital, United Kingdom

##### **Correspondence:** Afroditi Lalou

*Fluids and Barriers of the CNS* 2018, **15(Suppl 2):**O57

**Introduction:** We have previously investigated the negative predictive value of shunt infusion studies and their role in avoiding revisions of properly functioning shunts. With shunt infusion studies we can accurately detect blockage, over/underdrainage or normal function of shunts. We hypothesised that the positive predictive value of shunt infusion studies in detecting shunt obstruction is also high.

**Methods:** We identified a cohort of patients who had their shunts tested right before revision surgery and compare the two findings. A first researcher collected the results of infusion tests showing blockage between January 2013 and December 2017. A second, independent researcher collected the results from the revision of the shunt. The second researcher was blinded to the infusion results. The findings were compared and contrasted.

**Results:** A total of 47 patients (29female:18male) during this time period had undergone an infusion study confirming blockage with immediate revision afterwards and had a clear intraoperative note describing the patency of all the shunt elements. In 45 of these, the intraoperative finding was identical to the blockage detected during infusion. The two patients in whom there was a disagreement were scrutinised individually and the dispute could be identified in limitations in the intraoperative notes and clinical opinion. 46/47 patients also had confirmation that the other end of the shunt was patent. These correspond to 96% 98% positive predictive value for shunt obstruction and shunt patency identification respectively.

**Conclusions:** Infusion studies are an objective, accurate method that can be used to identify shunt blockage and/or confirm shunt patency at their occurrence, as well as the exact site of the blockage (proximally or distally to the needle insertion).

## O59 Effects of shunt function on ventricular volume in idiopathic normal pressure hydrocephalus

### Simon Lidén^1^, Dan Farahmand^2^, Johan Virhammar^3^, Elna-Marie Larsson^4^, Katarina Laurell^1^

#### ^1^Department of Pharmacology and Clinical Neuroscience, Umeå University, Sweden; ^2^Department of Clinical Neuroscience, Institute of Neuroscience and Physiology, The Sahlgrenska Academy, University of Gothenburg, and Department of Neurosurgery, Sahlgrenska university Hospital, Sweden; ^3^Department of Neuroscience, Neurology, Uppsala University Hospital, Sweden; ^4^Department of Surgical Sciences, Radiology, Uppsala University, Sweden

##### **Correspondence:** Simon Lidén

*Fluids and Barriers of the CNS* 2018, **15(Suppl 2):**O58

**Introduction:** Currently, non-invasive methods to evaluate the shunt effect at different shunt valve settings in hydrocephalus is lacking. A possible future method to evaluate shunt function could be volumetric measurement of brain ventricles. This study aimed to examine whether the ventricular volume changes in response to shunt treatment, and at different setting of the shunt valve.

**Methods:** Consecutive patients undergoing shunt-surgery were invited to participate in the study. Exclusion criteria were severe cognitive impairment (Mini Mental State Examination < 15). Using MRI volumetry (SyntheticMR^®^) the effects of three different settings of strata shunts on ventricular volume were examined: low (1.0), intermediate (1.5) and high (2.5) valve setting. Each setting was tried in all patients, beginning with intermediate followed by a randomized and blinded order of low and high. MRI was performed 1 month after each shunt adjustment.

**Results:** The four patients who have so far completed the study (men = 4, mean age = 78, SD = 5.5) showed a significant decrease in ventricular volume between preoperative and first as well as third post-operative MRI measurements with mean 139.8/125.5/119.8 mL, respectively (SD = 21.67/21.71/15.82, p < 0.05). Individual changes in volume in relation to different valve settings will be presented.

**Conclusions:** The ventricular volume changed significantly after shunt surgery. Volumetry seems to be a promising future method for shunt function evaluation.

## O60 Cerebral blood flow and oxygen metabolism as a new diagnostic for infant hydrocephalus in both the developed and developing worlds

### Lin Pei-Yi^1^, Sutin D. B. Jason^1^, Cheng Fang-Yu^2^, Vyas Rutvi^1^, Ssenyonga Peter^3^, Mbabazi Edith^3^, Blackwell Sarah^1^, Chimileski Brock^1^, Franceschini Maria Angela^3^, Grant Ellen Patricia^1^, Warf C Benjamin^4^

#### ^1^Fetal-Neonatal Neuroimaging and Developmental Science Center, Boston Children’s Hospital/Harvard Medical School, United States; ^2^Athinoula A. Martinos Center for Biomedical Imaging, Massachusetts General Hospital/Harvard Medical School, Charlestown, United States; ^3^CURE Children’s Hospital of Uganda, Mbale, Uganda; ^4^Departments of Neurosurgery and Global Health and Social Medicine, Boston Children’s Hospital and Harvard Medical School, United States

##### **Correspondence:** Lin Pei-Yi

*Fluids and Barriers of the CNS* 2018, **15(Suppl 2):**O59

**Introduction:** Current assessments of the progression of infant hydrocephalus and its treatment are crude, with little power for predicting neurodevelopmental impairment (NDI). We developed novel frequency-domain near-infrared (FDNIRS) and diffuse correlation spectroscopy (DCS) for quantitative measurement of cerebral blood flow (CBF) and oxygen metabolism (CMRO_2_) non-invasively, at the bedside, in both the developed and developing worlds. We aim to test whether CBF and CMRO_2_ can be new indicators of cerebral health for guiding hydrocephalus treatment and NDI prognosis.

**Methods:** We are investigating post-hemorrhagic hydrocephalus (PHH) and spinal bifida at Boston Children’s Hospital (BCH), and post-infectious hydrocephalus (PIH) infants at CURE Children’s Hospital Uganda (CCHU). Primary study outcomes are CBF and CMRO_2_ with secondary outcomes of cerebral hemoglobin concentrations, treatment outcomes (success or failed within 6 months), and brain growth in 6-month post-operative brain imaging scans.

**Results:** In the BCH cohort (N = 17), we found successful hydrocephalus treatment immediately increases CBF and restores CMRO_2_, whereas there was no change after unsuccessful treatment. The CCHU cohort (N = 33) shows primary infectious injuries in PIH cause more severe damage to brain structure than in PHH. Decreases in brain optical scattering immediately post-surgery is highly predictive for treatment failure to occur within 6 months. Most importantly, brain regions with higher CMRO_2_ had better recovery of brain structure by CT scan 6 months after treatment.

**Conclusions**: We have demonstrated our measurements of cerebral physiology are sensitive to the state of hydrocephalus and have potential as objective quantitative diagnostics for both high and low resource settings.

## O61 Impact of resistance to CSF outflow on CSF dynamic and ventricular morphometry

### Armelle Lokossou^1^, Zofia. Czosnyka^2^, Pan Liu^1^, Alexandra Vallet ^3^, Laurent Balardy^4^, Pierre Payoux^3,5^, Olivier Balédent^1,6^, Eric. A. Schmidt^3,7^

#### ^1^CHIMERE EA 7516, Research Team for head&neck,  niversity of Picardie Jules Verne, Amiens, France; ^2^Department of Neurosciences, University of Cambridge, United Kingdom; ^3^UMR 1214 - INSERM/UPS – TONIC Toulouse Neuro-Imaging Center, Toulouse, France; ^4^Department of Geriatry, University Hospital, Toulouse, France; ^5^Department of Nuclear Medicine, University Hospital Toulouse, France; ^6^Department of Image processing, University Hospital, Amiens, France; ^7^Department of Neurosurgery, University Hospital, Toulouse, France

##### **Correspondence:** Armelle Lokossou

*Fluids and Barriers of the CNS* 2018, **15(Suppl 2):**O60

**Introduction:** It is still debated that aqueductal cerebrospinal fluid (CSF) dynamic correlates with ventricular morphometry in hydrocephalus patients. Resistance to CSF outflow (Ro) obtained by infusion tests during intracranial pressure monitoring is the gold standard to evaluate CSF disorders and to select shunting patients. We determined the impact of Ro on the interplay between CSF dynamic and ventricular morphometry in hydrocephalus condition.

**Methods:** 79 Hydrocephalus patients (75 ± 7 years) underwent infusion tests to evaluate Ro using ICM+. The previous day, they had morphological MRI sequences to quantify ventricular volume and PCMRI sequence to assess aqueductal CSF flows oscillations. A Ventricular Volume index (VVi) was calculated by measuring the lateral ventricles area on a single 2D Flair axial image passing through the splenium and the genu of corpus callosum. This index was normalized by the head circumference. These measurements were made using ImageJ software. PCMRI data were analyzed with homemade software *Flow* to extract aqueductal CSF stroke volume (ASV) which represents the volume of CSF moving through the aqueduct during cardiac cycle.

Based on Ro value, we classified patients in potential-non-responders (PNR, n = 33) and potential-responders (PR, n = 46) groups to shunt surgery. Then, Pearson correlations were calculated between ASV and VVi.

**Results:** There was a significant moderate correlation between ASV and VVi in all hydrocephalus patients (r = 0.53, p < 0.0001), a significant correlation in PR patients (r = 0.64, p < 0.0001) and a weak and non-significant correlation in PNR patients (r = 0.25, p > 0.05).

**Conclusion:** Only patients with high Ro had a significant association between aqueductal CSF dynamic and ventricular morphometry.

## O62 Drainage, irrigation and fibrinolytic therapy (drift) for posthaemorrhagic ventricular dilatation in preterm infants; health-economic evaluation at 10-years

### William Hollingworth^1^, David Odd^1^, Sally Jary^1^, Charlotte Lea^1^, Aida Moure Fernandez^1^, Grace Young^1^, Pete Blair^1^, Cathy Williams^1^, Ian Pople^2^, Kristian Aquilina^3^, Michelle Morgan^4^, Andrew Whitelaw^1^, Karen Luyt^1,2^

#### ^1^Department of Neonatal Neurology, University of Bristol, Bristol, United Kingdom; ^2^Department of Neonatal Intensive Care, University Hospitals NHS Trust, Bristol, United Kingdom; ^3^Department of Paediatric Neurosurgery, Great Ormond Street Hospital, London, United Kingdom; ^4^Department of Paediatric Psychology, Community Children’s Health Partnership, Bristol, United Kingdom

##### **Correspondence:** Karen Luyt

*Fluids and Barriers of the CNS* 2018, **15(Suppl 2):**O61

**Introduction:** Drainage, irrigation and fibrinolytic therapy (DRIFT) is a novel method to clear the effects of intraventricular haemorrhage (IVH) with post-haemorrhagic ventricular dilatation (PHVD). DRIFT is the first intervention to reduce severe cognitive disability after PHVD. Our aim was to determine whether the initial costs of the intervention were offset by long term savings.

**Methods:** 10-year follow up of an RCT comparing DRIFT with standard care. Economic evaluation of 54 children. We used micro-costing to estimate the cost of DRIFT. Inpatient care information was extracted from hospital notes and hospital episode statistics data. Parental questionnaires provided data on other health, social and educational resource use. Regressions, adjusting for sex, birth weight and IVH grade were employed to estimate differences in mean costs between DRIFT and standard care.

**Results:** Participants had DRIFT for an average of 5.2 days (estimated cost £1513 per participant). We found no definitive evidence that DRIFT increased or decreased neonatal or other hospital costs up to 10 years, or ambulatory health and social care at 10 years. Results were sensitive to adjustment for sex and weight. Fewer participants in the DRIFT arm attended a special unit/school (adjusted odds ratio [95%CI] 0.13 [0.02, 0.82]). Due to the high cost of special education, this is potentially economically important; the adjusted mean [95%CI] difference in estimated annual school costs was − £5321 [− £9772, − £870].

**Conclusions:** DRIFT has a moderate financial cost, but the economic consequences are potentially very large, particularly if it reduces the need for special education in the long-term.

## O63 The difficult task of creating an expert centre dedicated to CSF disorders: from dream to real life

### Romain Manet^1^, Giorgio Palandri^2^, Virginie Desestret^3^, Zofia Czosnyka^4^, Marek Czosnyka^4^, Laurent Gergelé^4,5^, Emmanuel Jouanneau^1^

#### ^1^Department of Neurosurgery B, Neurological Hospital Wertheimer, University of Lyon, France; ^2^Department of Neurosurgery, Institute of Neurological Sciences of Bologna, Bellaria Hospital, University of Bologna, Italy; ^3^Department of Neurology D, Neurological Hospital Wertheimer, University of Lyon, France; ^4^Division of Neurosurgery, Department of Clinical Neurosciences, Addenbrooke’s Hospital, University of Cambridge, United Kingdom; ^5^Department of Intensive Care, Ramsay Générale de Santé, Hôpital privé de la Loire, Saint Etienne, France

##### **Correspondence:** Romain Manet

*Fluids and Barriers of the CNS* 2018, **15(Suppl 2):**O62

**Introduction:** Diagnosis, treatment and follow-up of cerebrospinal fluid (CSF) disorders are often controversial and challenging. Ideally, they should be managed in dedicated expert centres and follow consensual guidelines. However, to date, expert centres and guidelines are scarce. In this collaborative work, we wanted to identify challenges in building a dedicated centre for CSF disorders. Our intention is to share our experience, in order to promote such project and rise awareness of the experts community on the compelling needs for producing consensual, updated and unified guidelines to manage CSF disorders.

**Materials and methods:** The study consisted in three steps: (I) Listing the difficulties we encountered when starting to create a centre dedicated to CSF disorders; (II) Reviewing published clinical guidelines; (III) Visiting renowned European expert centres to evaluate their structural organization.

**Results**: (I) When starting to create our centre for CSF disorders, we encountered difficulties in: motivating busy colleagues to create a multidisciplinary team, dealing with patients hosting in a financially stressed system, getting access to neuropsychological assessment, standardizing patients evaluation, proving medico-financial interest to the administration of our institution. (II) Three different clinical guidelines have been published concerning normal pressure hydrocephalus (NPH). To date, no guidelines have been published concerning other CSF disorders. (III) We described the modalities we adopted from the different European expert centres we visited.

**Conclusions**: Creating an expert centre dedicated to CSF disorders is probably valuable but remains challenging.

## O64 Neurocognitive outcome in patient submitted to third ventricle cysternostomy for hydrocephalus due to idiopathic aqueductal stenosis in adults

### Matteo Martinoni^1^, Luca Albini Riccioli^2^, Francesca Santoro^3^, Paolo Mantovani^1^, Giacomo Bertolini^1^, Alessandro Pirina^1^, Marco Di Gino, Andrea Cuoci^1^, Mariella Lefosse^1^, Francesca Nicolini^1^, Mino Zucchelli^1^, Carmelo Sturiale^1^, Giorgio Palandri^1^

#### ^1^Department of Neurosurgery, IRCCS-ISNB, Bellaria Hospital, Bologna, Italy; ^2^Department of Neuroradiology, IRCCS-ISNB, Bellaria hospital, Bologna, Italy; ^3^Department of Neurology, IRCCS-ISNB, Bellaria Hospital, Bologna, Italy

##### **Correspondence:** Matteo Martinoni

*Fluids and Barriers of the CNS* 2018, **15(Suppl 2):**O63

**Introduction:** Idiopathic aqueductal stenosis (IAS) of the adult is an obstruction of cerebrospinal fluid outflow causing triventricular hydrocephalus. Clinical presentation mostly includes chronic neurological symptoms and cognitive inefficiencies. Endoscopic third ventriculostomy (ETV) represents the treatment of choice of this condition. Postoperative neurocognitive outcome has been poorely investigated.

**Methods:** All patients affected by IAS and submitted to ETV were prospectively included in this study. Partecipants were administered a tailored neuropsychological testing battery to assess intellectual functioning, visual and verbal memory, language skills, and executive functioning pre and post operatively. All patients were clinically evaluated both pre and post operatively. Pre and post operative MRI findings were matched with neurocognitive outcome.

**Results:** Between 2015 and 2017 eleven patients met the inclusion criteria. Median age of the patients was 59 (23 min–73 max). In two patients clinical presentation was headache, in one case papilledema without other symptoms and eight patients presented the typical symptoms of chronic hydrocephalus. All patients but one clinically improved after ETV. Postoperative neurocognitive evaluation compared to the preoperative one evidenced that 8 patients improved in different domains, one patient didn’t obtain any benefit from the procedure and two patients worsened in visuospatial memory domains. No patient died and no case of severe morbidity occurred.

**Conclusions:** In our series ETV has confirmed to be a safe and effective treatment leading to neurological improvement almost in all cases. Also cognitive inefficiencies seem to benefit from surgical treatment however these results are more variable and difficult to be interpreted and further studies are warrented.

## O65 Towards visualization of bulk flow of cerebrospinal fluid based on Q-space imaging

### Kenta Maruyama^1^, Takuma Okada^1^, Mitsunori Matsumae^2^, Hideki Atsumi^2^, Kagayaki Kuroda^1,3^

#### ^1^Course of Electrical and Electronic Engineering Graduate School of Engineering, Tokai University, Kanagawa, Japan; ^2^Department of Neurosurgery, Tokai University, Kanagawa, Japan; ^3^Department of Human and Information Sciences School of Information Science and Technology, Tokai University, Kanagawa, Japan

##### **Correspondence:** Kenta Maruyama

*Fluids and Barriers of the CNS* 2018, **15(Suppl 2):**O64

**Introduction:** Visualization of microscopic CSF motion called “bulk flow” may lead us to explicate clearance activity of neural wastes in the brain. In this work, we examined a quantitative visualization of microscopic motion based on q-space imaging (QSI) obtained with MRI.

**Methods:** A phantom with microscopic circulation of physiological saline through a tube of 6 mm in inner diameter was placed in a vertical 9.4-T MR scanner. Flow rate of the pump was set at 0.1–0.5 mL/min with 0.1 mL/min steps. Strength of motion probing gradient (MPG) was changed from − 43.4 to + 43.4 mT/m with 6.2 mT/m steps. The direction of MPG was foot-head direction. This resulted in a q-space of 32 points at each voxel in an axial section. The q-space was then Fourier-transformed into a probability density function (PDF) of proton displacement. The peak of PDF indicated the proton flow. The spatial distribution of the flow velocity was obtained by dividing the displacement by the MPG interval at all voxels.

**Results:** The resultant flow velocities were from 57.80 to 300.72 µm/s, and were highly correlated (r = 0.99, p < 0.01) with the velocities generated by the pump. Regardless of the presence or absence of flow, the diffusion coefficient was about 1.6 × 10^−9^ m^2^ at each flow rate.

**Conclusions:** Microscopic flow of the order of several 10 µm/s was sufficiently quantified by the present technique, even when the self-diffusion exists. Our experimental work to observe the CSF bulk flow in mouse brain is in progress.

## O66 Turnover of water molecules in brain, ventricles and subarachnoid spaces in normal volunteers and patients with idiopathic NPH: dynamic pet study using H_2_^15^O

### Mitsuhito Mase^1^, Emi Hayashi^2^, Shin Hibino^2^, Yoshiya Ito^2^, Akihiko Iida^2^, Toshiaki Miyati^3^, Etsuro Mori^4^

#### ^1^Department of Neurosurgery, Nagoya City University Graduate School of Medical Sciences, Nagoya, Japan; ^2^Department of Radiology, Nagoya City Rehabilitation Center, Nagoya, Japan; ^3^Faculty of Health Science, Institute of Medical, Pharmaceutical and Health Sciences, Kanazawa University, Kanazawa, Japan; ^4^Department of Behavioral Neurology and Neuropsychiatry, Osaka University United Graduate School of Child Development, Osaka, Japan

##### **Correspondence:** Mitsuhito Mase

*Fluids and Barriers of the CNS* 2018, **15(Suppl 2):**O65

**Introduction:** In order to clarify the origin and turnover of water molecules in CSF, dynamic PET (positron emission tomography) study was performed using radio labeled H_2_O.

**Methods:** Normal volunteers (n = 10) and patients with definite idiopathic normal pressure hydrocephalus (iNPH, n = 5) were included. Dynamic PET data were obtained for 15 min after intravenous injection of saline including H_2_^15^O (500 MBq). Voxels of interest (VOI) were set in the internal carotid artery (ICA), superior sagittal sinus (SSS), choroid plexus (CP), cortical gray matter (GM), white matter (WM), basal ganglia (BG), lateral ventricle (LV), Sylvian fissure (FS), and prepontine cistern (PPC). Time and relative radio activity (RAA) curves of each VOI were analyzed.

**Results:** The maximum peak radio activities of GM, WM and BG were at 22.5, 50.0 and 22.5 s after the peak in ICA, respectively. The activities in the whole brain structures decreased gradually. On the contrary, the activity of LV, FS and PPC increased gradually until the end of the measurement {13.6, 27.4, and 50.9% of the whole brain parenchyma (GM + WM + BG) activity at 9.5 min, respectively}. The RRA of LV, FS and PPC in iNPH tended to be lower compared to the normal control. After L-P shunt surgery, RRA of these subarachnoid spaces tend to increase compared to the preoperative values.

**Conclusions:** The present study showed very fast movement of water molecules from artery to brain parenchyma and ventricular and subarachnoid CSF. Water movements into the subarachnoid spaces may delay in iNPH patients, which may be normalized after shunt surgery.

## O67 Changing the currently held concept of cerebrospinal fluid dynamics-assessed by mutual findings of cerebrospinal fluid motion in the CSF space using various types of MRI techniques

### Mitsunori Matsumae^1^, Kagayaki Kuroda^2^

#### ^1^Department of Neurosurgery, Tokai University School of Medicine, Isehara, Kanagawa, Japan; ^2^Human and Information Sciences, School of Information Science and Technology, Tokai University, Hiratsuka, Kanagawa, Japan

##### **Correspondence:** Mitsunori Matsumae

*Fluids and Barriers of the CNS* 2018, **15(Suppl 2):**O66

**Object**: It has been widely recognized that cerebrospinal fluid (CSF) is produced by the choroid plexus and travels caudally through the ventricular system, circulates within the spinal cord and over the surface of the brain, and is eventually absorbed by the arachnoid granulations and villi near the venous sinuses, returning to the bloodstream. However, through subsequent magnetic resonance imaging (MRI) studies in humans and in animals, many researchers have raised questions regarding this “CSF circulation theory” of CSF flowing unidirectionally and circulating through the ventricles and subarachnoid space in a downward or upward fashion.

**Methods**: In this manuscript, we describe observations of CSF motion using three different MRI techniques, extract findings that are common among these techniques, and discuss CSF motion, as we currently understand it based on the results from the quantitative analysis of CSF motion.

**Results**: CSF moves in various directions in the ventricles and subarachnoid space (CSF space), and the velocity of CSF motion differs depending on the CSF space.

**Conclusion**: It is necessary to revise the currently held concept that CSF flows unidirectionally.

## O68 Preliminary results on the effects of a cell junction inhibitor in experimental post-hemorrhagic hydrocephalus

### James P. McAllister II^1^, Leandro Castaneyra-Ruiz^1^, Albert M. Isaacs^1^, Alexis Hartman^1^, Joel Brown^1^, Diego M. Morales^1^, Travis S. Crevecoeur^1^, Xia Ge^2^, John A. Engelbach^2^, Joel Garbow^2^, Sheng-kwei Song^2^, David D. Limbrick Jr^1^

#### ^1^Department of Neurosurgery, Division of Pediatric Neurosurgery, Washington University School of Medicine, St. Louis, United States; ^2^Department of Radiology, Mallinckrodt Institute of Radiology, Washington University School of Medicine, St. Louis, Missouri, United States

##### **Correspondence:** James P. McAllister II

*Fluids and Barriers of the CNS* 2018, **15(Suppl 2):**O67

**Introduction:** Our previous observations on human neuropathology specimens and cell cultures correlate well with our finding in infant ferrets of ventricular zone (VZ) disruption following intraventricular hemorrhage and post-hemorrhagic hydrocephalus (PHH). Impairments of ependymal cell (adherens) junctions appear to be a common feature of this disruption, which is mediated in part by ADAM10 modulation of N-cadherin cell junctions. Building on promising in vitro data, we sought to determine if an ADAM10 inhibitor could ameliorate VZ disruption and ventriculomegaly in our infant ferret model of PHH.

**Methods:** PHH was induced in 20-day old ferrets by intraventricular injections of heparinized autologous or syngeneic lysed blood; sham controls received sterile saline. The ADAM10 inhibitor was given daily for 11 days post-induction. Anatomic and diffusion MRI evaluations were conducted biweekly until approximately 70 days post-induction. Fixed tissue was analyzed using immunohistochemistry for ependymal cells, neural progenitors, multiciliated ependymal cells, astrocytes, cell-adhesion molecules, N-cadherin, and ADAM10.

**Results:** To date, 24 PHH and five control ferrets were analyzed. Within 1–2 weeks after PHH induction, mild-moderate ventriculomegaly occurred predominantly in the occipital and inferior horns. PHH animals, exhibited intermittent patches of disrupted VZ, increased expression of ADAM10, decreased expression of N-cadherin, and astrocytosis in periventricular white matter (PVWM). Diffusion MRI, especially diffusion basis spectrum imaging (DBSI) revealed myelin disruption, edema, axon loss and neuroinflammation in PVWM. Most importantly, no ventriculomegaly occurred during ADAM10-inhibitor treatments.

**Conclusions:** VZ disruption and periventricular white matter astrocytosis occur in PHH and may be prevented by treatment with cell junction inhibitors.

## O69 Biocompatibility of the novel microbot medical SCS shunt catheter

### James P McAllister II^1^, Leandro Castaneyra-Ruiz^1^, Albert M Isaacs^1^, Michael Talcott^1,2^, Simon Sharon^3^, Or Samoocha^3^, Eran Cohen^3^, Ian Boader^3^, Carolyn A Harris^4^, Hezi Himelfarb^3^

#### ^1^Department of Neurosurgery, Division of Pediatric Neurosurgery, Washington University School of Medicine, St. Louis, United Sates; ^2^Division of Comparative Medicine, Washington University School of Medicine, St. Louis, United Sates; ^3^Microbot Medical Ltd, Caesarea, Israel; ^4^Department of Chemical Engineering, Wayne State University, Detroit, United Sates

##### **Correspondence:** James P McAllister II

*Fluids and Barriers of the CNS* 2018, **15(Suppl 2):**O68

**Introduction:** Since occlusion of ventriculoperitoneal shunt (VPS) catheters continues to be a major cause of treatment failure in hydrocephalus patients, Microbot Medical, Inc. has designed the self-cleaning ventricular catheter (SCS) to resist catheter obstruction. Following promising in vitro and ex situ studies, preclinical evaluations of the SCS are needed, with an initial focus on biocompatibility.

**Methods:** Hydrocephalus was induced in 30-day old piglets by percutaneous intracisternal injection of 25% kaolin. After ventriculomegaly had developed for 3–44 days, standard VPS (n = 1) and SCS (n = 4) catheters were implanted using routine surgical procedures. The Microbot activation coil was placed into a subcutaneous pocket behind the left ear and distal catheters were tunneled subcutaneously and inserted into the peritoneal cavity. Animals were monitored for neurological deficits, ventriculomegaly, and sacrificed at 5–49 days post-shunt (median 15d). Fixed tissue blocks containing the catheter path were processed for histopathology and immunohistochemistry.

**Results:** Preliminary observations indicate that unactivated Microbot catheters exhibit the same neural tissue reaction as the standard commercial catheter in current clinical practice, although ventriculomegaly increased somewhat after shunting (ventricular volume 2774 mm^3^ vs 4051 mm^3^). Both types of catheters were ensheathed by a very thin capsule of collagenous and astrocytic tissue as they passed through brain tissue or contacted the ependyma. Adjacent white matter exhibited a mild inflammatory reaction that extended only 0.2–1.0 mm beyond the surface of the catheter.

**Conclusions:** The inflammatory reaction to the Microbot catheter appeared to be no different than the reaction to the standard catheter.

## O70 Development of a piglet model of hydrocephalus for endoscopic third ventriculostomy

### James P. McAllister II^1^, Michael Talcott^2^, Albert M. Isaacs^1^, Leandro Castaneyra-Ruiz^1^, Alexis Hartman^1^, David D. Limbrick Jr^1^

#### ^1^Department of Neurosurgery, Division of Pediatric Neurosurgery, Washington University School of Medicine, St. Louis, Missouri, United States; ^2^Division of Comparative Medicine, Washington University School of Medicine, St. Louis, Missouri, United States

##### **Correspondence:** James P. McAllister II

*Fluids and Barriers of the CNS* 2018, **15(Suppl 2):**O69

**Introduction:** Large, clinically-relevant animal models of hydrocephalus in which neurosurgical devices and novel procedures could be tested are lacking. To meet this unmet need, we have developed a porcine model of juvenile obstructive hydrocephalus, and now report our experience in performing endoscopic third ventriculostomy (ETV) on this model.

**Methods:** Hydrocephalus was induced in 30-day old piglets by percutaneous intracisternal injections of 25% kaolin. Pre- and post-kaolin, and pre- and post-treatment (ventriculoperitoneal shunting and ETV), anatomic MRIs were obtained to document ventriculomegaly and guide the neurosurgical procedures. Animals survived 1–84 days (median 41) post-kaolin and 17 days (median 18) post-shunt; to date the ETV case was a terminal procedure.

**Results:** Lateral ventricle volumes progressed from 1291 + 188 mm^3^ SEM pre-kaolin to 2455 + 1067, 2821 + 1139, 2280 + 1836, and 3538 + 2043 at post-kaolin days 1–5, 8–15, 22–29, and 42–69, respectively. Ventriculomegaly continued to progress post-shunt (mean 4051 mm^3^). ETV was performed successfully using standard neurosurgical procedures; i.e. frontal approach, visualization of the foramen of Monro (FoM), and opening the floor of the 3rd ventricle with balloon expansion. The piglet survived the procedure and after brain fixation via cardiac perfusion the path through the FoM and the ETV could be confirmed grossly with minimal damage to adjacent tissue.

**Conclusions:** The juvenile piglet represents a good, clinically-relevant large animal model of hydrocephalus that is amenable to ventricular shunting and ETV. Cauterization of the choroid plexus also seems feasible.

## O71 The effect of CSF flow blockage on the surgical management and outcomes of Chiari malformation

### Saniya Mediratta^1^, Linda D’Antona^1^, Claudia L Craven^1^, Lewis T Thorne^1^, Laurence D Watkins^1^, Ahmed K Toma^1^

#### ^1^National Hospital for Neurology and Neurosurgery, Queen Square, London, United Kingdom

##### **Correspondence:** Saniya Mediratta

*Fluids and Barriers of the CNS* 2018, **15(Suppl 2):**O70

**Introduction:** Chiari malformation can present with impairment of cerebrospinal fluid (CSF) flow across the cranial–vertebral junction (CVJ). This study aims to investigate the validity of MRI CSF flow studies in guiding management choices for patients with Chiari malformation.

**Methods:** A single-centre retrospective study included 68 patients (53 female, 15 male; ages 15–76, mean 37.5) with confirmed diagnosis of Chiari malformation and at least one MRI CSF flow study. Anterior and posterior flow at the CVJ was assessed using MRI CSF Flow studies. Clinical letters and radiological scans were analysed to determine the long-term clinical and radiological outcomes at a minimum of 6 months follow-up.

**Results:** 77 CSF Flow studies were analysed; 41 showed the presence of syrinx, 36 had no syrinx. 43 studies demonstrated reduced CSF flow at the CVJ, and 34 had normal flow.

Of the studies with syrinx and normal flow (n = 16), all patients who had no intervention had an unchanged or improved clinical outcome. 40% of patients who had foramen magnum decompression reported a deterioration in symptoms. For studies with syrinx and abnormal flow (n = 23), clinical outcomes were similar after no surgery or foramen magnum decompression (36% and 33% showed clinical improvement respectively).

**Conclusions:** Patients with normal CSF flow at the CVJ and syrinx did not clinically deteriorate if no surgery was performed, however, patients did experience worsening of symptoms after foramen magnum decompression. This study suggests that CSF MRI flow studies may be a useful assessment tool before deciding to operate in patients with Chiari Malformation.

## O72 The instrumented timed up and go test (TUGT): a new approach to the idiopathic normal pressure hydrocephalus (INPH) pre-surgical evaluation

### David Milletti^1^, Silvia Muscari^1^, Alberto Ferrari^6^, Giorgio Palandri^2^, Paolo Mantovani^2^, Stefania Magnoni^4^, Giulia Giannini^4^, Sabina Cevoli^4^, Federico Oppi^4^, Luca Albini Riccioli^5^, Tiziana Urli^3^, Vito A. Piserchia^3^, Lorenzo Chiari^6^, Pietro Cortelli^4^

#### ^1^Rehabilitation Medicine, IRCCS Istituto Scienze Neurologiche Bologna, Italy; ^2^Neurosurgery, IRCCS Istituto Scienze Neurologiche Bologna, Italy; ^3^Anesthesia and Intensive Care, IRCCS Istituto Scienze Neurologiche Bologna, Italy; ^4^Neurology, IRCCS Istituto Scienze Neurologiche Bologna, Italy; ^5^Neuroradiology, IRCCS Istituto Scienze Neurologiche Bologna, Italy; ^6^CIRI SDV University of Bologna, Italy

##### **Correspondence:** David Milletti

*Fluids and Barriers of the CNS* 2018, **15(Suppl 2):**O71

**Introduction:** iNPH’s gait apraxia can often be associated with vascular encephalopathy (VE). The use of TUGT to evaluate gait’s modification after cerebrospinal fluid tap test (CSFtt) is controversial. We instrumentally analyzed changes of Spatial–Temporal (ST) parameters during TUGT and compared data between pure iNPH and iNPH associated with VE.

**Methods:** Using MRI, 64 iNPH patients were divided into two groups: 29 without (A) and 35 with (B) VE. Groups did not differ in sex and age. Patients performed an instrumented TUGT, by means of three wearable inertial sensors. A smartphone app was used to collect data. 3 repetitions per test were recorded before and after (24–72 h) CSFtt. Comparisons were made with the Wilcoxon signed rank test. Test–retest reliability was assessed by ICC 1.1

**Results:** Test–retest reliability was excellent (ICC > 0.89). Stride length and cadence increased only in A: median values, respectively, from 73 to 77 (24 h) and 79 (72 h) cm (p < 0.001), and from 47 to 51 (24 h) and 52 (72 h) strides/min (p < 0.01). In A, speed progressively increased up to 72 h: from 55 to 59 (24 h) and 67 (72 h) cm/s (p < 0.001), whereas in B there was no significant improvement beyond 24 h. TUGT’s total time decreased at 72 in both groups (p < 0.001), but was constantly lower in A.

**Conclusions: I**nstrumented TUGT is useful to detect possible improvements of ST parameters after CSFtt. In the presence of VE, gait apraxia modifiability after the tap test is less evident than in pure iNPH patients.

## O73 Consideration of early shunt requirement after ETV

### Tomoru Miwa^1^, Takayuki Ohira^1^, Takashi Horiguchi^1^, Masahiro Toda^1^, Kazunari Yoshida^1^

#### ^1^Department of Neurosurgery, Keio University School of Medicine, Tokyo, Japan

##### **Correspondence:** Tomoru Miwa

*Fluids and Barriers of the CNS* 2018, **15(Suppl 2):**O72

**Introduction:** Endoscopic third ventriculostomy (ETV) is effective for non-communicating hydrocephalus, but in some cases, it is necessary to shunt early after ETV. We investigated the factors and pathology.

**Methods:** In 52 consecutive cases of initial ETV since 2000, four cases of non-communicating hydrocephalus were investigated requiring shunt within 1 month after ETV. Their average age was 34.0 years. In three cases, a contrast medium was injected into the ventricle intraoperatively, and the effectiveness of ETV and the postoperative cerebrospinal fluid absorption ability were evaluated.

**Results:** The primary disease was a brain stem tumor, spinal cord tumor, vertebral aneurysm after open surgery, and acoustic tumor after gamma knife. The average period from ETV to shunt was 18.8 (1–29) days. In the images, all cases showed obstruction of the outlet of fourth ventricle. Two cases of contrast injection were wash out within 24 h, but in one case, stagnation of the contrast agent was observed after 16 h, and ventricular dilatation was exacerbated, and the shunt was immediately performed. No findings that suspected early stoma occlusion after ETV. After the shunt, all cases progressed well.

**Conclusions:** The early shunt requirement cases after ETV is associated with absorption disorder in addition to adhesion of the posterior cranial fossa due to inflammation, local infection, bleeding component. It can be said that so-called communicating and non-communicating hydrocephalus were mixed clinically. Furthermore, subcutaneous fluid retained in the occipital region after surgery induce subclinical infection/inflammation, which may be the cause of ETV early failure.

## O74 Neuropsychological tests can predict the comorbidities of Alzheimer disease and parkinsonian syndromes in normal pressure hydrocephalus

### Chizuru Kamohara^1^, Madoka Nakajima^1^, Chihiro Akiba^1^, Kanei Jo^1^, Ikuko Ogino^1^, Masakazu Miyajima^2^

#### ^1^Department of Neurosurgery, Juntendo University, Hongo Bunkyo-ku, Tokyo, Japan; ^2^Department of Neurosurgery, Juntendo Tokyo Koto Geriatric Medical Center, Shinsuna Koto-ku, Tokyo, Japan

##### **Correspondence:** Masakazu Miyajima

*Fluids and Barriers of the CNS* 2018, **15(Suppl 2):**O73

**Introduction:** Comorbidities such as Alzheimer disease (AD) and Parkinsonian syndromes (PS) have a great influence on the prognosis of idiopathic normal pressure hydrocephalus (iNPH). The aim of this study was to differentially diagnose these comorbid diseases using neuropsychological tests.

**Methods:** Forty consecutive clinically suspected iNPH patients were enrolled. In all cases, DAT scan and cerebrospinal fluid (CSF) biomarkers (Aβ42, p-Tau) were measured. Rey Auditory Verbal Learning Trst (RAVLT), grooved pegboard test, and the Stroop test used in the European multicenter study were conducted at baseline and 1 year after surgery. Each test’s results were converted into scores from 0 to 100 and a radar chart was created from the scores.

**Results:** In 13 of 40 cases, the SBR on DAT scan was 3 or less, and PS was suspected to coexist (iNPH + PS). Associated AD was suspected in 15 cases with pTau of 30 pg/mL or more (iNPH + AD). Twelve cases were iNPH alone. With regard to iNPH + PS, Stroop test scores were significantly lower, which reflects executive function failure and decreased psychomotor speed. With regard to iNPH + AD, RAVLT scores were low, which reflects impairment of memory. After CSF shunt was performed, iNPH + PS patients showed significant improvement in RAVLT, and iNPH + AD patients showed significant improvements in all scores after CSF shunt. In patients with iNPH alone, the grooved pegboard test was the most sensitive to improvement after CSF shunt.

**Conclusions:** In those with iNPH + AD, memory impairment is most prominent, and in iNPH + PS, psychomotor speed is remarkably decreased.

## O75 Fall related chronic subdural hematoma on patients with idiopathic normal pressure hydrocephalus is high in 2 years after shunt surgery

### Hisayuki Murai^1^, Toshimasa Shin^1^, Shiro Ikegami^1^, Masayoshi Kobayashi^1^

#### ^1^Department of Neurosurgery, Chiba University Graduate School of Medicine, Chiba, Japan

##### **Correspondence:** Hisayuki Murai

*Fluids and Barriers of the CNS* 2018, **15(Suppl 2):**O74

**Introduction:** Disproportionately enlarged subarachnoid-space hydrocephalus (DESH) findings have been reported as a good predictor of favorable outcomes in shunt operation for patients with idiopathic normal pressure hydrocephalus (iNPH). However, patients with DESH contain both shunt responders and shunt non-responders. The aim of this study was to determine the difference in DESH MRI findings between shunt responders and shunt non-responders.

**Methods:** We evaluated DESH MRI findings and clinical features in 60 DESH cases of study of idiopathic normal pressure hydrocephalus on neurological improvement (SINPHONI) 2, who underwent LP shunting. We assessed correlations between the DESH finding measurements and change of 12 scales (i.e., mRS, iNPHGS total, iNPHGS gait, iNPHGS cognitive, iNPHGS urinary, TUG, 3-m reciprocating walking test [WT], MMSE, TAB, TMT-A, WAIS III, ZBI total) at 12 months after surgery. To determine the change of DESH findings, we measured Evans index, Callosal angle, z-Evans index, brain ventricle ratios at the anterior commissure (BVRs at AC), brain ventricle ratios at the posterior commissure (BVRs at PC).

**Results:** There was a positive correlation between a scale for gait ability and BVRs at PC. Furthermore, improved rate of TMT-A and a scale for frontal lobe abilities were negatively correlated with callosal angle and positively correlated with z-Evans index.

**Conclusion:** DESH MRI findings had a tendency to express clinical improvement of shunt responders. These findings may be a clue of understanding iNPH clinical statement and pathophysiology.

## O76 Fall related chronic subdural hematoma on patients with idiopathic normal pressure hydrocephalus is high in 2 years after shunt surgery

### Hisayuki Murai^1,2^, Toshimasa Shin^1,2^, Shiro Ikegami^2^, Masayoshi Kobayashi^2^

#### ^1^Department of Neurosurgery, Saiseikai Narashino Hospital, Narashino Japan; ^2^Department of Neurosurgery, Chiba University Graduate School of Medicine, Chiba, Japan

##### **Correspondence:** Hisayuki Murai

*Fluids and Barriers of the CNS* 2018, **15(Suppl 2):**O75

**Introduction:** Idiopathic normal pressure hydrocephalus (iNPH) is a disease of the elderly, who are at high risk of chronic subdural hematoma (SDH). Shunt surgery is one of the risk factors of chronic SDH. We studied the effect of fall on chronic SDH after shunt surgery.

**Methods:** From July 2002 to May 2015, 171 cases of iNPH were diagnosed and received shunt surgery. Incidence of chronic SDH with or without hematoma surgery was examined and compared with national database. The incidence of fall was analyzed in chronic SDH cases.

**Results:** A median observation period was 3 years and 10 months. Chronic SDH was observed in 27 patients (15.8%, 3.7% per year), with non-surgical treatments the surgery was needed in only 8 patients (4.7%, 1.1% per year). Eighteen chronic SDH cases (67%) were observed within first 2 years and 10 cases of them were associated with fall. Incidence of SDH surgery in our study seemed to be lower than that of previous reports (Birkeland et al. 2015). However, it was still 14 times higher than nationwide prevalence of chronic SDH in Japanese elderly (Toi et al. 2017).

**Conclusions:** Incidence of postoperative chronic SDH was high in first 2 years. The effect of fall was seemed to be also high in first 2 years. CT follow-up was useful for finding asymptomatic chronic subdural hematoma to start non-surgical treatments. Taking care to avoid fall is also important to prevent chronic SDH after shunt surgeries.

## O77 Novel CSF biomarkers for diagnosing spontaneous intracranial hypotension: combination of lipocalin-type prostaglandin D synthase and brain-type transferrin

### Yuta Murakami^1^, Koichi Takahashi^2^, Kyoka Hoshi^3^, Hiromi Ito^3^, Kiyoshi Saito^1^, Masakazu Miyajima^4^, Hajime Arai^4^, Tatsuo Mima^2^, Yasuhiro Hashimoto^3^

#### ^1^Department of Neurosurgery, Fukushima Medical University, Japan; ^2^Department of Neurosurgery, Sanno Hospital, Minato-ku, Tokyo, Japan; ^3^Department of Biochemistry, Fukushima Medical University, Japan; ^4^Department of Neurosurgery, Juntendo University, Bunkyo-ku, Tokyo, Japan

##### **Correspondence:** Yuta Murakami

*Fluids and Barriers of the CNS* 2018, **15(Suppl 2):**O76

**Introduction:** Spontaneous intracranial hypotension (SIH) is caused by cerebrospinal fluid (CSF) leakage. Definitive diagnosis can be difficult by clinical examinations and imaging studies. We investigated for CSF biomarkers to aid the diagnosis of SIH.

**Methods:** SIH was diagnosed with the following criteria: (i) evidence of CSF leakage by cranial MRI findings of intracranial hypotension and/or low CSF opening pressure; (ii) no recent history of dural puncture. We quantified CSF proteins such as albumin, immunoglobulin G (IgG), serum-type transferrin (Tf), lipocalin-type prostaglandin D synthase (L-PGDS), soluble amyloid precursor protein (sAPP) and brain-type Tf by ELISA or Western blotting. We also examined correlations between radioisotope (RI) residual activity rate or CSF opening pressure and the quantification data of six proteins.

**Results:** Comparing with non-SIH patients, SIH patients showed significant increase of brain-derived CSF glycoproteins such as L-PGDS, sAPP and brain-type Tf. Brain-type Tf was demonstrated to be produced in choroid plexus. Serum-derived proteins such as albumin, IgG, and serum-type Tf were also increased. A combination of L-PGDS and brain-type Tf differentiated SIH from non-SIH with sensitivity 94.7% and specificity 72.6%. Significant correlation were observed between clinical tests and L-PGDS or brain-type Tf. Decrease of RI residual activity rate was inversely correlated with increases of L-PGDS and brain-type Tf. Decrease of intracranial pressure was also inversely correlated with increases of L-PGDS and brain-type Tf. No other proteins were correlated with intracranial pressure or RI residual activity rate.

**Conclusions:** L-PGDS and brain-type Tf can be biomarkers for diagnosing SIH.

## O78 Pressure-controlled drainage: short-term ICP monitoring applications at the neurosurgical bedside

### Eva Nabbanja^1^, Afroditi-Despina Lalou^2^, Marek Czosnyka^2^, Laurent Gergele^2,3^, Matthew Garnett^1^, Zofia Czosnyka^2^

#### ^1^Division of Neurosurgery, Department of Clinical Neurosciences, Cambridge University Hospital, United Kingdom; ^2^Academic Neurosurgery, Division of Neurosurgery, Department of Clinical Neurosciences, University of Cambridge, United Kingdom; ^3^Department of intensive care, Ramsay Générale de Santé, Hôpital privé de la Loire, Saint Etienne, France

##### **Correspondence:** Eva Nabbanja

*Fluids and Barriers of the CNS* 2018, **15(Suppl 2):**O77

**Introduction:** CSF drainage in every-day neurosurgical care is very common, both in an emergency and a routine setting. Access to the CSF is gained via LP, shunt or reservoir, either for diagnostic or therapeutic reasons. Drainage is often arbitrarily determined by volume. However, the effects of excessive drainage are widely known and can be insignificant, or cause severe discomfort to the patient. Particularly in shunted patients, uncontrolled CSF drainage from the ventricular space can have long-term effects. We propose a short protocol for pressure-control CSF withdrawal in neurosurgical patients, using practical CSF dynamics and ICP monitoring.

**Materials and methods:** A reliable baseline ICP can be obtained via connection with fluid-filled manometer lines to a pressure amplifier connected to a computer, with a software to record the ICP and calculate parameters such as the compensatory reserve. ICP is monitored for a few minutes (10–20), then drainage is started, rechecking every 5–10 mls in order to avoid overdrainage. When the compensatory reserve is depleted, smaller changes in volume provoke steeper drops in ICP which can be avoided.

**Results:** Such a setting gives the advantage of a reliable baseline reading of the pressure and avoiding overdrainage by (1) checking the pressure (2) possessing knowledge of the compensatory reserves, which can warn about abrupt drops in ICP. Readings can be correlated and combined with clinical symptoms and clinical needs (diagnostic and therapeutic).

**Conclusions:** Short-term ICP monitoring using an appropriate software can be a useful tool to support clinical practice in certain patients. ICP manometry can be unreliable and overdrainage can be prevented in many cases.

## O79 Early stage p-tau measurement of cerebrospinal fluid can predict cognitive function 3 years after shunt surgery in patients with idiopathic normal pressure hydrocephalus

### Madoka Nakajima, Masakazu Miyajima, Ikuko Ogino, Chihiro Akiba, Kaito Kawamura, Hajime Arai

#### Department of Neurosurgery, Juntendo University, Tokyo, Japan

##### **Correspondence:** Madoka Nakajima

*Fluids and Barriers of the CNS* 2018, **15(Suppl 2):**O78

**Introduction:** Measurements of preoperative cerebrospinal fluid (CSF) biomarkers using the xMAP^®^ platform were able to predict the long-term functional prognosis in iNPH patients after shunt treatment for the first time.

**Methods:** Preoperative CSF biomarkers were measured in iNPH patients (n = 42), creating two groups based on a p-tau cut off value of 30 pg/mL Patents’ clinical progress was recorded for 3 years following lumboperitoneal shunt treatment. The relationships among functional prognosis and the modified Rankin Scale (mRS), Mini Mental State Examination (MMSE), Frontal Assessment Battery (FAB), and the iNPH Grading Scale (iNPHGS) were analyzed and compared between an age-adjusted low (n = 24, aged 75.7 ± SD 5.3 years) and a high p-tau groups (n = 11, aged 76 ± 5.6 years). Informed consent to publish has been obtained from the patients.

**Results:** The use of a p-tau cut-off value indicated that although it did not correlate with the preoperative MMSE score, it exhibited a significant negative correlation with the MMSE score at 1-year postoperatively (p = 0.02). This correlation strengthened with the extension of the postoperative period (2 years, p = 0.01, 3 years, p < 0.001, r^2^ = 0.352). Cognitive function improved in the early postoperative period in the low p-tau group and was maintained thereafter. In contrast the high p-tau group, after early postoperative (3–6 months) improvement, gradual decline to baseline levels by the 2nd and 3rd postoperative year occurred (p < 0.001). Furthermore, both p-tau groups had initial mRS improvement, although the high p-tau group had some decline 3rd years after surgery.

**Conclusions:** High p-tau is indicative of concomitant Alzheimer’s pathology, and therapeutic effects of iNPH shunt treatment with coexisting Alzheimer’s disease were limited to a shorter time period.

## O80 A new method for diagnosis of normal pressure hydrocephalus: callosal arch ratio

### Omer Ozdemir, Orhan Barlas

#### Department of Neurosurgery, Istanbul University Faculty of Medicine, Istanbul, Turkey

##### **Correspondence:** Omer Ozdemir

*Fluids and Barriers of the CNS* 2018, **15(Suppl 2):**O79

**Introduction:** The purpose of this study was to test the hypothesis that the ‘Callosal Arch Ratio’ (CAR), described as the ratio of the greatest distances of the corpus callosum and the cortex to the Anterior Commissure-Posterior Commissure Line (ACPCL) as measured at 90° to the ACPCL, correctly predicts shunt responsiveness of Normal Pressure Hydrocephalus (NPH).

**Methods:** Callosal Arch Ratio values were determined on preoperative magnetic resonance images (MRI) of 27 patients with presumed NPH undergoing VPS surgery between 2014 and 2016. Patients with an improvement of one point in the Normal Pressure Hydrocephalus Grading Scale (NPHGS) at 6 months postoperatively were considered shunt responders (Group 1) and those that did not show this improvement as non-responders (Group 2). The CAR results of these two groups were compared, as well as those of a control group (CG).

**Results:** There were 22 responders and 5 non-responders to shunt surgery. There was no difference between the two groups and the control group regarding demographical data (p > 0.05). The mean value of CAR was 0.66 (± 0.01) for Group 1 (responders to VPS), 0.60 (± 0.01) for Group 2 (non-responders to VPS) and 0.38 (± 0.01) for the control group. The difference between groups 1, 2 and CG was statistically significant (p = 0.000), and the difference between groups 1 and 2 was statistically significant (p = 0.001).

**Conclusion:** These preliminary results suggest that CAR may be a valuable prognostic tool in selecting patients for shunt surgery. Studies with a higher number of patients are needed to obtain more reliable results.

## O81 Identification of *ETINPH* gene

### Jun Zhang^1^, Akhil Padarti^1^, Sangjin Kim^2^, Madan Baral^2^, Michael A. Williams^3^, Daniele Rigamonti^4^, Abhay Moghekar^4^

#### ^1^Texas Tech University Health Science Center El Paso, El Paso, Texas, United States; ^2^University of Texas at El Paso, El Paso, Texas, United States; ^3^The Adult and Transitional Hydrocephalus Program at University of Washington Medical Center, El Paso, Texas, United States; ^4^The Adult Hydrocephalus Center at Johns Hopkins University School of Medicine, El Paso, Texas, United States

##### **Correspondence:** Jun Zhang

*Fluids and Barriers of the CNS* 2018, **15(Suppl 2):**O80

We previously reported a novel form of hydrocephalus termed essential tremor-idiopathic normal pressure hydrocephalus (ETINPH) identified in a large five-generation pedigree and mapped ETINPH locus to a 17-cM interval between 19q12-13.31 on chromosome 19 (chr19).

In this report, we employed Whole Exome Sequencing (WES) to sequence three ETINPH patients and three normals from this pedigree. WES was performed with approximately 60× coverage and 100 bp paired-end (PE) reads and mapped to human genome GRCh38 to generate genetic variant files: SNPs and INDELs. We found that unique SNPs and INDELs shared by patients on chr19 is much higher than that on other chromosomes. Compared with normals, SNPs shared by patients on chr19 is fivefold higher than on other chromosomes and INDELs shared by patients on chr19 is nearly eightfold higher than on other chromosomes. This strongly reverberates our linkage result. By analyzing WES data with exome data parsing and novel variants extraction, we narrowed down ETINPH gene to 25 genes on chr19. Narrowing our focus to the 17-cM critical interval on 19q12-13.31, the ETINPH gene has been further narrowed down to mutations on 8 genes. These 8 non-synonymous mutations are currently analyzed with functional prediction assays (SIFT, PolyPhen2, etc.). A second ETINPH pedigree has been identified and sample has been collected for WES (100× coverage), which will lead to ETINPH gene identification.

## O82 Unravelling the genetics of hydrocephalus

### Sangjin Kim^1^, Jon Mohl^1^, Daniele Rigamonti^2^, Abhay Moghekar^2^, Akhil Padarti^3^, Jun Zhang^3^

#### ^1^University of Texas at El Paso, El Paso, Texas, United States; ^2^The Adult Hydrocephalus Center at Johns Hopkins University School of Medicine, El Paso, Texas, United States; ^3^Texas Tech University Health Science Center El Paso, El Paso, Texas, United States

##### **Correspondence:** Jun Zhang

*Fluids and Barriers of the CNS* 2018, **15(Suppl 2):**O81

Idiopathic normal pressure hydrocephalus (iNPH) is a type of hydrocephalus affecting the elderly, characterized by unexplained symmetric gait disturbance, dementia, and/or urinary incontinence without elevated CSF pressure and other causative disorders. It has been hypothesized that genetic factors play an important role in the pathogenesis of iNPH. However, iNPH is a complex and multifactorial disorder which frequently overlaps with other neurological disorders, such as Alzheimer’s and Parkinson’s Diseases (AD/PD), therefore the genetics and molecular pathogenesis of human iNPH remain unknown.

In this pilot study, we hypothesize that there are some common genetic variants among iNPH patients. To identify these novel biomarkers related to iNPH, using Illumina Human Omin1-Quad v.1 single nuclear polymorphism (SNP) microarrays which contain 450,000 SNPs spanning the whole genome, we performed the whole genome genotyping scan for 71 patients of iNPH recruited from our Hopkins clinics and 71 controls matched for sex, age, and race from the current NCBI database. Using a case control methodology such as logistic regression, random forest models, and other rigorous statistical methods to account for the multiple comparisons made, we are performing a pilot small-scale genome-wide association study to try to identify “relatively significant” SNPs or CNVs associated with the iNPH. At the same time, we will try to build a statistical model with these markers that can be used for prognosis and diagnosis of iNPH. Finally, we will perform pathway analysis by utilizing all identified genetic variants corresponding to this pilot biomarker search to extract more biological information related to iNPH.

## O83 Hydrocephalus etiology: a unified theory or multi-directional approach?

### Jogi V. Pattisapu

#### School of Medicine, University of Central Florida, Orlando, Florida, United States

##### **Correspondence:** Jogi V. Pattisapu

*Fluids and Barriers of the CNS* 2018, **15(Suppl 2):**O82

**Introduction:** Hydrocephalus is not excessive CSF accumulation under pressure. Although this condition has been recognized for several centuries, we still do not have a comprehensive understanding of the complex pathophysiology involved. The condition can occur in the fetus and is now more frequently recognized in older individuals. This continuum and the multiple “faces” of hydrocephalus make it a very interesting and almost indefinable situation.

Over the past several years, recent advances in neuroradiologic techniques, genetic and molecular biology of hydrocephalus provide the opportunity to revisit the initial hypotheses. Excessive CSF accumulation within the CNS may be the result of a complex mechanism gone awry, or the primary causative pathology in an otherwise well-balanced system. The numerous causes of hydrocephalus, and the varied manifestations of this condition beg the need for a more unified theory.

**Methods:** Causative factors of hydrocephalus can be considered in many different ways: (1) restriction of blood flow, (2) blockage of normal CSF flow, (3) irregularities in normal brain pulsations, (4) loss of vascular integrity, (5) volume transmission abnormalities, (6) asynchrony of arterial/venous pressure waves, (7) extracellular matrix disfiguration (7) abnormal CSF absorption, (8) inadequate toxin removal, (9) “poisoning”, (10) abnormal “brain turgor”, (11) excessive reactionary responses, (12) inadequate activation of reparative mechanisms, and (13) abnormal expression of growth factors/cytokines.

**Results:** Given these various theoretical approaches, it seems natural to attempt a unified approach to the “cause” of hydrocephalus. Such an approach is absolutely crucial in hopes of identifying therapeutic targets at various stages of this complex condition. Conversely, a multi-directional attack also seems appropriate, if a logical progression can be established, or an appropriate sequence of events can be ascertained. However, given the various ages and etiologies involved, there may be a simple, yet unrecognized basic pathophysiological mechanism that integrates our understanding.

In this presentation, we will review the current philosophies of thought and attempt to unify them into similar avenues for discussion. These plausible and recognized theories may serve as a model of “pattern” recognition, and may have relevance to the currently available treatment options. This attempt will assist in our understanding, and allow reconsideration of accepted pathophysiological models of hydrocephalus. It will also allow us to consider several plausible and simultaneously applicable avenues of research. The basic question ‘why CSF?’ is still not clearly defined in our thinking!

**Conclusion:** We hope to stimulate and provoke discussion regarding the numerous schools of thought regarding these basic, simple and yet elusive concepts regarding the etiology of hydrocephalus.

## O84 Secondary non-responding versus shunt insufficiency in patients with idiopathic normal pressure hydrocephalus after shunt surgery

### Ullrich Meier, Pawel Gutowski, Sergej Rot, Johannes Lemcke

#### Department of Neurosurgery, Unfallkrankenhaus Berlin, Germany

##### **Correspondence:** Johannes Lemcke

*Fluids and Barriers of the CNS* 2018, **15(Suppl 2):**O83

**Objectives:** After ventriculo-peritoneal (vp) shunt surgery for idiopathic normal pressure hydrocephalus (iNPH) with programmable gravitational valves, a certain proportion of patients develop secondary clinical worsening after initial improvement of the clinical symptoms. The aim of this study is to analyze this group of secondary non-responders.

**Methods:** Three hundred and sixty one consecutive patients with idiopathic normal pressure hydrocephalus were included in our prospective shunt registry (mean age 74 years) and underwent surgery with implantation of vp shunts with programmable low-pressure valves plus non-programmable gravitational units (pressure level 50–70 mmH_2_O plus 200–300 mmH_2_O). All patients were routinely followed up 1, 2, 3, 4, 5, 6 and 7 years after shunt surgery.

**Results:** After surgery, 308 patients showed clinical improvement measured by the Kiefer score (86% primary responders). Out of these group, we observed secondary worsening of minimum two points (Kiefer Score) 2.7 years after shunt surgery in 53 patients (14%). In 33 of these patients, the low pressure-valve was re-programmed to pressure levels between 0 and 30 mmH_2_O. 20 of these patients underwent shunt fluoroscopy with contrast medium (shuntography). 13 patients underwent a replacement of the non-programmable gravitational unit by a programmable gravitational unit. 14 of these patients improved after taking action. 31 patients showed no improved after the procedures. We had a drop out rate of 8 patients.

**Conclusion:** Fourteen percent of patients with iNPH are at risk for secondary clinical worsening 3 after shunt surgery. Twenty six percent of these patients benefited for additional years from pressure level management and/or shunt valve revision.

**Keywords:** Idiopathic normal pressure hydrocephalus, Shunt registry, Responder, Non-responder, Shunt insufficiency

## O85 Repeated endoscopic third ventriculostomy: our case series

### Valentina Pennacchietti, Paola Ragazzi, Angela Coppola, Mario Cacciacarne, Pierpaolo Gaglini, Paola Peretta

#### Department of Pediatric Neurosurgery, Children Hospital Regina Margherita, Turin, Italy

##### **Correspondence:** Valentina Pennacchietti

*Fluids and Barriers of the CNS* 2018, **15(Suppl 2):**O84

**Introduction:** endoscopic third ventriculostomy (ETV) provides an effective treatment for triventricular hydrocephalus. Failure rates in children are 10–40% in different series. In case of closure of the stoma, the choice between a ventriculoperitoneal shunt (VPS) or another ETV can be challenging and no standardized protocol has been described yet.

**Materials and methods:** we reviewed 151 children treated with ETV from 2006 to 2017 in our Center. A repeated ETV was performed on 29 patients (mean age: 5.9 years). The primary etiologies of hydrocephalus were: 11 tumors, 7 intraventricular hemorrhages, 8 acqueductal stenosis, one lumbosacral myelomeningocele, one vein of Galen malformation and one Blake’s pouch cyst. The interval between the first and second ETV was 3 days–6 years (mean follow-up 64.5 months).

**Results:** In 22 patients we twice performed an ETV, while in 7 cases we attempted a third ETV. Among the 22 patients, in 5 cases it was necessary to implant a VPS. In the group of the third ETV, 2 patients required a VPS. Our ETV failure rate at first attempt was 19.3%, while failure after repeated ETV was 27.6%. In 51.7% the ETV failed within 6 months; in 37.9% the failure occurred within the first 3 months after surgery. A repetition of the ETV was successful in 72.4%.

**Conclusions:** According to our data and to literature a repeated ETV is a valid treatment after the failure of a previous ETV. As our experience suggests, even a third ETV might be effective, if the malresorptive nature of the hydrocephalus has been ruled out.

## O86 Cortical metabolic changes in normal pressure hydrocephalus after shunt: our experience

### Gianpaolo Petrella^1^, Agazio Menniti^1^, Alberto Delitala^1,2,3,4^ Oreste Bagni^2^, Luca Filippi^2,^ Agostino Chiaravallotti^3^

#### ^1^Department of Neurosurgery, S.Maria Goretti Hospital, Latina, Italy; ^2^Department of Nuclear Medicine, S.Maria Goretti Hospital, Latina, Italy; ^3^Department of Biomedicine and Prevention, Nuclear Medicine, University Tor Vergata, Rome, Italy; ^4^Department of Neurosurgery, S.Camillo Forlanini Hospital, Rome, Italy

##### **Correspondence:** Gianpaolo Petrella

*Fluids and Barriers of the CNS* 2018, **15(Suppl 2):**O85

**Introduction:** To investigate the cortical metabolic changes in patients affected by normal pressure hydrocephalus (NPH) after a placement of ventriculoperitoneal (VP) shunt.

**Materials and methods:** 10 patients with suspected NPH underwent a CSF hydrodynamics evaluation based on an lumbar infusion test. Intracranial Elastance (IE) was evaluated by measuring the slope of the linear regression between the diastolic ICP values and the corresponding amplitude of each CSF pulse pressure wave. Patients with a IE > 0.30 underwent a PET and a placement of A ventriculoperitoneal (VP) shunt. All patients improved after shunting.

Brain PET/CT with 18F FDG was performed in all the subjects at baseline and after one. A region of Interes (ROI) was automatically drawn on each lobe (frontal, parietal, temporal, occipital and limbic) by means of WFU Pickatlas implemented in statistical parametric mapping (SPM8, Wellcome Department of Cognitive Neurology, London, UK). Data were then normalized for the average counts of the cerebellum. A paired t test was then performed for the comparison of 18F FDG cortical uptake before (PET1) and after surgery (PET2).

**Results:** As compared to PET1, PET2 showed a significant increase in brain glucose consumption in all the cortical areas examined. I.e. as for left frontal lobe, cortical 18F FDG glucose consumption was 1.032 ± 0.12 in PET1 and 1.2 ± 0.09 in PET2 (p < 0.001).

**Conclusions:** The results of our study show that NPH may benefit of surgical treatment. 18F FDG PET could be considered a potential diagnostic imaging modality in these patients.

## O87 Usefulness of the lumbar infusion test as a screening tool for the prognosis of CSF shunting in normal pressure hydrocephalus (NPH): our experience

### Gianpaolo Petrella^1^, Agazio Menniti^1^, Alberto Delitala^1,6^, Manuela Galli^2,3^, Caterina Vicidomini^4^, Ana Rozin Kleiner^2^, Veronica Cimolin^2^, Maria Francesca de Pandis^5^

#### ^1^Department of Neurosurgery, S.Maria Goretti Hospital, Latina, Italy; ^2^Department of Electronics, Information and Bioengineering, Politecnico di Milano, Milan, Italy; ^3^IRCCS San Raffaele Pisana, Tosinvest Sanità, Roma, Italy; ^4^Biostructure and Bioimaging Institute (IBB), National Research Council (CNR), Naples, Italy; ^5^San Raffaele Cassino Hospital, Tosinvest Sanità, Cassino, Italy; ^6^Department of Neurosurgery, S.Camillo Forlanini Hospital, Rome, Italy

##### **Correspondence:** Gianpaolo Petrella

*Fluids and Barriers of the CNS* 2018, **15(Suppl 2):**O86

**Introduction:** Normal Pressure Hydrocephalus (NPH) is a neurological condition which is commonly confused with Parkinson’s or Alzheimer’s diseases as well as dementia. Cerebrospinal fluid (CSF) shunting was acclaimed to be a treatment of choice in NPH patients. In this work we evaluate the usefulness of lumbar infusion test (LIT) in the selection of NPH patients for a ventriculoperitoneal (VP) shunt implantation.

**Materials and methods:** We report our experience on 9 patients with NPH diagnosis that underwent also a diagnostic LIT. A different CSF dynamic parameters were determined. Only the patient with intracranial elastance (IE) ≥ 0.3 underwent to implant a VP shunt. Every patient has carried out two neuropsychological evaluations (MMSE, FAB, MOCA) and of 3D-gait analysis, pre- (30 days pre-surgery) and 3 months post-surgical intervention.

**Results:** Three months post-intervention the patients increased the FAB (t7 = − 3.870; p = 0.006) and MOCA (t7 = − 3.564; p = 0.009) scores. Moreover, patients presented improvements in the gait spatiotemporal parameters. In particular, they decreased the support phase and double support phase percentage, and increased the balance phase percentage. They increased the cadence, gait mean velocity and stride length also.

**Conclusions:** Although in our study there is no control group of patients treated with IE < 0.3, all patients in our group improve their cognitive and motor condition after the shunt implantation. On the basis of our previous experience, our data suggest that the evaluation of IE is very useful in predicting a successful prognosis of the derivative intervention in NPH patients.

## O88 Brain tamponade mechanical parameters and its treatment by extracorporeal brachiocephalic selective circulation

### Gianpaolo Petrella^1^, Carmello Anile^2^

#### ^1^Department of Neurosurgery, S.Maria Goretti Hospital, Latina, Italy; ^2^Department of Neurosurgery, Catholic University, Rome, Italy

##### **Correspondence:** Gianpaolo Petrella

*Fluids and Barriers of the CNS* 2018, **15(Suppl 2):**O87

**Introduction:** An unchecked increase of intracranial pressure (ICP) causes a brain tamponade (BT). It is generally accepted that BT occurs when ICP is the same as values of systemic blood pressure (SBP).

**Method:** Six sheeps were intubated, anesthetized and put in extracorporeal brachiocephalic circulation (EBC). Through a subdural needle, in which Ringer Lactate was infused, we produced BT.

We recorded: Electrocardiogram, SBP, Carotid arterial blood pressure (CABP), Cerebral blood flow (CBF), ICP.

In each animal we performed three different types of procedures.

1. We started to infuse Ringer Lactate while measuring all parameters until mean cerebral blood flow was zero.
2. We performed the same procedure in conditions of extracorporeal brachiocephalic selective circulation in continuous flow.
3. After putting the animals in extracorporeal brachiocephalic selective circulation with a pulsed flow and reaching a BT, we turned off the pump, thus obtaining a continuous flow.

**Results:** (1) In normal conditions, when the BT occurred the ICP was 70 mmHg and CABP was 110 mmHg with a CPP of 40 mmHg. (2) In EBC circulation in continuous flow, BT occurred when ICP reached a value almost equal to CABP, with a CPP of 15–16 mmHg. (3) After reaching a BT with pulsed flow, the pump was turned off and partial recovery of brain circulation was recorded.

**Conclusion:** These data exclude that a simple pressure gradient drop to 0 value or less could be the fundamental variable determining the arrest of intracranial blood flow circulation, which could instead be related to the pulsation of the arterial blood.

## O89 The importance of neck position in ICP control

### Gianpaolo Petrella^1^, Zofia Czosnyka^2^, Marek Czosnyka^2^

#### ^1^Department of Neurosurgery, S.Maria Goretti Hospital, Latina, Italy; ^2^Academic Neurosurgical Unit, Addenbrooke’s Hospital, Cambridge, United Kingdom

##### **Correspondence:** Gianpaolo Petrella

*Fluids and Barriers of the CNS* 2018, **15(Suppl 2):**O88

**Introduction:** The concept of Intracranial Pressure (ICP), function of the volume and compliance of each component of the intracranial compartment, was proposed by Alexander Monro (1733–1817) and his student George Kellie (1758–1829) during the late eighteenth century. ICP is normally 7–15 mmHg in adults who are supine. Raised ICP is a life threatening condition that is common to many neurological and non-neurological illnesses. Unless recognized and managed early it may cause secondary brain injury due to reduced cerebral perfusion pressure (CPP), and progress to brain herniation and death. Management of raised ICP includes care of airway, ventilation and oxygenation, adequate sedation and analgesia, neutral neck position, head elevation, short-term hyperventilation and hyperosmolar therapy.

**Method:** To determinate the role of the neck position in the management of ICP we study 10 patients in the Intensive Care Unit (ICU) with parenchymal or ventricular ICP monitoring for different neurosurgical pathologies. We evaluated the change in the median value of the Intracranial Pressure just putting the head in neutral position to promote venous drainage via the external jugular veins.

**Results:** Six patient were female, the median age was 50 years, the value of ICP was included between 6 and 16 mmHg.

Putting the head in right neutral position the abatement of median ICP was included between 4 and 8 mmHg, restoring a normal ICP.

**Conclusions:** Our experience shows how the importance of the position of the neck. It is an easy, quick, economic and safe maneuver which can reduce by itself, the level of Intracranial Pressure.

## O90 Malfunction or under-drainage? The value of the infusion test

### Gianpaolo Petrella^1^, Zofia Czosnyka^2^, Marek Czosnyka^2^, Carmelo Anile^3^

#### ^1^Department of Neurosurgery, S.Maria Goretti Hospital, Latina, Italy; ^2^Academic Neurosurgical Unit, Addenbrooke’s Hospital, Cambridge, United Kingdom; ^3^Department of Neurosurgery, Catholic University, Rome, Italy

##### **Correspondence:** Gianpaolo Petrella

*Fluids and Barriers of the CNS* 2018, **15(Suppl 2):**O89

**Introduction:** Patients who never clinically improve or improve and then worsen after CSF shunting constitute a difficult group to manage. The difficulty partially depends on establishing whether their clinical condition is due to shunt. We report our experience using infusion test with two different type of software (ICM+ Cambridge University, NEMO Catholic University) to estimate shunt patency.

**Method:** Normal saline or Ringer lactate was infused at a constant rate into the shunt prechamber or previously implanted Ommaya reservoir.

With ICM+ we analyzed retrospectively 312 tests, performed in 197 patients, to investigate the parameters describing CSF dynamics that correlate with the clinical finding of shunt malfunction.

With Nemo we analyzed 15 NPH patients with suspect of malfunction.

**Results:** In 161 of the 312 infusion tests results indicated under-draining shunts. Patients in the under-draining group had higher baseline (p = 5 * 10 − 9) and plateau CSF pressures (p = 0), higher resistance to CSF outflow (p = 0) and higher levels of baseline pulse amplitude waveform (p = 0.044) compared to patients with a properly performing shunt. During the test a significantly greater vasogenic waves (p = 5.8 * 10 − 7) and lower compensatory reserve (p = 0.0027) was noticed in patients with blocked shunts.

In 10 of the 15 patients we found a higher baseline and plateau CSF pressure comparing with the opening pressure of the valve.

All the patients with altered parameters underwent operative revision of the shunt with an improvement in symptoms.

**Conclusions:** Infusion test is easy, safe and clinically useful, aiding decision in difficult clinical situations, where shunt malfunction is suspected but not certain.

## O91 Can the difference between initial and final pressure on tap test to predict clinical response to shunt in normal pressure hydrocephalus patients?

### Fernando C. G. Pinto^1^, Gabriel A. S. Mendes^1^, Rodolfo C. Reis^1^, Matheus F. Oliveira^1^, Renata Gobbato^1^, Manoel J. Teixeira^2^

#### ^1^Cerebral Hydrodynamics Group, Division of Functional Neurosurgery, Institute of Psychiatry, Hospital das Clínicas, University of São Paulo, Brazil; ^2^Department of Neurosurgery, University of São Paulo, Brazil

##### **Correspondence:** Fernando C. G. Pinto

*Fluids and Barriers of the CNS* 2018, **15(Suppl 2):**O90

**Introduction:** Tap Test (TT) can predict the clinical response to shunt. The objective of this study was to determine a correlation between the initial and final pressure difference (50 mL CSF removal) with clinical outcome after 12 months of shunt surgery in NPH patients.

**Methods:** 42 NPH patients, who presented positive TT, were enrolled in the study. Of these, 14 were excluded because they did not have complete TT pressure data or they were not submitted to shunt. After surgery, the patients were followed for 12 months. Japanese NPH scale, BERG, DGI and TUG were compared and related to the difference between the initial and final TT pressure.

**Results:** 28 NPH patients were included in the study. The mean values of the initial and final TT pressure and the difference between them were 17.3, 4.8 and 12.5 cmH_2_O. The preoperative and post-12 months of shunt surgery scores on the Japanese NPH scale, BERG, DGI and TUG were, respectively, 5.6 and 3.3, 33.2 and 41.6, 8.7 and 14.0, 38.2 and 43.8 s. Eighteen patients improved the TUG and the Japanese NPH scale, 8 worsened. In the statistical analysis, the t-test showed a difference between preoperative and after 12 months of surgery scores, but the Pearson correlation coefficient did not show a correlation between the difference of TT initial and final pressures and the clinical outcome.

**Conclusion:** TT showed a positive predictive value of 64.3%, but the initial and final pressure difference did not present a relationship with the clinical outcome after shunt.

## O92 Preliminary results of safety and clinical performance of the Sphera Pro™ programmable valve

### Fernando C. G. Pinto^1^, Rodolfo C. Reis^1^, Nikolas Harada^2^, Flávia M. G. Pinto^1^, Camila S. Cechi^1^, Renata Gobbato^1^, Salamandra S. S. Silvestre^1^, Regina H Maykot^1^, Gabriel A. S. Mendes^1^, Juliana Tornai^1^, João V. R. Morais^3^, Manoel J. Teixeira^4^

#### ^1^Cerebral Hydrodynamics Group, Division of Functional Neurosurgery, Institute of Psychiatry, Hospital das Clínicas, University of São Paulo, Brazil; ^2^HpBio, São Paulo, Brazil; ^3^Instituto Federal de Educação, Tecnologia e Ciência de São Paulo, Brazil; ^4^Department of Neurosurgery, University of São Paulo, Brazil

##### **Correspondence:** Fernando C. G. Pinto

*Fluids and Barriers of the CNS* 2018, **15(Suppl 2):**O91

**Introduction:** Programmable valve is the first choice for the treatment of NPH patients and it is also indicated for arachnoid cyst (AC) and idiopathic intracranial hypertension (IIH). The aim of this study was to confirm the safety and clinical performance of the Sphera Pro™ programmable valve.

**Methods:** 9 NPH, 1 temporal AC and 1 IIH patients were included sequentially in the study from July 2017 to February 2018. After shunt surgery, the postoperative evaluations took place at 10 days, 3 months, 6 months and 1 year. Primary outcomes: frequency and severity of complications or side effects. Secondary outcomes: clinical improvement after shunt.

**Results:** Seven patients had idiopathic NPH, 2 secondary NPH and the mean age was 73.6. In all of them the valve was initially set at 3 cmH_2_O with antigravitacional device of 15 cmH_2_O. Two NPH patients required 1 adjustment for pressure reduction to 1 cmH_2_O. All patients improved on the Japanese NPH scale (mean pre = 5, mean post 3 months = 2). There was no overdrainage, spontaneous or after 3 T MRI valve deprogramming or infections. In the AC patient, the valve was set at 3 cmH_2_O and symptoms improved. In the IIH patient, the valve was initially set at 10 cmH_2_O and partial improvement of the symptoms were observed after 3 adjustments down to 1 cmH_2_O.

**Conclusion:** Despite the small sample and the short follow-up period, preliminary data from this pilot study show that Sphera Pro™ programmable valve is safe when used in NPH, IHH or arachnoid cyst adult patients.

## O93 Noninvasive monitoring of intracranial pressure

### Fernando C. G. Pinto^1^, Gabriel A. S. Mendes^1^, Rodolfo C. Reis^1^, Flávia M. G. Pinto^1^, Gustavo Frigieri^2^, Cintya Y. Hayashi^3^, Daniel M. Costa^2^, Sergio Mascarenhas^2^, João V. R. Morais^4^, Manoel J. Teixeira^5^

#### ^1^Cerebral Hydrodynamics Group, Division of Functional Neurosurgery, Institute of Psychiatry, Hospital das Clínicas, University of São Paulo, Brazil; ^2^Braincare Health Tecnology, São Carlos, Brazil; ^3^School of Medicine, Department of Neurology, University of São Paulo, Brazil; ^4^Instituto Federal de Educação, Tecnologia e Ciência de São Paulo, Brazil; ^5^Department of Neurosurgery, University of São Paulo, Brazil

##### **Correspondence:** Fernando CG Pinto

*Fluids and Barriers of the CNS* 2018, **15(Suppl 2):**O92

**Introduction:** Intracranial pressure monitoring is an important factor in the definition of the treatment of cerebral hydrodynamic disorders. In this pilot study, the objective was to evaluate the performance of noninvasive intracranial pressure monitoring (niICP) in adult patients with idiopathic intracranial hypertension (IIH), arachnoid cyst or hydrocephalus with inadequately functioning shunt.

**Methods:** During routine outpatient care, 10 patients with cerebral hydrodynamic disorder (6 IIH, 3 arachnoid cyst, 1 hydrocephalus with inadequately functioning shunt) underwent niICP, which detects the nanoscale variation of the skull for 5 min in the positions: dorsal decubitus, sitting, standing and valsalva maneuver.

**Results:** Seven of the ten patients had focal neurological symptoms or clinical signs of intracranial hypertension. In these patients there was abnormal ICP curves and positive correlation with the data obtained in niICP. Five patients with IIH presented curves with P2 > P1, one patient with frontal arachnoid cyst had P1 = P2 and one patient with hydrocephalus with inadequately functioning shunt had horizontal P3. These patients received a shunt implant indication for the treatment of cerebral hydrodynamic disorders.

**Conclusion:** Noninvasive monitoring of intracranial pressure is a practical, fast and efficient tool for the evaluation of patients with hydrocephalus, HII and arachnoid cyst.

## O94 “Four scores” comorbidity evaluation and postoperative complications in iNPH

### Tiziana Urli^1^, Giorgio Palandri^2^, Paolo Mantovani^2^, Vito A. Piserchia^1^, Giulia Giannini^3^, Stefania Magnoni^3^, Sabina Cevoli^4^, Federico Oppi^4^ David Milletti^5^, Luca Albini Riccioli^6^, Carmelo Sturiale^2^, Pietro Cortelli^3^

#### ^1^Anesthesia and Intensive Care, IRCCS Istituto Scienze Neurologiche Bologna, Italy; ^2^Neurosurgery, IRCCS Istituto Scienze Neurologiche Bologna, Italy; ^3^DIBINEM, University of Bologna, Italy; ^4^Neurology, IRCCS Istituto Scienze Neurologiche Bologna, Italy; ^5^Rehabilitation Medicine, IRCCS Istituto Scienze Neurologiche Bologna, Italy; ^6^Neuroradiology, IRCCS Istituto Scienze Neurologiche Bologna, Italy

##### **Correspondence**: Tiziana Urli

*Fluids and Barriers of the CNS* 2018, **15(Suppl 2):**O93

**Introduction:** The ISHCSF task force (2013) on comorbidities in idiopathic normal pressure hydrocephalus (iNPH) underlined the need of continuing studies on both specific and universal scores; the aim of this study was to describe the epidemiology of comorbidities and their relationship with postoperative complication using four scores: the Comorbidity Index (CMI) proposed by Kiefer in 2006; the Cumulative Illness Rating Scale Geriatrics (CIRS-G) and the Charlson Comorbidity Index (CCI), already validated in the elderly; the American Society of Anesthesiologists (ASA) classification as part of the preoperative evaluation before shunt surgery.

**Methods:** From 2015 to 2018 a multidisciplinary team selected 38 patients for shunt surgery after diagnosis of “probable iNPH” and exclusion of very severe comorbidities. Baseline demographic, cognitive, functional, and pathological characteristics were recorded and converted into the four scores. A ventriculoperitoneal shunt was positioned in all patients. Postoperative complications were registered during a 2 months period.

**Results:** Each score pointed out different aspects of concomitant diseases. All patients had at least two CIRS-G “level 2” items. The scores median values (ranges) were: CIRS-G 9 (4–16), Charlson 1 (0–3), Kiefer 3 (0–6), ASA 3 (2–3). Symptomatic and asymptomatic postoperative complications were registered in 10 patients (subdural hygroma/hematoma, seizures, shunt dysfunction, respiratory failure) and the statistical correlation with comorbidity scores, calculated at different cut-off values, was significant (p < 0.05) for CIRS-G > 9 and Charlson > 1.

**Conclusions:** Concomitant diseases were always present in this study population: each score described them in a different way. CIRS-G > 9 and Charlson > 1 were associated with an increased risk of postoperative complications.

## O95 “Four scores” comorbidity evaluation and outcome prediction in iNPH

### Tiziana Urli^1^, Vito A. Piserchia^1^, Giorgio Palandri^2^, Paolo Mantovani^2^, Giulia Giannini^3^, Stefania Magnoni^3^, Sabina Cevoli^4^, Federico Oppi^4^ David Milletti^5^, Luca Albini Riccioli^6^, Carmelo Sturiale^2^, Pietro Cortelli^3^

#### ^1^Anesthesia and Intensive Care, IRCCS Istituto Scienze Neurologiche Bologna, Italy; ^2^Neurosurgery, IRCCS Istituto Scienze Neurologiche Bologna, Italy; ^3^DIBINEM, Università di Bologna, Italy; ^4^Neurology, IRCCS Istituto Scienze Neurologiche Bologna, Italy; ^5^Rehabilitation Medicine, IRCCS Istituto Scienze Neurologiche Bologna, Italy; ^6^Neuroradiology, IRCCS Istituto Scienze Neurologiche Bologna, Italy

##### **Correspondence:** Tiziana Urli

*Fluids and Barriers of the CNS* 2018, **15(Suppl 2):**O94

**Introduction:** Considering the ISHCSF report (2013) on comorbidities in idiopathic Normal Pressure Hydrocephalus (iNPH), we studied the relationship between outcome and four scores: The Comorbidity Index (CMI) proposed by Kiefer in 2006, the Cumulative Illness Rating Scale Geriatrics (CIRS-G), the Charlson Comorbidity Index (CCI) and, for the preoperative evaluation, the American Society of Anesthesiologists (ASA) physical status classification.

**Methods:** From 2015 to 2017 a multidisciplinary team selected 30 patients for shunt surgery after diagnosis of “probable iNPH” and exclusion of very severe comorbidities. Baseline demographic, cognitive, functional and pathological characteristics were recorded and converted into the “four scores”. A ventriculoperitoneal shunt was positioned in all patients. Outcome evaluation was carried out 6 months later. “Good outcome” was defined by the improvement of modified Rankin Scale (mRS) > 0 and/or iNPH grading scale (INPHGS) > 1 (or return to INPHGS = 0).

**Results:** The “four scores” median values (ranges) were: CIRS-G 9 (4–14); Charlson 0.5 (0–3); Kiefer 2.5 (0–6); ASA 3 (2–3). Good outcome was reported in 23 cases (76.6%); this “good outcome” group generally had lower values of comorbidity scores than the other one, with fewer and/or less severe concomitant diseases, but only for the ASA classification the difference was statistically significant (p < 0.05); ASA 2 was associated with good outcome.

**Conclusions:** In this study population of selected iNPH patients the preoperative ASA physical status classification 2 (mild systemic diseases) was significantly associated to good outcome; a similar trend, but not significant, was observed for CIRS-G, Charlson CI and Kiefer CMI.

## O96 Endoscopic treatment of suprasellar cyst early with hydrocephalus

### Jiaping Zheng, Qing Xiao, Guoqiang Chen, Yiyang Huang, Li Qizhuang

#### Neurosurgery Center, Aviation General Hospital of China, Beijing, China

##### **Correspondence:** Guoqiang Chen

*Fluids and Barriers of the CNS* 2018, **15(Suppl 2):**O95

**Purpose:** The incidence of suprasellar cysts is approximately 9–21% in children with arachnoid cysts. It is generally believed that the occupying effect of suprasellar cysts is an important cause of hydrocephalus. We have investigated the endoscopic treatment of hydrocephalus with small suprasellar cyst and insignificant mass effect.

**Methods:** Neurosurgery in our hospital performed between August 2016 and August 2017 for 10 patients with 3 months to 15 months. There were 6 males and 4 females. Their bregma tension increased and development was delayed. MRI of the head revealed dilatation of the ventricles, no obstruction in the aqueduct of sylvius, and flat or slightly raised bulges at the base of the third ventricle. ETV were given, and the midbrain lobe and pontine lobe of lilliequist membrane were fistulized at the same time.

**Results:** Postoperative fontanelle tension decreased in 9 patients. Magnetic resonance imaging revealed a decrease in ventricular volume. One patient’s symptoms did not improve significantly and he was treated with ventriculoperitoneal shunt.

**Conclusion:** For patients with imaging findings suggesting that there is no clear obstruction in the ventricular system, the basal cisterna matrices and the morphology of the basement of the third ventricle should be carefully observed. If the basal cistern is full, the flat or microvolume of the third ventricle may indicate the possibility of suprasellar cysts. Endoscopic treatment can relieve the basal cistern obstruction, relieve hydrocephalus, and reduce shunt dependence.

**Keywords:** Hydrocephalus, Arachnoid cyst, Suprasellar cistern, Neuroendoscopy

## O97 Comparison of complication and revision rates after frontal versus parietal approach for ventricular shunt placement in normal pressure hydrocephalus

### Lorenzo Rinaldo, Giuseppe Lanzino, Benjamin D. Elder

#### Department of Neurosurgery, Mayo Clinic, Rochester, Minnesota, United States

##### **Correspondence**: Benjamin D. Elder

*Fluids and Barriers of the CNS* 2018, **15(Suppl 2):**O96

**Introduction:** There is limited information on the effect of frontal versus parietal shunt placement on complication and revision rates after shunting for normal pressure hydrocephalus (NPH).

**Methods:** Patients with NPH receiving shunts between 2001 and 2017 were included for analysis. The effect of frontal versus parietal shunt placement on the incidence of complications and revision surgery was assessed using the Pearson’s Chi squared or Fisher’s exact test and logistic regression analysis.

**Results:** There were 348 patients included for analysis, with 266 (76.4%) and 82 (23.6%) patients receiving a frontal and parietal shunt, respectively. The incidence of intracerebral hemorrhage (ICH), subdural (SD) fluid collections, and SD fluid collections requiring surgical evacuation was 1.7%, 19.9%, and 5.7%, respectively. The rate of revision surgery was 21.0%, with a rate of proximal catheter malpositioning or obstruction of 1.7% and an infection rate of 3.7%. There were no differences in the rate of ICH (1.1% vs 3.7%; p = 0.146), SD fluid collection formation (17.7% vs 26.8%; p = 0.071), and SD collection requiring evacuation (5.6% vs 6.1%; p = 0.877) between patients with frontal versus parietal shunts. There was no difference in the rate of revision surgery (21.8% vs 18.3%; p = 0.495), either due to proximal obstruction/malpositioning (1.1% vs 3.7%; p = 0.146) or infection (3.8% vs 3.7%; p = 0.966), between frontal versus parietal shunts.

**Conclusions:** We did not observe differences in complication or shunt revision rates between shunts placed through a frontal versus parietal approach in our institutional series.

## O98 Effect of fixed-setting versus programmable valve on incidence of shunt revision after ventricular shunting for normal pressure hydrocephalus

### Lorenzo Rinaldo, Giuseppe Lanzino, Benjamin D. Elder

#### Department of Neurosurgery, Mayo Clinic, Rochester, Minnesota, United States

##### **Correspondence:** Benjamin D. Elder

*Fluids and Barriers of the CNS* 2018, **15(Suppl 2):**O97

**Introduction:** We compared shunt revision rates between patients receiving a fixed-setting (FSV) versus programmable valve (PV).

**Methods:** Patients with NPH treated with ventricular shunting between 2001 and 2017 were included for analysis. The incidence of shunt revision was noted and risk factors for revision were identified using a Cox proportional hazards model.

**Results:** There were 348 patients included for analysis, with 98 patients (28.1%) receiving a PV. Shunt revision occurred in 73 patients (21.0%), with 12 patients (3.4%) undergoing multiple revisions. Overall revision rates were lower in patients receiving a PV (13.3% vs 24.0%; p = 0.027), and all patients undergoing multiple revisions initially received a FSV. Patients with a PV were less likely to undergo revision due to persistent symptoms without obstruction (2.0% vs 8.8%; p = 0.032). On multivariate analysis, increasing age (Unit RR 0.93, 95% CI 0.90–0.96; p = 0.001) and PVs (RR 0.18, 95% CI 0.01–0.90; p = 0.035) were associated with reduced risk of distal obstruction, and PVs were associated with reduced risk of revision due to persistent symptoms without obstruction (RR 0.26, 95% CI 0.04–0.91; p = 0.032). PVs were associated with more frequent shunt series during follow-up (4.1 vs 1.0 x-rays/follow-up year; p < 0.001), but not more frequent head CT scans (4.8 vs 3.9 CTs/follow-up year; p = 0.260).

**Conclusion:** Our results suggest that programmable valves lead to reduced rates of shunt revision in patients with NPH. Despite the increased cost of PVs, they may be cost-effective.

## O99 Predictors of distal obstruction after ventriculoperitoneal shunt placement for normal pressure hydrocephalus

### Lorenzo Rinaldo, Giuseppe Lanzino, Benjamin D. Elder

#### Department of Neurosurgery, Mayo Clinic, Rochester, Minnesota, United States

##### **Correspondence:** Benjamin D. Elder

*Fluids and Barriers of the CNS* 2018, **15(Suppl 2):**O98

**Introduction:** Distal obstruction is a common cause of shunt failure and need for revision in patients undergoing ventriculoperitoneal shunting (VPS) for normal pressure hydrocephalus (NPH).

**Methods:** Records of patients with NPH treated with VPS between 2001 and 2017 were reviewed. The incidence of revision surgery due to distal obstruction was noted. Risk factors for distal obstruction were identified using a stepwise Cox proportional hazards model.

**Results:** There were 341 patients included for analysis. Assistance from a general surgeon in placement of the peritoneal catheter was provided in 55 patients (16.1%). Shunt revision was necessary in 69 patients (20.2%), with 17 patients (5.0%) found to have a distal obstruction. On univariate analysis, increasing age was associated with reduced risk of distal obstruction (Unit RR 0.92, 95% CI 0.89–0.96; p < 0.001). BMI ≥ 38.9 (RR 6.60, 95% CI 1.84–19.00), prior abdominal surgery (RR 2.95, 95% CI 1.11–7.70; p = 0.032), and fixed-setting valve (RR 6.24, 95% CI 1.27–112.72; p = 0.020) were associated with increased odds of distal obstruction. General surgery involvement had no effect on distal obstruction rates (OR 0.89, 95% CI 0.25–3.21; p = 0.862). On multivariate analysis, increasing age (Unit RR 0.92, 95% CI 0.89–0.95; p < 0.001) and prior abdominal surgery (RR 3.30, 95% CI 1.23–8.71; p = 0.019) were independently associated with decreased and increased risk of distal obstruction, respectively.

**Conclusions:** We identify multiple factors associated with distal shunt obstruction. These data may aid in the risk-stratification of patients undergoing VPS for NPH.

## O100 Effects of physical exercise after shunt surgery in idiopathic normal pressure hydrocephalus (INPHYS)

### Johanna Rydja^1^, Katarina Owen^1^, Lena Kollén^2^,^3^, Elisabet Blomsterwall^2,3^, Per Hellström^3^, Åsa Lundgren Nilsson^4^, Mats Tullberg^3^, Fredrik Lundin^5^

#### ^1^Department of Activity and Health, Linköping University Hospital, Linköping, Sweden; ^2^Department of Occupational Therapy and Physiotherapy, Sahlgrenska University Hospital, Gothenburg, Sweden; ^3^Institute of Neuroscience and Physiology, Sahlgrenska Academy, University of Gothenburg, Sweden; ^4^Rehabilitation Medicine, Institute of Neuroscience and Physiology, Sahlgrenska Academy, University of Gothenburg, Gothenburg, Sweden; ^5^Department of Clinical and Experimental Medicine (IKE), Division of Neuroscience, Linköping University, Sweden

##### **Correspondence:** Johanna Rydja

*Fluids and Barriers of the CNS* 2018, **15(Suppl 2):**O99

**Introduction:** Idiopathic normal pressure hydrocephalus (iNPH) share some clinical features with Parkinson’s disease (PD). Physical activity can promote positive effects on motor symptoms as well as cognitive functions in PD.

In a previous study we used actigraphy for long-time evaluation of physical activity in iNPH patients. In spite of improved motor functions, measured with Timed up and go test (TUG) and 10-m walk test, the patients did not improve in motor parameters collected by actigraphy.

The hypothesis is that adding a structured physical exercise program for iNPH-patients with goal setting after shunt surgery, an increased benefit for the patients regarding different aspects of motor function, cognition, quality of life and activities of daily living (ADL) can be achieved.

**Methods:** Two Swedish centers have included patients from 2016 until 2018: 128 patients were needed according to sample analysis. Consecutive inclusion and randomisation into (1) written advice for physical self-exercise or (2) additional rehabilitation program for 12 weeks, 2 times 60 min a week postoperatively. Primary outcomes: changes in iNPH scale and Goal Attainment Scaling (GAS) before surgery, 3 and 6 months postoperatively. Secondary outcomes: changes in 6-min walk test, TUG, 30-s chair stand test, EQ5D-5L, LiSat11, Beck´s Depression Inventory, Actigraphy, ADL-taxonomy. The examiner is blinded.

**Results:** Inclusion has ended. We are now finishing the intervention and completing the follow up data collections. Final results are expected in 2019.

**Conclusions:** Hopefully this study will provide evidence for an additive effect of physical exercise after shunt surgery in iNPH.

## O101 LP shunt for iNPH patients: surgical technique

### Naoyuki Samejima, Nobumasa Kuwana, Akira Watanabe, Akira Sato, Yojiro Seki

#### NPH Center, Department of Neurosurgery, Tokyo Kyosai Hospital, Japan

##### **Correspondence:** Yojiro Seki

*Fluids and Barriers of the CNS* 2018, **15(Suppl 2):**O100

**Introduction:** The SINPHONI-2 study (a group of Japanese prospective multicenter cohort studies of the treatment of idiopathic normal pressure hydrocephalus [iNPH]) was carried out and showed the safety and efficacy of LP shunt surgery for iNPH. The LP shunt has recently become widely used in the treatment of iNPH in Japan. Although our high level of success with surgery may be considered to be a minor point, it is worth reporting, as minor differences in technique and know-how can markedly affect the efficacy of shunt surgery. We show a video of our LP shunt procedure.

**Methods:** A total of 429 probable iNPH patients underwent LP shunt surgery at our NPH center between April 2009 and December 2016 (mean age of 78.1 ± 6.5 years). Aspects of our surgical technique include: (1) general anesthesia, (2) use of the original drape, (3) upward insertion of the spinal tube through L2/3 via a paramedian puncture for highly deformed lumbar spine patients, (4) placement of a Codman-Hakim programmable valve with Siphonguard™ in the back, (5) inclination of the table at 35° angle without position change and re-sterilization, (6) laparotomy via rectal muscle splitting, and (7) running the peritoneal tube obliquely from the upper lateral to lower medial (which is different from the route used for catheter installation and for abdominal entry to eliminate the space permitting catheter expulsion).

**Results:** The LP shunt could be placed in all 429 cases without changing its route to the VP shunt. Of the 394 patients followed up at the NPH Center for 1 year after LP shunt surgery, 260 (66%; 95% CI 60–71) showed a favorable outcome, defined as improvement of at least one point on the modified Rankin scale. During the first year after surgery, 37 of 394 patients (9.4%) developed postoperative complications including chronic subdural hematoma requiring evacuation in 13 patients (3.2%), tube occlusion in 7, lower limb numbness in 5, migration of the abdominal tube in 5, rupture of the spinal tube in 2, and shunt infection in 2.

**Conclusions:** Our LP shunt procedure generally seems to be acceptable from the viewpoint of complications. The low-invasive LP shunt that does not require ventricular puncture is preferred and has become the first-line procedure for iNPH in Japan. We would like to popularize the use of this surgical procedure worldwide in the future.

## O102 Comparison between gravitational and flow-regulated shunt valves in the treatment of normal pressure hydrocephalus (flow-grav study)

### Pierre Scheffler, Markus Oertel, Lennart Stieglitz

#### Department of Neurosurgery, University Hospital Zurich, Switzerland

##### **Correspondence:** Lennart Stieglitz

*Fluids and Barriers of the CNS* 2018, **15(Suppl 2):**O101

**Introduction:** Normal pressure hydrocephalus (NPH) is frequently treated with ventriculoperitoneal (VP) shunt surgery. However, VP shunt implantation can lead to overdrainage and complications such as headaches, hygroma and subdural hematoma due to a siphon effect in upright position. Gravitational valves were designed to prevent overdrainage through position-dependent adjustment of valve resistance. Unfortunately, in our experience, gravitational valves occasionally cause underdrainage, requiring subsequent valve explantation. Flow-regulated valves, which increase flow resistance in presence of a high transvalvular pressure gradient, are an alternative to gravitational valves that may provide similar protection against overdrainage without causing underdrainage.

**Methods:** We retrospectively compared gravitational valves with flow-resistance valves in patients with NPH. The primary endpoint was the occurrence of hygroma or subdural hematoma. Secondary endpoints were response to shunt therapy (Black Grading Scale ≥ 5) and frequency of valve adjustments and reoperations.

**Results:** Seventy three patients were included in this interim analysis. No significant difference in the postoperative occurrence of hygroma and subdural hematoma (5.7% for gravitational valves vs 10% for flow-regulated valves, p = 0.51) or the response to treatment (81.1% vs 85%, p = 0.7) was found. There was a significant difference in the average number of valve adjustments per patient between both groups (2 vs 0.7, p < 0.001) and a trend towards a lower rate of surgical revisions in the flow-regulated valve group (0.26 vs 0.05, p = 0.1).

**Conclusion:** Our results suggest that implanting a flow-regulated valve instead of a gravitational valve may lead to fewer valve adjustments and reoperations in patients undergoing VP shunt implantation for NPH.

In submitting an abstract to the to the International Society for Hydrocephalus and Cerebrospinal Fluid Disorders I warrant, on behalf of myself and any co-authors, that:

## O103 Facial memory is impaired in normal pressure hydrocephalus

### Suraj Sennik, Linda D’Antona, Claudia L. Craven, Lewis Thorne, Laurence D. Watkins, Ahmed Toma

#### Department of Neurosurgery, National Hospital for Neurology and Neurosurgery, London, United Kingdom

##### **Correspondence:** Suraj Sennik

*Fluids and Barriers of the CNS* 2018, **15(Suppl 2):**O102

**Introduction:** Idiopathic Normal Pressure Hydrocephalus (iNPH) patients have known cognitive deficits in addition to gait disturbances and urinary incontinence. Improvement in cognitive tests after lumbar drainage of cerebrospinal fluid is part of the diagnostic evaluation for possible iNPH patients. We review scores in cognitive subtests of a routinely used cognitive battery, to determine which were the most sensitive for iNPH.

**Methods:** Retrospective analysis of iNPH cohort of 52 patients who underwent Wechsler Adult Memory Scale-Third Edition (WAIS-III). Scores are assessed against published normal values for Alzheimer’s disease and intact patients [1].

**Subjects:** Fifty two patients with iNPH who undertook the WAIS-III

**Results:** Median test scores in the WAIS-III facial memory subtest was 5.0 (n = 52, test result range 0–90). Facial memory deficit was the most consistent finding in the test battery, and was lower than median scores published for intact patients (32.3, n = 98) and Alzheimer’s disease (26.9, n = 46). Word recall and pneumonic fluency were also found to be impaired, concurring with data already published on neurocognitive profile in iNPH2.

**Conclusion:** Facial recognition scores in cognitive tests may be a sensitive tool to differentiate NPH patient. Facial recognition could be useful addition to cognitive test batteries used to predict shunt responsiveness.

**References**

1. Seelye AM, Howieson DB, Wild KV, Moore MM, Kaye JA. Wechsler Memory Scale-III Faces test performance in patients with mild cognitive impairment and mild Alzheimer’s disease. J Clin Exp Neuropsychol. 2009;31(6):682–8.
2. Saito M, Nishio Y, Kanno S, et al. Cognitive profile of idiopathic normal pressure hydrocephalus. Dement Geriatr Cogn Disord Extra. 2011;1(1):202–11. 10.1159/000328924.

Comparison of WAIS-III Facial Recognition Scores in our NPH cohort vs. literature intact (n = 98)^1^ AD (n = 46)^1^ NPH (n = 52) 32.3, 26.9, 5.0

## O104 Formulating infection control guidelines for lumbar drain insertion following subarachnoid haemorrhage

### Suraj Sennik, Claudia L Craven, Linda D’Antona, Hassan Asif, Simon Thompson, J. Ramos, Will Dawes, Lewis Thorne, Laurence D. Watkins^1^, Ahmed K. Toma

#### National Hospital for Neurology and Neurosurgery, Queen Square, London, United Kingdom

##### **Correspondence:** Suraj Sennik

*Fluids and Barriers of the CNS* 2018, **15(Suppl 2):**O103

**Introduction:** CSF Diversion via lumbar drain (LD) is relatively common following subarachnoid haemorrhage (SAH). We review a single centre’s outcomes with the aim to formulate a putative guideline for LD insertion and infection control.

**Method:** Retrospective cohort of patients admitted to a single centre with SAH with a lumbar drain inserted (January 2016–January 2018).

**Results:** Twenty-four patients (13.1% of SAH admissions), aged 58.3 ± 18.7 (mean ± SD) underwent LD insertion.

Od the 24, only 1 post LD insertion CSF sample grew an organism (*S. epidermis*) and this was on enriched culture only. No patient in this cohort demonstrated systemic features of infection.

Neither location of drain insertion (ITU/HDU n = 14, theatre n = 7, angiography suite n = 3), seniority of operator (resident n = 18; consultant n = 6) or drain material (Silverline^®^ Spiegelberg n = 16; barium-impregnated EDM Medtronic n = 8) had any significant impact on infection rate.

White cell count was raised in 23 of the 24 lumbar samples (411 ± 844 WCC/μL; 1009 ± 1590 RBC/μL).

In 7 patients who underwent subsequent ventricular CSF diversion following LD removal (EVD n = 1, VP shunt n = 6), a rostro-caudal WCC gradient of 29.33 ± 37.44 to 163 ± 217 WCC/μL was observed. None of the 7 patients developed an intracranial infection.

**Conclusion:** Lumbar drains can be safely inserted on ITU by residents without increasing infection risk. Lumbar white cell count is not a useful marker of infection following SAH. Antibiotic’s should be used only in patients with clear organism growth.

## O105 Analysis of categorization based on intracranial pressure monitoring in idiopathic intracranial hypertension, CSF leak, and mixed CSF disorders

### Alexander Perdomo-Pantoja^1^, Riccardo Serra^1^, Rajiv R. Iyer^1^, Lacie Manthripragada^1^, Aruni Rao^2^, Abhay Moghekar^2^, Mark Luciano^1^

#### ^1^Department of Neurosurgery, Johns Hopkins University, Baltimore, Maryland, United States; ^2^Department of Neurology, Johns Hopkins University, Baltimore, Maryland, United States

##### **Correspondence:** Alexander Perdomo-Pantoja

*Fluids and Barriers of the CNS* 2018, **15(Suppl 2):**O104

**Introduction:** Evaluation of patients with suspected idiopathic intracranial hypertension (IIH) and CSF leak by lumbar puncture (LP) demonstrated limitations in patients with borderline pressures and mixed disorders. ICP monitoring (ICPM), though more invasive, may offer higher accuracy, continuous measurements and positional testing.

**Methods:** Charts of patients with suspected pressure abnormalities who underwent continuous ICPM at our institution between 2015 and 2018 were reviewed, and pressures collected. Patients were grouped as suspected IIH, CSF leak or mixed disorders, and subcategorized on ICPM positional testing as high, normal or low pressures. Differences between LP, lying and positional ICPM were analyzed.

**Results:** 119 patients underwent ICPM procedures. The overall mean pressure was 16.01 mmHg with LP and 10.09 mmHg with lying ICPM (p ≤ 0.0001). In the suspected IIH subgroup, a significant difference was found between LP and lying ICPM (p = 0.0004). In this group high pressures were demonstrated in 82% of LPs but only 12% with lying ICPMs. In the low-pressure CSF leak subgroup, LP and lying ICPM pressures were statistically different to standing ICPM (p ≤ 0.0001, 0.003) In groups suspected of CSF leaks, the positional ICPM increased detection of low pressures from 1 to 11/28 (39%) patients. ICPM helped ruling out abnormalities in 36/41 (88%) and 18/23 (78%) of IIH and CSF leak suspected cases.

**Conclusions:** LP pressures often differ significantly from ICPM measurements, especially with the addition of positional testing. ICPM allowed for the sub-categorization of IIH and CSF leaks, validating high and low pressures and ruling out abnormalities in many patients.

## O106 A novel gravity-driven flow model to study ventriculo-peritoneal shunting

### Ricardo Serra, Noah Leviton Gorelick, Rajiv Iyer, Mark Luciano

#### Department of Neurosurgery, Johns Hopkins University, Baltimore, Maryland, United States

##### **Correspondence**: Ricardo Serra

*Fluids and Barriers of the CNS* 2018, **15(Suppl 2):**O105

**Introduction:** Hydrocephalus, a diffuse and multifaceted disease, constitutes a significant challenge to investigators: the varied etiology, complex pathophysiology and high rate of therapeutic failure frequently lead to poor outcomes. The limitations of current therapeutic strategies prompted us to develop a system capable of testing and supporting the development of novel shunting technologies.

**Methods:** We designed a gravity-driven and temperature-controlled shunting system contained inside a tissue-culture-grade air-tight incubator. A set of different adjustable valves were tested to mimic ventriculo-peritoneal shunting over a period of 30 days, with two daily trials of 8 and 14 h. Diurnal positional changes were recreated by adjusting collecting reservoir height, while intracranial pressure was maintained within a normal physiologic range. Valves were submerged in a water bath to simulate the elastic recoil of subcutaneous tissue and the system was filled with normal saline solution.

**Results:** Physiological flow rates were recorded continuously over a 30-day period, showing inter-trial reproducibility and significant flow rate differences between vertical and supine states. The reliability of the proposed system was further investigated by means of hourly measurements, showing a constant and pressure-related change in flow rates during each trial.

**Conclusions:** We have designed and tested a novel gravity-driven model of shunting: this system will allow for a more accurate and reliable testing of catheters and valves, providing a realistic pathophysiologic reproduction of ventriculoperitoneal shunting. We believe that this line of research will contribute to significant improvements in the management of CSF disorders.

## O107 Revision rates of three different types of programmable shunt valve in VA shunt for the treatment of iNPH

### Kiyoshi Takagi^1^, Kazuyoshi Kato^2^

#### ^1^NPH Center, Tokyo Neurological Center Hospital, Edogawa, Tokyo, Japan; ^2^Department of Surgery, Abiko Seijinkai Hospital, Abiko, Chiba, Japan

##### **Correspondence:** Kiyoshi Takagi

*Fluids and Barriers of the CNS* 2018, **15(Suppl 2):**O106

**Introduction:** CSF shunt can treat iNPH but the complications are not negligible. Shunt revision in VA shunt has been reported 6.7% (Clin Neurol Neursurg 2017;157:1–6). However, it has not yet been known whether the type of shunt valve influences the rate of shunt revision. We retrospectively investigated whether the revision rate depends on the shunt valve type.

**Methods:** From April 2007 to December 2017, 467 iNPH patients received VA shunt were followed up over 1 year. The revision rates were compared among 3 different types of programmable valve; Valve A (StrataII, n = 98), Valve B (CHPV-SG, n = 182), and Valve C (SPVA-140, n = 56). Results were shown in mean (SD). Statistical analysis was performed by ANOVA or Chi square test. Statistically significant level was set at p < 0.05.

**Results:** The mean ages for 3 types of valve were 77.8 (6.1) yo (Valve A), 77.3 (6.6) (Valve B), and 77.4 (6.8) (Valve C) (p = 0.8356). Revision rates were 13.3%, 5.5%, and 0% respectively (p = 0.0109). Symptomatic improvement was observed in 34 cases (73.9%) after shunt revision.

**Conclusions:** This study clearly demonstrates that the rate of shunt revision depends of the type of programmable valve. Among these 3 valves, only Valve C lacks anti-siphon device. Although anti-siphon device is used to prevent over drainage, the result of the investigation indicates that it enhances the incidence of shunt revision. Shunt system is composed of inflow catheter, valve, anti-siphon device, and outflow catheter. There are many combinations and further investigations are indispensable to find the vest combination.

## O108 Endoscopic third ventriculostomy for adults with hydrocephalus: a systematic review and meta-analysis

### Sondre Tefre, Alexander Lilja-Cyron, Marianne Juhler

#### Department of Neurosurgery, Rigshospitalet University Hospital of Copenhagen, Denmark

##### **Correspondence:** Sondre Tefre

*Fluids and Barriers of the CNS* 2018, **15(Suppl 2):**O107

**Introduction:** Research on endoscopic third ventriculostomy (ETV) is overwhelmingly based on paediatric populations for whom the ETV success score (ETVSS) exist to aid patient selection. The ETVSS is not applicable for adults, as it includes neither adult age ranges nor common aetiologies of adult hydrocephalus. We performed a systematic review of the literature on ETV for adult hydrocephalus and discuss the need for an adult ETVSS.

**Methods:** Publications on adult ETV were identified in MEDLINE and EMBASE, and selected for the review based on pre-specified inclusion and exclusion criteria. Meta-analysis was conducted for complications as well as for success rates grouped by aetiology and shunt history.

**Results:** Twenty nine papers were included in the review. The best outcomes were seen for non-communicating hydrocephalus with success rates ranging from 79.8 to 83.1%, followed by haemorrhagic aetiology at 75.7%. Normal pressure hydrocephalus and infection showed success rates of 49.1% and 45.6% respectively. Previously shunted patients fared significantly worse than those without prior treatment (64.5% vs. 78.9%). The overall complication rate was 5.4% and procedure-related mortality 0.4%.

**Conclusions:** While certain patient groups are treated effectively with ETV, low success rates are seen for others; and, although rare, serious complications occur. Proper patient selection is therefore paramount, and we believe that an adult ETVSS should be created. More research is required to determine appropriate outcome factors, but a meta-analytical approach serves as a good background for future studies.

## O109 The affect of acetazolamide on intracranial pressure: primary study with prolonged continuous intracranial pressure monitoring

### Simon D. Thompson^1^, Huan Wee Chan^2^, Ahmed K. Toma^1^, Lewis Thorne^1^, Laurence D. Watkins^1^

#### ^1^Victor Horsely Department of Neurosurgery, National Hospital for Neurology and Neurosurgery, London, United Kingdom; ^2^Department of Neurosurgery, Queens Medical Centre, Nottingham, United Kingdom

##### **Correspondence:** Simon D. Thompson

*Fluids and Barriers of the CNS* 2018, **15(Suppl 2):**O108

**Introduction:** Acetazolamide has frequently been used as a first-line treatment for idiopathic Intracranial Hypertension (IIH) and other disorders leading to raised intracranial pressure (ICP). The effect of Acetazolamide has been observed through lumbar puncture, however the effect of Acetazolamide on ICP has not been studied in continuous ICP measurement.

**Methods:** A retrospective study of a prospectively built ICP database was undertaken. All patients with continuous ICP monitoring demonstrating 24 h on and 24 h off Acetazolamide were included in the study. Patients median ICP and median pulse amplitude over 24 h monitoring period on and off Diamox was assessed.

**Results:** 12 patients (9F, 3M) underwent ICP monitoring with data collected during the same admission. 8 patients had IIH, 1 Chiari Malformation, 3 new diagnostic ICP procedures. Seven patients were started on Acetazolamide following raised ICP on primary monitoring, 5 were on Acetazolamide during the first period of monitoring and subsequently stopped and more data collected.

10 patients saw a reduction in ICP while on Acetazolamide. Overall, patients experienced a Median reduction of 1.14 mmHg (mean 1.16 mmHg, range 4.24 to − 4.445 mmHg). Patients (n9) who were on ≥ 1 g of Acetazolamide per day experienced a median reduction of 1.595 mmHg (mean 1.91 mmHg, range 4.24–0.5 mmHg).

**Conclusion:** Our data suggests Acetazolamide can reduce ICP quickly following commencement, however this reduction was relatively small. The effect seems greater with a higher dose. Larger numbers of patients are required to gain a greater understanding into the significance of acetazolamide on ICP, particularly the affect at larger doses.

## O110 Separation of cardiac- and respiratory-driven CSF motions based on realtime phase contrast magnetic resonance and time-resolved frequency analyses

### Tetsuya Tokushima^1^, Satoshi Yatsushiro^2,3^, Mitsunori Matsumae^4^, Hideki Atsumi^4^, Kagayaki Kuroda^1,2^

#### ^1^Course of Electrical and Electronic Engineering, Graduate School of Engineering, Tokai University, Hiratsuka, Kanagawa, Japan; ^2^Department of Human and Information Science, School of Information Science and Technology, Tokai University, Hiratsuka, Kanagawa, Japan; ^3^BioView Inc., Tokyo, Japan; ^4^Department of Neurosurgery, Tokai University, Isehara, Kanagawa, Japan

##### **Correspondence:** Tetsuya Tokushima

*Fluids and Barriers of the CNS* 2018, **15(Suppl 2):**O109

**Introduction:** To separate cardiac- and respiratory-driven CSF motions under free breathing, short-time Fourier transform (STFT) and Stockwell Transform (ST) were applied to CSF velocity waveforms obtained by realtime, asynchronous phase contrast magnetic resonance imaging.

**Methods:** Five patients of hydrocephalus [3 males (38, 74, and 76 yo), 2 females (14 and 70 yo)] were examined under the following conditions: TR, 6.0 ms; TE, 3.9 ms; flip angle, 10 degrees; slice thickness, 7 mm; acquisition matrix, 256 × 256; SENSE factor, 4; velocity encode direction, FH; and velocity encoding (VENC), 10 cm/s. The time resolution was 217 ms. STFT was performed with 8-s long hamming window. Window length of ST was changed adaptively with the frequency component.

**Results:** Cardiac-driven components were detected between 1 Hz and 1.5 Hz in most of the subjects. In 2 patients, the cardiac frequency was higher than the others by 0.3–0.6 Hz. One of them had tumor in anterior horn and the other had obstruction of Sylvian aqueduct. Respiratory components, which are likely between 0 and 0.5 Hz, were not clearly recognized in most of the subjects.

**Conclusions:** The cardiac components of CSF motions were separately detected by STFT and ST under free breathing. The cardiac component had different frequency bands in each patient. In order to detect the weak respiratory components spreading over the low frequency bands, optimization of parameters for the transformation techniques is necessary.

## O111 Mortality among individuals with pediatric hydrocephalus

### Hannah M. Tully^1,4^, Jennifer Phillips^2^, Daniel Doherty^2,4^

#### ^1^Division of Pediatric Neurology, University of Washington/Seattle Children’s Hospital, Seattle, United States; ^2^Research Informatics, Seattle Children’s Enterprise Analytics, Seattle, United States; ^3^Department of Pediatrics, University of Washington/Seattle Children’s Hospital, Seattle, United States; ^4^Center for Integrative Brain Research, Seattle Children’s Research Institute, Seattle, United States

##### **Correspondence:** Hannah M. Tully

*Fluids and Barriers of the CNS* 2018, **15(Suppl 2):**O110

**Introduction:** Pediatric hydrocephalus is reputedly associated with high mortality, but the extent to which death is due to hydrocephalus as opposed to other medical comorbidities is unclear.

**Methods:** We systematically assessed a cohort of children treated for hydrocephalus at a regional medical center between 2009 and 2018, using existing clinical data supplemented by official state death records. We assessed causes of death, calculated mortality rates, and compared hazard ratios (HRs) in various subgroups.

**Results:** Among 2154 individuals with childhood-onset hydrocephalus, 159 (7.4%) were deceased, with an overall mortality rate of 9.1 per 1000 person-years (95% CI 7.8, 10.1). Mortality rates were more than 6 times higher in children diagnosed after their first birthday compared to those diagnosed before (32.6 vs 4.9 per 1000 person-years (95% CIs: 26.1, 40.6; 3.8, 6.2, respectively), primarily reflecting the preponderance of fatal brain tumors in the older age group. Among 1420 children diagnosed with hydrocephalus during infancy, 68 (4.8%) were deceased, and cause of death could be determined in 65. Death was primarily attributable to medical comorbidities such as complications of prematurity in 54 (83%). Among the 14 deaths associated with elevated intracranial pressure, 6 involved catastrophic shunt failure. The remaining 8 deaths were attributable to a decision not to pursue surgical treatment due to perceived futility.

**Conclusions:** Death among children with hydrocephalus is primarily due to medical comorbidities rather than hydrocephalus itself. Among individuals diagnosed during infancy who died of elevated intracranial pressure, death was attributable to shunt failure and to decisions to forgo surgical treatment.

## O112 Resting-state-functional MRI (F-RSMRI) in patients affected by probable idiopathic normal pressure hydrocephalus (iNPH): changes in the connectivity networks after tap test. A preliminary prospective study to improve patient selection

### Francesco Tuniz^1^, Marta Maieron^3^, Daniele Bagatto^2^, Maria C. Vescovi^1^, Maria C. De Colle^2^, Enrico Belgrado^4^, Miran Skrap^1^

#### ^1^Neurosurgery Department, ASUI S.M. della Misericordia, Udine, Italy; ^2^Neuroradiology Department, ASUI S.M. della Misericordia, Udine, Italy; ^3^Physics Department, ASUI S.M. della Misericordia, Udine, Italy; ^4^Neurology Department, ASUI S.M. della Misericordia, Udine, Italy

##### **Correspondence:** Francesco Tuniz

*Fluids and Barriers of the CNS* 2018, **15(Suppl 2):**O111

**Introduction:** Resting-state functional MRI (rsMRI) studies have shown alterations in connectivity within the default-mode-network (DMN) to be associated with cognitive impairments in several disorders. The aim of this prospective study is to understand if rsMRI could improve the positive predictive value of the invasive tests for selecting patients for shunt surgery.

**Methods:** A total of 12 consecutive patients with diagnosis of probable iNPH were submitted to a diagnostic MR examination before and immediately after a lumbar infusion test and tap test. 7 subjects were positive to lumbar infusion test (group 1) while 5 patients were negative (group 2). All the MR examinations included a T1w-mprage and a rsMRI SS-EPI (200 vol). Functional data were processed by FSL using MELODIC-ICA and analysis was performed with GLM by dual-regression, p < 0.05. The ICA-component-dataset was inspected to identify the classical functional pattern. Differences in rsMRI data were assessed within and between group 1 and 2 and in a cohort of healthy volunteers.

**Results:** In group 1 we found a significant positive difference from pre and post tap-test for motor network (p < 0.043, Z = 5.4), language network (p < 0.052, Z = 4.5) and DMN (p < 0.048, Z = 8.7). The analysis performed within group 2 pre and post tap-test don’t show any improvement. The analysis of only the post tap-test-rsMRI acquisition in both groups showed an improvement in the motor network (p < 0.058, Z = 4.5), language network (p < 0.06, Z = 4.1) and DMN (p < 0.057, Z = 3.5).

**Conclusions:** Since the trend of rsMRI agreed with invasive test results this could be a promising method to be considered for the management of iNPH patients candidates for shunt surgery.

## O113 Chronic pulmonary diseases and outcome of inph shunted patients

### Tiziana Urli^1^, Carlo Bortolotti^2^, Giorgio Palandri^2^, Monica Nicola^2^, Paolo Mantovani^2^, Vito A. Piserchia^1^, Sabina Cevoli^3^, Giulia Giannini^4^, David Milletti^5^, Luca Albini Riccioli^6^, Carmelo Sturiale^2^, Pietro Cortelli^4^

#### ^1^Anesthesia and Intensive Care, IRCCS Istituto Scienze Neurologiche Bologna, Italy; ^2^Department of Neurosurgery, IRCCS Istituto Scienze Neurologiche Bologna, Italy; ^3^Department of Neurology, IRCCS Istituto Scienze Neurologiche Bologna, Italy; ^4^DIBINEM, University of Bologna, Italy; ^5^Rehabilitation Medicine, IRCCS Istituto Scienze Neurologiche Bologna, Italy; ^6^Department of Neuroradiology, IRCCS Istituto Scienze Neurologiche Bologna, Italy

##### **Correspondence:** Tiziana Urli

*Fluids and Barriers of the CNS* 2018, **15(Suppl 2):**O112

**Introduction:** Lung-brain interactions are partly already known, such as the oxygen delivery process, partly matter of recent studies, such as the cognitive impairment in chronic obstructive pulmonary diseases or the brain-lung cross-talk in neurocritically ill ventilated patients. The aim of this study was to test the relationship between chronic pulmonary diseases and outcome in patients with idiopathic Normal Pressure Hydrocephalus (iNPH).

**Methods:** From 2015 to 2017 a multidisciplinary team selected 30 patients for shunt surgery after diagnosis of “probable iNPH”. Baseline demographic, cognitive, physical and pathological characteristics were recorded. Very severe comorbidities were considered as exclusion criteria. Diagnosis of pulmonary disease was done considering history, symptoms and chest x-ray. A ventriculoperitoneal shunt was positioned in all patients. Outcome evaluation was carried out 6 months later, considering one generic scale, the modified Rankin Scale (mRS) and one specific scale, the iNPH grading scale (INPHGS). Good outcome was defined by improvement of mRS > 0 and/or INPHGS > 1 (or return of INPHGS to 0).

**Results:** Chronic pneumopathies were present in 11 patients (36.6%). Overall “good outcome” was reported in 23 cases (76.6%). The absence of a pulmonary disease was significantly associated with improvement of mRS (p < 0.05), while the association with INPHGS was not significant. The relation between outcome and other comorbidities (cardiac, vascular, musculoskeletal, urinary, endocrine-metabolic, neurological, psychiatric) was not significant (p > 0.1).

**Conclusions:** In this study pulmonary diseases were associated with the outcome described by the modified Rankin Scale. Pulmonary comorbidities should be considered in future studies on the outcome of iNPH shunted patients.

## O114 Biomechanical approach of brain aging, neurodegenerative diseases and frailty

### Alexandra Vallet^1,3^, Armelle Lokossou^2^, Sylvie Lorthois^3^, Pascal Swider^3^, Pauline Assemat^3^, Laurent Risser^4^, Zofia Czosnyka^5^, Marek Czosnyka^5^, Natalia Del Campo^6^, Laurent Balardy^6^, Patrice Peran^1^, Olivier Balédent^2^, Pierre Payoux ^1^, Eric Schmidt^1,6^

#### ^1^ToNIC UMR 1214, INSERM, Toulouse, France; ^2^CHIMERE EA 7516, Amiens, France; ^3^Inst. De Mecanique des Fluides de Toulouse UMR 5502, Toulouse, France; ^4^Inst. De Mathématiques de Toulouse UMR 5219, Toulouse, France; ^5^Neurosurg. Unit, Department of Clinical Neurosciences, United Kingdom; ^6^Ctr. Hospitalier Universitaire de Toulouse, Toulouse, France

##### **Correspondence:** Alexandra Vallet

*Fluids and Barriers of the CNS* 2018, **15(Suppl 2):**O112

**Introduction:** Brain aging is a natural process that can become pathological leading to neuronal loss and neurodegenerative diseases. In link with CSF related disorders, intracranial physical constrains like stress, strain or shear should participate to the pathophysiology of neurodegenerative diseases. We propose to explore the frail zone that is the transition region from normal to pathological brain aging with a biomechanical approach.

**Methods:** A statistical analysis was performed on a database that included 100 patients suspected of normal pressure hydrocephalus with enlarged ventricles or parenchymal atrophy, gait disturbance, modest cognitive decline or urinary incontinence. The frailty was evaluated using the SEGA score, based on cognitive status, nutritional status, risk of depression, level of independence and fall risk.

The cerebrospinal fluid (CSF) dynamics was explored using an infusion test. The intra cranial pressure was recorded while a saline fluid was injected at constant rate through a lumbar puncture. The intracranial fluids (blood and CSF) dynamics were also quantified (at baseline) with phase contrast MRI. A model of the blood and CSF circuit system was fitted on the clinical measurements in order to obtain the brain mechanical properties.

**Results:** The statistical analysis showed a significant correlation (r = 0.34, p = 0.01) between brain elastance, which describes the brain ability to accommodate to volume changes, and frailty index SEGA.

**Conclusion:** Our results support the hypothesis that biomechanical characterization of the brain could be valid to identify the transition from normal to pathological aging.

## O115 Cerebral perfusion measured by arterial spin labeling MRI does not increase after shunt surgery in patients with iNPH

### Johan Virhammar^1^, André Ahlgren^2^, Kristina G. Cesarini^3^, Katarina Laurell^4^, Elna-Marie Larsson^5^

#### ^1^Department of Neuroscience, Neurology, Uppsala University, Sweden; ^2^Center for medical image science and visualization, Linköping University, Linköping, Sweden; ^3^Department of Neuroscience, Neurosurgery, Uppsala University, Sweden; ^4^Department of Pharmacology and Clinical Neuroscience, Unit of Neurology, Östersund, Umeå, University, Sweden; ^5^Department of Surgical Sciences, Radiology, Uppsala University, Sweden

##### **Correspondence:** Johan Virhammar

*Fluids and Barriers of the CNS* 2018, **15(Suppl 2):**O114

**Introduction:** Cerebral blood flow (CBF) has been reported to increase after shunt surgery in patients with idiopathic normal pressure hydrocephalus (iNPH). Using the non-invasive perfusion MRI method arterial spin labeling (ASL), the aims of this study was to investigate if CBF increases after shunt surgery, if postoperative change in CBF correlates with reduction in ventricular volume and improvement in symptoms, and if baseline CBF data correlates with postoperative outcome.

**Methods:** Twenty-three patients with iNPH were prospectively included and examined clinically and with MRI of the brain at baseline. Eighteen of the patients were treated with shunt implantation and were reexamined clinically and with MRI 3 months post-operatively. The MRI protocol included pseudo-continuous ASL for perfusion imaging and Synthetic MR as volumetric method. The perfusion was measured in twelve manually drawn ROIs.

**Results:** In the whole sample, CBF did not increase after shunting in any ROI. Postoperative reduction in ventricular volume correlated with increased CBF in: periventricular white matter (r = 0.56, p < 0.05), frontal medial cortex (FMC) (r = 0.72, p = 0.01), lentiform nucleus (r = 0.61, p < 0.05) and thalamus (r = 0.52, p < 0.05). Preoperative CBF in FMC was correlated with an improvement in incontinence after shunt surgery, r = 0.53, p < 0.05. However, there were no correlations between change in CBF and change in clinical symptoms postoperatively.

**Conclusions:** The use of ASL MRI may have limited clinical value, as it could not predict outcome after shunt surgery and there were no correlations between change in CBF and change in clinical symptoms after shunt surgery. However, more longitudinal perfusion studies are needed.

## O116 Cerebral aqueduct CSF stroke volume hydrodynamics in chronic communicating hydrocephalus: exploration of the initial and late phases in an animal model

### Tito Vivas-Buitrago^1,5^, Armelle Lokossou^6^, Ignacio Jusué-Torres^8^, Gabriel Pinilla-Monsalve^1^, Jamie Robison^1^, Ari M Blitz^3^, Daniel A. Herzka^9^, Jiadi Xu^4^, Hugo Guerrero-Cazares^5^, Susumu Mori^3,4^, Alfredo Quiñones-Hinojosa^5^, Olivier Baledént^6,7^, Daniele Rigamonti^1,2^

#### ^1^Johns Hopkins School of Medicine, Department of Neurosurgery, Baltimore, United States; ^2^Johns Hopkins Aramco Healthcare, Dhahran, Saudi Arabia; ^3^Johns Hopkins School of Medicine, Department of Radiology, Baltimore, United States; ^4^Kennedy Krieger Institute, Kirby Research Center for Functional Brain Imaging, Baltimore, United States; ^5^Mayo Clinic Florida, Department of Neurosurgery, Jacksonville, Florida, United States; ^6^Chimère EA 7516, Research Team for head & neck, University of Picardie Jules Verne, Amiens, France; ^7^Jules Verne University Hospital, Department of Image Processing, Amiens, France; ^8^Loyola University School of Medicine, Department of Neurosurgery, Maywood, Illinois, United States; ^9^Johns Hopkins School of Engineering, Department of Biomedical Engineering, Baltimore, United States

##### **Correspondence:** Daniele Rigamonti

*Fluids and Barriers of the CNS* 2018, **15(Suppl 2):**O115

**Introduction:** Phase-contrast-magnetic-resonance-imaging (PCMRI) is a non-invasive technique of measuring CSF hydrodynamics, which is often used to assess and differentiate patients with normal pressure hydrocephalus (NPH) from other conditions with ventricular enlargement. Although controversial, it has been reported that hyperdynamic Aqueductal-CSF-Stroke-Volume (ASV) is associated with better CSF diversion treatments in NPH patients. It is critical to better understand NPH physiopathology and hydrodynamics during early development. This study aims to elucidate the ASV dynamics during NPH early development and throughout progression of the disease.

**Methods:** Chronic communicating hydrocephalus was induced in adult Sprague–Dawley-rats by injecting Kaolin into the subarachnoid space over the cerebral convexities and compared with control animals. PCMRI was performed to calculate the ASV as well as T2-W images for ventricular size measurements at days 15, 60, 90 and 120 using a Bruker-11.7-TMR. Non-parametric tests were implemented to analyze ASV/ventricular volumes (VV) correlation.

**Results:** Kaolin injected (KI) animals showed significant ventricular enlargement at all time points. Strong difference in ASV was present between KI and controls at all times. Significant VV/ASV correlation 15 (p = 0.015), 60 (p = 0.001), 90 (p < 0.001) and 120 days (p < 0.001) was found. Moreover, there was a significant positive correlation between the ventricular volume expansion and the ASV between 15 and 60 days.

**Conclusions:** An initial active phase of rapid ventricular enlargement shows a strong VV/ASV correlation during the first 60 days, followed by a second phase with less further ventricular enlargement and heterogeneous ASV. Results may suggest an optimal window for CSF diversion treatment that will be assessed in a following experiment with shunted animals.

## O117 White matter tracts fractional anisotropy and mean diffusivity changes over time in a rodent model of chronic communicating hydrocephalus

### Tito Vivas-Buitrago^3^, Gabriel Pinilla-Monsalve^1^, Ignacio Jusue-Torres^2^, Jamie Robison^1^, Alfredo Quiñones-Hinojosa^3^, Susumu Mori^4^, Daniele Rigamonti^1,2^

#### ^1^Department of Neurosurgery, Johns Hopkins Hospital, Baltimore, United States; ^2^Johns Hopkins Aramco Healthcare, Dhahran, Saudi Arabia; ^3^Mayo Clinic Florida, Department of Neurosurgery, Jacksonville, United States; ^4^Department of Radiology, Johns Hopkins Hospital, Baltimore, United States

##### **Correspondence:** Daniele Rigamonti

*Fluids and Barriers of the CNS* 2018, **15(Suppl 2):**O116

**Introduction:** Diffusion tensor imaging (DTI) has been used to characterize microstructural alterations of the brain parenchyma. The onset of this microstructural damage in iNPH patients is unknown given our current limitations in the early identification of this syndrome before symptoms presentation. This study aims to explore the fractional anisotropy (FA) and mean diffusivity (MD) in the white matter tracks (WM) during the early development and progression of chronic communicating hydrocephalus in a rodent model.

**Methods:** Communicating chronic hydrocephalus was induced in five adults Sprague–Dawley rats by a bilateral kaolin injection into the subarachnoid space over the convexities and compared with controls. DTI acquisition was obtained with a Bruker 11.7-Tscan. Regional FA and MD were measured at the corpus callosum (CC) and cortical-spinal tracts (CST) at days 15, 60, 90 and, 120 after injection.

**Results:** Progressive ventricular enlargement was found only in the injected group at all four-time points (p < 0.050). MD at the CC was significantly higher in hydrocephalic animals at all times: 14 (p = 0.0278), 60 (p = 0.0143), 90 (p = 0.009), and 120 days (p = 0.0139). CC-MD showed a positive correlation with the ventricular volume (p ≤ 0.013), as well as a significant progressive increase in value over time (p = 0.0070). CC-FA was decreased in the hydrocephalic animals only at 90 days (p = 0.0150). No differences were found in CST-FA at any time point.

**Conclusions:** Higher CC-MD was found in the hydrocephalic animals at all times. These findings will be further correlated with behavioral analysis and symptoms recovery rates in treated/shunted animals at different time points to assess microstructural variation/recovery at the corpus callosum.

## O118 Characteristics of the first 500 subjects in the adult hydrocephalus clinical research network registry

### Michael A. Williams^1^, Mark Luciano^2^, Sean J. Nagel^3^, Norman Relkin^4^, Thomas Zwimpfer^5^, Heather Katzen^6^, Richard Holubkov^7^, Mark G. Hamilton^8^

#### ^1^Departments of Neurology and Neurological Surgery, University of Washington, Seattle, United States; ^2^Department of Neurosurgery, Johns Hopkins University, Baltimore, United States; ^3^Department of Neuro-restoration, Cleveland Clinic, United States; ^4^Department of Neurology, Weill Cornell School of Medicine, New York, United States; ^5^Department of Surgery, University of British Columbia, Vancouver, Canada; ^6^Department of Neurology, University of Miami, United States; ^7^Department of Family & Preventive Medicine, University of Utah, Salt Lake City, United States; ^8^Department of Neurosurgery, University of Calgary, Canada

##### **Correspondence:** Michael A Williams

*Fluids and Barriers of the CNS* 2018, **15(Suppl 2):**O117

**Introduction:** The characteristics of the adult population with hydrocephalus are not well described. The Adult Hydrocephalus Clinical Research Network (AHCRN), founded in 2014, initially comprised University of Calgary, University of British Columbia, Cleveland Clinic, Weill Cornell Medical College, Sinai Hospital of Baltimore, University of Washington, and University of Utah (Data Coordinating Center).

**Methods:** Adults ≥ 18 years were non-consecutively enrolled in a registry. Data includes symptoms, examination, neuropsychology screening, comorbidities, imaging, treatment, complications, and outcomes. Four categories were defined: Transition (treated before age 18), Congenital (congenital pattern, not treated before age 18), Acquired (secondary to known risk factors, treated or untreated), and Possible Idiopathic Normal Pressure Hydrocephalus (≥ age 65 years, not previously treated). We report the first 519 subjects (2015–2017).

**Results:** Demographics

- Sex: female 42.0%, male 58.0%
- Age at enrollment: 59.8 ± 19.2 years (range 18.1–90.7)
- Race: White 90.6%, Asian 5.0%, Black 1.7%, missing/other 2.7%
  Treatment at time of enrollment
- All subjects: 36.8% (shunt 26.8%, ETV 10.0%)

**Table:** 

	Transition	Congenital	Acquired	Possible iNPH
N	N = 86	N = 132	N = 88	N = 213
(%)	16.6%	25.4%	17.0%	41.0%
Category variation among 6 centers	0–43%	0–42.2%	8.7–31.9%	19.0–85.7%
Treatment at time of enrollment	100% Shunt 95.3% ETV 14.0%	43.2% Shunt 22.0% ETV 21.2%	39.2% Shunt 27.5% ETV 11.8%	0% By definition
Top etiologies	Myelomeningocele: 33.7% Aqueductal stenosis: 15.1% IVH of prematurity: 9.3%	Communicating: 41.7% Aqueductal stenosis: 50.0% Aqueductal pattern: 15.2%	Brain tumor: 28.4% Intraventricular adhesion/web/colloid cyst: 13.7% Non-traumatic SAH: 11.8% TBI: 9.8%	
Lawton ADL score (lower = better)	3.1 ± 4.0	1.5 ± 2.7	4.1 ± 5.9	5.5 ± 5.2

**Conclusions:** The population in the AHCRN registry is diverse. A combined 42% have childhood onset, whether treated or untreated by age 18, and only 41% are possible INPH. The degree of impairment (Lawton ADL) is least in the congenital group and worst in possible INPH. The proportion of hydrocephalus categories varied widely among centers. More research on the lifespan needs and outcomes for adult hydrocephalus is needed.

Supported by the hydrocephalus association

## O119 Importance of quantification in changes of symptoms and images after shunting in iNPH

### Shigeki Yamada^1,2^, Masatsune Ishikawa^1,3^, Makoto Yamaguchi^2^, Kazuo Yamamoto^2^

#### ^1^Normal-Pressure Hydrocephalus center, Rakuwakai Otowa Hospital, Kyoto, Japan; ^2^Department of Neurosurgery, Rakuwakai Otowa Hospital, Kyoto, Japan; ^3^Rakuwa Villa Ilios, Kyoto, Japan

##### **Correspondence:** Shigeki Yamada

*Fluids and Barriers of the CNS* 2018, **15(Suppl 2):**O118

**Introduction:** It is not fully recognized how symptoms and intracranial CSF distribution change after shunt surgery in iNPH. To reduce the misunderstanding that shunt surgery is ineffective for iNPH, we should objectively and quantitatively evaluate the improvement of symptoms and CSF imaging after shunting. Therefore, we quantitatively assessed the change of gait disturbance and CSF distribution in the patients with iNPH.

**Methods:** Eighty-five patients (mean age, 76 years) with iNPH who underwent 3-T T2-weighted 3D-MRI before and after shunting. We measured Evans index, z-Evans index, brain/ventricle ratios (BVR) and callosal angle, and the volumes of total ventricle, convexity-SAS, Sylvian fissure and basal cistern and posterior fossa-SAS. Additionally, we measured the time and score on instrumented timed up-and-go test (iTUG) before and after shunting by using the newly released free iPhone application “hacaro iTUG”.

**Results**: The mean time on iTUG was shortened by > 5 s and the mean iTUG score was increased by > 20 points 2–4 weeks after shunting. Maximum changing rates about 6 months after shunting were as follows; Evans index: − 3%, z-Evans index: − 9%, BVR: + 28%, callosal angle: + 34%, total ventricle volume: − 16%, convexity-SAS volume: + 39%, volume of Sylvian fissure and basal cistern: − 5%, and posterior fossa-SAS volume: + 8%.

**Conclusions:** The iTUG score rather than iTUG time measured by hacaro iTUG is a universally applicable measurement for the quantitative assessment of gait disturbance in iNPH. CSF distribution specific to iNPH gradually normalized around 6 months after shunting. The most distinct morphological change was increase in the volume of convexity-SAS.

## O120 Cerebrospinal fluid dynamics: MRI time-spatial spin labeling

### Shinya Yamada^1^, Seiko Shimizu^2^

#### ^1^Neurosurgery, Toshiba Rinkan Hospital, Kanagawa, Japan; ^2^Canon Medical systems, Tochigi, Japan

##### **Correspondence:** Shinya Yamada

*Fluids and Barriers of the CNS* 2018, **15(Suppl 2):**O119

**Introduction:** Time spatial labeling inversion pulse (Time-SLIP) technique was developed that permits CSF dynamics observation over a 1–5 s period. Using this method, we have investigated the CSF dynamics in normal and pathophysiological conditions.

**Methods:** MRI scanners (Canon Medical Systems Corp., Japan) was used for Time-SLIP CSF dynamics imaging. Using a scan time of 3–6 min, Time-SLIP was applied to CSF where the region of interest.

**Results:** The Time-SLIP technique revealed the CSF physiological aspects that had not been known. CSF reflux from the third ventricle to the lateral ventricle in the normal brain was confirmed. This reflux disappeared when hydrocephalus developed. This observation is totally the opposite finding from what we had seen on radioisotope study or CT cisternography. This CSF exchange shows the existence of active CSF exchange between the lateral ventricle and the third ventricle in the normal brain. CSF turbulent flow in the third and fourth ventricle that has also been discovered by this technique may play a very important role regarding volume transmission function of the CSF to the circum ventricular organs. Real time Time-SLIP imaging showed importance of respiration effect on CSF motion besides cardiac pulsation. Head motion also drives CSF motion that implies difference of CSF dynamics while awake and during sleep.

**Conclusions:** We visualized CSF dynamics using MRI Time-SLIP method. The CSF kinetics observed by the Time-SLIP method in normal physiological brain were very different from the description of the neurosurgical textbook.

## O121 Percutaneous-tunneled, transfontanellar external ventricular drainage as a bedside early treatment of post hemorrhagic hydrocephalus in ELBW infants

### Mino Zucchelli, Francesca Nicolini, Mariella Lefosse

#### Department of Pediatric Neurosurgery, IRCCS Istituto delle Scienze Neurologiche Bologna, Italy

##### **Correspondence:** Mino Zucchelli

*Fluids and Barriers of the CNS* 2018, **15(Suppl 2):**O120

**Introduction:** Optimal treatment of hydrocephalus affecting ELBW newborns is still debated: although an early treatment correlates with better neurological outcome, the choice of surgical strategy must take into account the frailty of these patients. We present a safe, quick and well tolerated bedside procedure that can achieve this purpose.

**Methods:** Fourteen cases of posthemorrhagic hydrocephalus (PHH) in ELBW infants (7 cases < 700 g, range 550–1000 g) were treated with a PTTEVD that was implanted at bedside as the first measure in a stepwise approach. Cognitive and motor outcome according to Griffith scales at 12 and 24 months of follow/up will be discussed.

**Results:** The average duration of the procedure was 7 min, and there was no blood loss. The drain remained in place for an average of 24 days (range 8–45 days). In all cases early control of the hydrocephalus was achieved. One patient had a single episode of CSF leakage (due to insufficient CSF removal). In another patient Enterococcus in the CSF sample was detected the day after abdominal surgery with ileostomy (infection resolved with intrathecal vancomycin). One patient died of Streptococcus sepsis, a systemic infection existing prior to drain placement that never resolved. Once a patient reached 1 kg in weight, when necessary, a ventriculoperitoneal shunt was implanted and the PTTEVD was removed.

**Conclusions:** The introduction of PTTEVD placement in our standard protocol for the management of PHH has proved to be a wise temporary option for small patients that could impact over the long-term outcome.

## O122 Shunt malfunction: add, replace or remove

### Mino Zucchelli, Francesca Nicolini, Ercole Galassi

#### Pediatric Neurosurgery, IRCCS Istituto Scienze Neurologiche di Bologna, Italy

##### **Correspondence:** Mino Zucchelli

*Fluids and Barriers of the CNS* 2018, **15(Suppl 2):**O121

**Introduction:** Shunt malfunction still represents a common problem for the pediatric and general neurosurgeon. In any case the choice between replacing the shunt, performing a secondary third ventriculostomy (sETV) with removal of the shunt or implanting a new catheter should aim to resolution of symptoms entailing the lowest operative risks.

**Methods:** We applied to 122 cases of shunt malfunction a simple protocol taking into account the age of the patient, the presence of ventricular enlargement compared with the previous neuroradiological examinations and the technical feasibility of a sETV.

Forty seven patients underwent sETV and 75 shunt revision. We employed another treatment protocol in the latter group with the use of intraluminal coagulation, endoscopic release of the adherent catheter, and the placement of a new catheter with neuronavigation as ultimate strategy.

**Results:** The overall success rate of sETV was 74% (shunt free patients with normalized intracranial pressure and resolution of symptoms) also in patients with a long shunting duration (up to 30 years). The number of previous shunt revision procedures (p = 0.026) and lower age (p = 0.017) correlates with the likelihood of secondary ETV failure, a score of 80 in ETV success score correlates with secondary ETV success (p = 0.014). None of the cases case of the group was complicated by postoperative intracranial hemorrhage

**Conclusion:** The application of our treatment protocol to patients with CSF shunt malfunction allowed, in a large number of cases, shunt removal by performing sETV; when it didn’t seem feasible, the endoscopic management of the intraventricular catheter avoided significant operative complications.

## O123 Improvement of gait and cognitive function 3 months after endoscopic third ventriculostomy (ETV) in adult obstructive hydrocephalus

### Tom J. Zwimpfer^1,8^, Rich Holubkov^2^, Heather Katzen^3,8^, Mark G. Luciano^4,8^, Ben Miller^2^, Sean J. Nagel^5,8^, Michael A. Williams^6,8^, Mark G. Hamilton^7,8^

#### ^1^Department of Surgery, University of British Columbia, Vancouver, Canada; ^2^Department of Pediatrics, University of Utah, Salt Lake City, United States; ^3^Department of Neurology, University of Miami, United States; ^4^Department of Neurosurgery, Johns Hopkins University, Baltimore, United States; ^5^Department of Neuro-restoration, Cleveland Clinic, United States; ^6^Departments of Neurology and Neurological Surgery, University of Washington, Seattle, United States; ^7^Department of Clinical Neurosciences, University of Calgary, Canada; ^8^Adult Hydrocephalus Clinical Research Network (AHCRN)

##### **Correspondence:** Mark G. Hamilton

*Fluids and Barriers of the CNS* 2018, **15(Suppl 2):**O122

**Introduction:** In addition to symptoms of raised ICP, adults with obstructive hydrocephalus often present with gait, bladder and/or cognitive dysfunction. This report presents preliminary results of gait and cognitive function, both pre and 3 months post ETV in adults.

**Methods:** Adult patients with obstructive hydrocephalus were identified based on tri-ventricular hydrocephalus on CT and MRI. This report focuses on (1) gait velocity (10 m timed gait) and; (2) cognitive function (Montreal Cognitive Assessment [MOCA]) pre and post ETV.

**Results:** Eighty five adults underwent ETV, with a mean age of 55 years and 49 (58%) males. Distribution of hydrocephalus: 41 (48%) Congenital, never previously treated; 33 (39%) Acquired, shunted or not; 11 (13%) Transitional patients, any etiology and treated before age 18. ETV was primary in 73 (86%), while 12 (14%) with a blocked shunt, underwent a secondary ETV. Due to drowsiness, confusion, severe gait disturbance, 49 (58%) of the 85 patients completed a MOCA and Gait test prior to the ETV. To date, 33 (67%) of these 49 have a Post ETV MOCA, with improvement from 24/30 (2 months pre ETV) to 26/30 (3 months post ETV). Post ETV Gait tests were completed by 32 of the 49 (65%) with improvement of gait velocity from 0.9 m/s (1 months pre ETV) to 1.2 m/s (3 months post ETV).

**Conclusions:** ETV in adults with obstructive hydrocephalus results in improvement of cognitive and gait, 3 months post ETV. Longer follow-up will determine the degree and durability of this improvement. Supported by the Hydrocephalus Association.

## P1 The risk of subdural fluid collection following high volume lumbar puncture for the evaluation of idiopathic normal pressure hydrocephalus

### Naomi A. Abel, Alexey Goloubev, Yarema Bezchlibnyk, Siviero Agazzi

#### Department of Neurosurgery and Brain Repair, University of South Florida, Tampa, Florida, United States

##### **Correspondence:** Naomi A. Abel

*Fluids and Barriers of the CNS* 2018, **15(Suppl 2):**P1

**Introduction:** High volume lumbar puncture (LP) with rapid removal of cerebral spinal fluid (CSF) in the outpatient setting has a high positive predictive value for shunt responsive idiopathic normal pressure hydrocephalus (iNPH). To evaluate gait, patients stand up shortly after the high-volume LP raising concern for development of subdural fluid collection. While the reported rate of subdural fluid collection following insertion of a ventricular shunt is 10 percent and is often initially asymptomatic, there is no data for the rate of subdural fluid collection after high volume LP. The objective of this study is to investigate the potential for early occurrence of subdural fluid collection following high volume LP and gait assessment.

**Methods:** Thirty-four sequential patients age 59–94 years old with clinical presentation consistent with probable iNPH and Evans Index greater than 0.33 underwent LP with removal of 30–40 cc of CSF using a 22-gauge 3.5-inch Quincke spinal needle. Laboratory results and medications were reviewed minimizing risk of coagulopathy. Gait was evaluated at 60 min following the LP and a non- contrast computed tomography (CT) of the head was performed 3–4 h later. Patients were queried for the presence of headache post LP and at the 2 weeks follow up appointment.

**Results:** No headaches were reported. There were no early subdural fluid collections following high volume LP and gait assessment.

**Conclusions:** In the outpatient setting, high volume LP and post procedural gait assessment do not increase the risk of early development of subdural fluid collection.

## P2 “Flow-controlled” valves: a technical nonsense term and utopia

### Alfred Aschoff

#### Department of Neurosurgery, Formerly University of Heidelberg, Germany

##### **Correspondence:** Alfred Aschoff

*Fluids and Barriers of the CNS* 2018, **15(Suppl 2):**P2

**Introduction**: In 1987 Sainte Rose cointed the term “flow-controlled valves” for the Orbis-Sigma (OSV), which word is used in the meantime for the Phoenix-Diamond (1995; PD) und das Codman SiphonGuard (1998; SG) also. Inspite of 32 years clinical use (PD 23, SG 20 a) the clinical as well the lab literature is extremly limitated. In 2018 PubMed shows 30 OSV-papers only, one over Diamond, and 9 over SG,

**Material:** 1988–2010 we tested 34 valves (8 designs) with “flow-control”, 31 new probes, 3 explanted, 12 OSV, 4 OSV II, 2 Cordis NPH, 3 SG prototypes, 5 serial SG, 2 with Micro-Programmable, 2 Diamond and 4 Diamond-II. The specimen was submitted up to 35 subtests which lasted over the maximum of 500 days.

**Results:** No valve showed a flow-sensor. All specimen was exclusively controlled by differential pressure (DP). In contrast to normal physiology to ASDs or g-valves the body position has no influence on the hydraulics. The pressure-flow-graphs are flat, similar to sticking slit-valves (e.g. Holter). Critical in OSV is the tiny slit (0.08 mm!) cylinder vs. rubyring, which trends to frictions, extreme temperature- and protein-sensivity. The SG measures 0.4 mm Ø ID only and trends to occlusions. Using 8–15 cmH_2_O pressure the flow exceeds 150 mL/h (19.3% cSDH! Sundström 2017); the flow is too low between 25 and 50 cmH_2_O.

**Conclusions**: CSF-pumps controlled by electric flowsensors are feasible, but need energy and effort. Unclear is the desired flowrate. A pressure-controlled monitoring is necessary.

## P3 History of gravitational valves

### Alfred Aschoff

#### Department of Neurosurgery, Formerly University of Heidelberg, Germany

##### **Correspondence:** Alfred Aschoff

*Fluids and Barriers of the CNS* 2018, **15(Suppl 2):**P3

**Introduction:** Differential-pressure-valves are unable to compensate the hydrostatic pressure off-set in vertical and overdrain. In 1973 Portnoy introduced the Antisiphon device (ASD), a diaphragma-switch activated by the weight of the hanging distal water column. Unfortunately, ASDs are at risk for disturbances due to external pressures. In 1975 Hakim patented the first gravitational (g)-valve with inbuilt spheres, which counteract the gravity effects.

**Methods:** We present personal informations of Portnoy, Schulte, Pudenz, Holter, S. and C. Hakim, Chhabra and Miethke. 1984–2010 we overviewed 1108 g-valves in 822 patients, tested 204 specimens (11 types).

**Results**: A successful clinical study of Hakim-Lumbar was interrupted by an airplan crash of the initiator. The concept remained forgotten except of patents of Yamada, Sainte-Rose, Holter, Portnoy. In 1989 Richard and our team tested simultaneously the Hakim-Lumbar with excellent results. In 1991 we proposed to combine adjustable and g-valves. In 1993 we implanted a customized Hakim-Lumbar and an adjustable Medos. In the same year the Chhabra-Z-Flow and the Affeld-valve, a precursor of the Miethke DualSwitch, were patented. In 1996 we proposed adjustable g-valves, which were launched in 2008 (ProSA). Currently 35 g-designs exist, 12 of them (from 5 companies) are on the market. Clinical g-valve-papers show massively reduced quotes of overdrainage. The most important problems are implantation-failures (not strictly vertical).

**Conclusions:** Gravitational valves have largely solved the problems of overdrainage. The adaptation on the growth and difficult patients require adjustable g-valves. A vertical position is the condition sine qua non for a correct function.

## P4 CSF diversion strategies for persistent cranial pseudomeningocoeles

### Claudia L. Craven, Hasan Asif, Simon Thompson, Joana Ramos, Linda D’Antona, Lewis Thorne, Laurence D. Watkins, Ahmed K. Toma

#### National Hospital for Neurology and Neurosurgery, Queen Square, London, United Kingdom

##### **Correspondence:** Claudia L. Craven

*Fluids and Barriers of the CNS* 2018, **15(Suppl 2):**P4

**Introduction:** Pseudomeningocoeles are a challenging complication of cranial surgery. Rarely, cases can persist beyond simple initial management strategies and require permanent CSF diversion. Such patients are occasionally referred to this specialist hydrocephalus unit. We describe our experience of referrals and management strategies.

**Methods:** Retrospective review of patients with persistent cranial pseudomeningocoeles referred to a single centre’s hydrocephalus specialists from August 2017 to May 2018.

**Results:** Fourteen patients (10F:4M) aged 47.1 ± 20.4 years (mean ± SD) had persistent pseudomeningocoeles after cranial surgery (4 posterior fossa craniotomies, 7 convexity craniotomies and 3 foramen magnum decompression).

Prior to referral, the 14 patients had undergone a collective total of 333 interventions (over range of 2–6 months) including aspiration (n = 7), wound repair (n = 3), lumbar CSF drainage (n = 11), cystoperitoneal shunt (n = 7) and ventriculoperiteonal (VP) shunt (n = 5).

Failure of pseudomeningocoele resolution was the predominant reason for the multiple interventions. Two patients had additional surgery due to persistent bacterial growth in CSF samples (with heavy growth and light growth of *S. epidermis* respectively). 9 patients had monitoring confirming raised ICP pressure.

After referral, 10 patients underwent insertion of a VP shunt with an additional catheter distal to the adjustable valve, draining the pseudomeningocoele. Four patients had simple VP shunts inserted. All patients had significant reduction in pseudomeningocoele on examination, with radiological resolution of the CSF collection at 81.6 ± 31.9 days post-operatively.

**Conclusions:** Persistent cranial ppseudomeningocoeles are frequently associated with high ICP states and can be definitively treated with VP shunt, commonly with catheter distal to the adjustable valve draining the cyst.

## P5 Aseptic meningitis: does it really exist?

### Claudia L. Craven, Simon Thompson, Joana Ramos, Linda D’Antona, Lewis T. Thorne, Laurence D. Watkins, Ahmed K. Toma

#### National Hospital for Neurology and Neurosurgery, Queen Square, London, United Kingdom

##### **Correspondence**: Claudia L. Craven

*Fluids and Barriers of the CNS* 2018, **15(Suppl 2):**P5

**Introduction:** Raised white cell count (WCC) in lumbar CSF is a commonly used marker of meningitis. The effect of cranial neurosurgery per se on lumbar WCC is not established. At this single centre, many patients undergo ICPM followed by lumboperitoneal shunt (LPS), with lumbar CSF WCC samples taken during insertion. We aim to determine the effect of ICP bolt insertion on lumbar CSF WCC.

**Methods:** Retrospective analysis of lumbar CSF samples in patients who had recently undergone 24-h ICPM.

**Results:** Thirty-three patients (16F:7M) aged 43.31 ± 12.1 years (mean ± SD) had lumbar CSF samples after ICPM. Fourteen had CSF sampled within 6 weeks and 19 after 6 weeks of ICPM. Twenty-five samples were taken during LPS insertion, 5 during lumbar drainage/puncture and 3 during LPS revision. All 33 patients were afebrile at the point of CSF sampling.

The mean lumbar WCC within 6 weeks of ICPM was significantly higher than the mean lumbar WCC after 6 weeks, being 15.4 ± 18.0 and 2.32 ± 1.79 cells/μL respectively. There was no significant increase in RBC. In patients with raised CSF WCC, 60% of raised WCC were predominantly lymphoctyes and 40% predominantly neutrophils. Only one patient grew an organism (*S. aureus*).

**Conclusions:** Lumbar CSF WCC can be raised following minor intracranial surgery, despite no clinical sign of infection. We caution against using lumbar CSF WCC values independently as a marker of infection following neurosurgery.

## P6 Missed nursing care and hydrocephalus: is a good operation enough?

### Jessica Frassineti, Maria Elena Ciocchini, Michela Nanni, Paolo Mantovani, Alessandro Pirina, Carmelo Sturiale, Giorgio Palandri

#### Neurosurgery, IRCCS Istituto Scienze Neurologiche Bologna, Italy

##### **Correspondence:** Jessica Frassineti

*Fluids and Barriers of the CNS* 2018, **15(Suppl 2):**P6

**Introduction:** Missed nursing care is defined as assistance actions that are considered indispensable for patient safety, but which are, in daily practice, partly or completely omitted for various reasons. Such omissions can lead to negative consequences and affect patient’s clinical outcome (increase in mortality and length of stay), as well as having negative implications on the psychophysical conditions of the staff. This aspect has not been investigated yet in the neurosurgical patient, and particularly in patients affected from idiopathic normal pressure hydrocephalus (iNPH).

**Methods:** We propose a prospective study to evaluate which are the most missed treatments in the patient with iNPH, why and how often these cures are lost and impact on outcome. A questionnaire will be administered to the nurses of the neurosurgery department, to identify the most missed treatments in the patient with NPH and for which reasons these cures are lost. A transversal prevalence study will be set to assess the frequency of lost treatment, focusing on the triad of Hakim: gait disorder (and related risk of fall), cognitive disorders (in particular memory deficits and spatio-temporal disorientation) and urinary incontinence. A final prospective cohort study will verify how the lost cures affect the clinical outcomes in the patient with NPH, analyzing correlations with post-operative early complications and follow up clinical evaluation.

**Conclusions:** With this study, we propose to identify critical elements in nursing care of INPH patient. In order to guarantee best possible care, a tailored nursing for iNPH patients is needed.

## P7 Use of adjustable anti-gravity devices in nph patients with delayed post-shunt deterioration

### Jonathan P. Funnell^1,2^, Claudia L. Craven^1^, Linda D’Antona^1^, Lewis Thorne^1^, Laurence D. Watkins^1^, Ahmed K. Toma^1^

#### ^1^Victor Horsley Department of Neurosurgery, National Hospital for Neurology and Neurosurgery, London, United Kingdom; ^2^UCL Medical School, University College London, London, United Kingdom

##### **Correspondence:** Jonathan P. Funnell

*Fluids and Barriers of the CNS* 2018, **15(Suppl 2):**P7

**Introduction:** A subset of idiopathic normal pressure hydrocephalus (NPH) patients respond to VP shunt insertion temporarily. Adjustable anti-gravity devices are designed to control CSF drainage position-induced changes; in the authors’ unit, these devices have been used to achieve controlled overdrainage in temporary shunt responders.

**Methods:** A single-centre retrospective study of patients undergoing shunt valve revision from an adjustable differential pressure valve with fixed anti-siphon (ProGAV + Shuntassistant) to a system incorporating an adjustable anti-siphon valve (ProGAV + ProSA) (April 2013–April 2018). Records were retrospectively reviewed for interventions, clinical and subjective outcomes.

**Results:** Twenty one patients diagnosed with temporary shunt responsive NPH improved on high volume shunt reservoir tap. (10M:11F). Accordingly, they underwent VP shunt valve revision from a ProGAV valve with Shuntassistant to ProGAV valve with ProSA valve. Mean age at first VP shunt insertion was 74.5 ± 7.87 years, with 31.5 ± 16.8 months (mean ± SD) until revision with a ProSA valve. Mean follow up was 31.37 ± 15.85 months.

Of 20 patients with sufficient follow-up, 12 made objective improvements in walking and/or neuropsychological test outcome. 15 patients made subjective improvements following revision in mobility or cognitive impairment.

**Conclusions:** VP shunting with adjustable differential pressure valves and fixed antigravity devices may not drain sufficient CSF for optimum management of low-pressure hydrocephalus. By lowering shunt opening pressure on adjustable antigravity devices to below those of fixed antigravity devices, adequate drainage may be achieved, and outcomes improved in low pressure hydrocephalus.

## P8 Tap test with closure pressure of 0 cm of H_2_O in the diagnosis of normal pressure hydrocephalus in the University Hospital Fundación Santa Fé de Bogotá, 2014–2018

### Diego Gomez A., Maria Cardenas, Maria Dominguez, Juan Davidson, Luis Pulido^1^, Lina Gómez, Juan Ramon, Kemel Ghotme, Juan Armando Mejia, Enrique Jimenez, Fernando Hakim

#### Neurosurgery Section, Department of Surgery, Hospital Universitario Fundación Santa Fe de Bogotá, Bogotá, Colombia

##### **Correspondence:** Diego Fernando Gomez

*Fluids and Barriers of the CNS* 2018, **15(Suppl 2):**P8

**Introduction:** Since the description of normal pressure hydrocephalus (NPH), evacuation lumbar puncture has been a fundamental pillar in the diagnosis of this entity. The positivity of the test has been related to the clinical improvement of the three main manifestations of this disease, which over time has been objectified in various ways. The volume of cerebrospinal fluid (CSF) to be extracted has been described between 30 and 50 cc, without a clear explanation in the literature of the reasons for defining these values. In our institution we have established the volume of CSF extraction based on a closing pressure of 0 cmH_2_O. With this, we are in the search to describe our experience and we propose this parameter as the standard in performing evacuation lumbar puncture for the diagnosis of PNH.

**Methods:** A retrospective review of patients with normal pressure hydrocephalus who were diagnosed by tap test with the pressure methodology of 0 cm of H_2_O at the closing during the period 2014–2018.

**Results:** A total of 77 candidate patients were identified to be included in the study; however, three of them did not have information on the opening pressure or the volume of liquid extracted, for which reason they were excluded from the analysis. Of the 74 patients included, the average age was 79.4 years (SD 7.4 years), with a minimum age of 55 years and a maximum age of 96 years. 47 patients were men (63.5%), the majority of patients are from the city of Bogotá (62.2%). Of the 74 patients analyzed, 95.9% had a confirmed diagnosis of hydrocephalus with normal pressure.

**Conclusions:** Our experience suggests that CSF drainage based on a pressure of 0 cmH_2_O at closure increases the likelihood of a positive result for PNH. The drainage parameters can be standardized and individualized, by a Tap test guided by closing pressure.

## P9 A longitudinal cohort study of idiopathic normal pressure hydrocephalus (iNPH) in the community-dwelling elderly in takAhata, Japan: the prevalence of iNPH in octogenarians

### Chifumi Iseki, Yoshimi Takahashi, Luna Kimihira, Ryusuke Igari, Hiroyasu Sato, Shingo Koyama, Kyoko Suzuki, Takeo Kato

#### Division of Neurology and Clinical Neuroscience, Department of Internal Medicine III, Yamagata University School of Medicine, Japan

##### **Correnponding author:** Chifumi Iseki

*Fluids and Barriers of the CNS* 2018, **15(Suppl 2):**P9

**Introduction:** The prospective study had started in 2000 and recruited 272 out of 350 (77.7%) inhabitants, the total number of 70-year-old in Takahata, a rural area of Japan. We previously estimated the incidence of idiopathic normal pressure hydrocephalus (iNPH) as 1.2/100 persons per year, which was higher than previously thought. We also advocated the state of asymptomatic ventriculomegaly with features of iNPH on MRI (AVIM), as a preclinical stage of iNPH.

**Methods:** The assessment was performed in 2000, 2006, 2010 and 2016–2017 with physical, neurological, laboratory, neuropsychological, and brain MRI exams.

**Results:** By the end of 2016 at the age of 86, 104 subjects had died. Among the living participants, 71 subjects underwent brain MRIs. 104 subjects were evaluated by Clinical Dementia Rating (CDR), where 35 were demented (CDR ≧ 1), in which 7 (6.7%) subjects were diagnosed as having possible or provable iNPH based on the Japanese Guidelines of iNPH; however, any of them had not consulted hospitals by themselves or their family. The longitudinal observation detected that; 3 subjects developed iNPH from AVIM, one became AVIM newly, one remained in AVIM for more than 16 years. Four were non-DESH ventriculomegaly at the age of 70 and became possible iNPH at 86.

**Conclusions:** In the community-dwelling octogenarians, iNPH seems to be common but was underdiagnosed.

## P10 Neurosarcoidosis presenting with normal pressure hydrocephalus: case series

### Aida Kafai, Claudia L. Craven, Lewis T. Thorne, Ahmed K. Toma, Laurence D. Watkins

#### National Hospital for Neurology and Neurosurgery, Queen Square, London, United Kingdom

##### **Correspondence:** Aida Kafai

*Fluids and Barriers of the CNS* 2018, **15(Suppl 2):**P10

**Introduction:** Neurosarcoidosis is a rare condition with a high mortality. Early recognition of symptoms is important to enable prompt interventions. We report a case series of NS with ventriculomegaly that presented with symptoms resembling normal pressure hydrocephalus (NPH).

**Methods:** Case series of patients with ventriculomegaly on MR imaging and diagnosis of confirmed or probable NS.

**Results:** Four patients (2M:2F) aged 49.0 ± 3.01 years (mean ± SD) were identified. Three had definite NS and one probable. Three presented with gait disturbance and one with memory impairment.

MR Imaging (with gadolinium) demonstrated ventriculomegaly with leptomeningeal enhancement. One patient underwent 24-h ICPM, with a median ICP of 3.47 mmHg and pulse amplitude of 4.35 mmHg. CSF showed mild increase in chronic inflammatory cells in the absence of infection.

Patients underwent medical management plus ventriculoperionteal shunt insertion (with adjustable valves set to 5 cmH_2_O and anti-siphon devices). There was a propensity for transient development of slit-like ventricles but without other features of over-drainage. All patients showed symptom improvement following CSF diversion at mean follow-up of 27 months.

**Conclusions:** NS can present similarly to NPH. Early combined treatment of CSF diversion and medical management is effective for symptom management.

## P11 Complication and outcome of VA shunt for idiopathic normal pressure hydrocephalus (iNPH) patients over 85 years old

### Kato Kazuyoshi^1^, Takagı Kiyoshi^2^

#### ^1^Department of Surgery, Abiko Seijinkai Hospital, Japan; ^2^Department of Neurosurgery, Abiko Seijinkai Hospital, Japan

##### **Correspondence:** Takagı Kiyoshi

*Fluids and Barriers of the CNS* 2018, **15(Suppl 2):**P11

**Introduction:** Idiopathic normal pressure hydrocephalus (iNPH) is one of the disabling factors among the elderly. The purpose of this retrospective study is to elucidate the complications and prognosis of VA shunt for the iNPH patients over 85.

**Patients and methods:** From April 2004 to December 2017, 117 iNPH patients over 85 yo received VA shunt. The sequel of VA shunt was investigated in all cases. Outcome was evaluated by mRS in 89 patients 3 months or later after the surgery. The best postoperative mRS was compared with preoperative scale.

**Results:** There were no shunt infection. Three cases died within 3 months by postoperative pneumonia. Symptomatic cerebral infarction occurred in 2 cases within 1 month of surgery. CSDH was observed in 54 cases (46.2%), but only 3 cases (2.6%) needed surgery. Therefore, serious postoperative events occurred in 8 cases (6.8%). The mRS was improved in 55 cases (61.8%), showed no change in 31 cases (34.8%), and worsened in 3 cases (3.4%). The preoperative mRS was 2.8 ± 0.8 and the best mRS after VA shunt was 2.0 ± 1.0. This improvement has statistical significance (p < 0.0001).

**Conclusion:** The occurrance of life-threatening events were low compared to the previous reports (Cerebrospinal Fluid Res 2010;7:18, Lancet Neurol 2015;14:585–94) despite the remarkably older patients covered in this study. The outcome has improved in 62% of cases, and the result was satisfactory enough. Thus it indicates that VA shunt can be performed safely for elderly iNPH patient over 85 yo with good outcome.

## P12 Outcome of lumbo-peritoneal shunt surgery for the patients with idiopathic normal pressure hydrocephalus using virtual off valve

### Takashi Kawahara^1^, Masamichi Atsuchi^1^, Takuya Iwasaki^1^, Kazunori Arita^2^

#### ^1^Division of Neurosurgery, Atsuchi Neurosurgical Hospital, Kagoshima, Japan; ^2^Department of Neurosurgery, Graduate School of Medical and Dental Sciences, Kagoshima University, Japan

##### **Correspondence:** Kazunori Arita

*Fluids and Barriers of the CNS* 2018, **15(Suppl 2):**P12

**Introduction:** Intracranial hypotension, postural headache, and subdural hematoma due to over-drainage, are important complications after lumbo-peritoneal shunt (LPS) in the patients with idiopathic normal pressure hydrocephalus (iNPH). Although shunt devices such as with controllable valve have been developed to prevent these complications, some patients still suffer from this complication even at a highest valve setting. To overcome this complication, shunt devices with a virtual off mode have been developed. We report here a retrospective study of our experience using this kind of shunt with a virtual off mode.

**Methods:** A retrospective study was performed on ninety-five iNPH patient series underwent LPS surgery using Certas Plus valve (Codman^®^) from July 2016 to February 2018 at one hospital. Initial valve pressure of all cases was set at the seven level.

**Results:** After LPS surgery, nine cases (9.4%) presented the symptoms, such as postural headache, subdural effusion, and subdural hematoma. We set the level of programmable valve in the virtual off mode, as soon as the appearance of symptoms. Six cases had improved their headache promptly. Three cases have not improved their symptoms, and we underwent shunt catheter ligation and one was required for hematoma irrigation surgery.

**Conclusions:** The virtual off mode of LPS is useful for the management intracranial hypotension after LPS surgery. However, the symptoms of some cases were not completely resolved, even at the virtual off mode

## P13 Impact of hydrocephalus on the cerebral venous system

### Armelle Lokossou^1^, Mauren Ahiatsi^1^, Pan Liu^1^, Serge Metanbou^2^, Cyrille Capel ^3^, Jadwiga Attier-Zmudka^4^, Olivier Balédent^1,5^

#### ^1^CHIMERE EA 7516, Research Team for head & neck, University of Picardie Jules Verne, Amiens, France; ^2^Department of Radiology, University Hospital, Amiens, France; ^3^Department of Neurosurgery, University Hospital, Amiens, France; ^4^Department of Geriatry, General Hospital, Saint Quentin, France; ^5^Department of Image Processing, University Hospital, Amiens, France

##### **Correspondence**: Armelle lokossou

*Fluids and Barriers of the CNS* 2018, **15(Suppl 2):**P13

**Introduction:** The involvement of cerebral venous system in hydrocephalus condition is still debated. We determined the impact of hydrocephalus on venous vessels morphometry and their flow.

**Methods:** We included 15 hydrocephalic patients (HY, 76 ± 6 years). They were age-matched to 13 Elderly Controls (EC, 74 ± 4 years). Both groups underwent PC-MRI for quantification of right (RJ) and left (LJ) jugular flows and sagittal (SS) and straight (Str.S) sinuses venous flow using *Flow* software. The circularities of these vessels were evaluated with *ImageJ* software. Considering anatomical aspects, we expect sinuses circularity equal to 0.6 which corresponds to a perfect equilateral triangle. For jugular veins, we expect a circularity between 1 (perfect circle circularity) and 0.6. Below these values, the vessel was deformed.

**Results:** For each vessel: Str.S, SS, RJ and LJ venous flow was significantly (p < 0.0005) decreased in HY compared to EC.

SS circularity of HY was significantly decreased (p < 0.05) compared to EC (respectively 0.49 ± 0.2, 0.62 ± 0.1). SS of HY patients is deformed while EC exhibited normal SS circularity.

There was no difference in the Str.S (0.46 ± 0.2; 0.55 ± 0.2) and RJ (0.29 ± 0.1; 042 ± 0.2) circularity between HY and EC. Str.S morphometry was normal in both groups while RJ was deformed in both groups.

LJ circularity of HY was significantly decreased (p < 0.0005) compared to EC (0.35 ± 0.2; 0.61 ± 0.1, respectively). LJ of HY patients was deformed while EC exhibited normal SS morphometry.

**Conclusions:** There is an impact of hydrocephalus on venous flow and morphometry which is reflected by a decrease of flow and vessels deformation.

## P14 Post-traumatic CSF disorders: a comprehensive review

### Romain Manet^1^, Laurent Gergelé^2,3^, Marek Czosnyka^3^, Zofia H Czosnyka^3^, Angelos Kolias^3^, Jacques Luauté^4^, Emmanuel Jouanneau^1^

#### ^1^Department of Neurosurgery B, Neurological Hospital Wertheimer, University of Lyon, France; ^2^Department of Intensive Care, Ramsay Générale de Santé, Hôpital privé de la Loire, Saint Etienne, France; ^3^Division of Neurosurgery, Department of Clinical Neurosciences, Addenbrooke’s Hospital, University of Cambridge, United Kingdom; ^4^Department of Neuro-Rehabilitation, Neurological Hospital Wertheimer, University of Lyon, France

##### **Correspondence:** Romain Manet

*Fluids and Barriers of the CNS* 2018, **15(Suppl 2):**P14

**Introduction**: Cerebrospinal fluid (CSF) flow impairment after traumatic brain injury can results from different pathophysiological mechanisms and lead to different and unclear diagnosis. We summarized current knowledge concerning diagnosis, treatment and prognosis of post-traumatic CSF disorders.

**Materials and methods**: A literature review was conducted, using MedLine database, including prospective and retrospective studies as well as previous reviews.

**Results**: Post-traumatic CSF disorders can be classified in two groups: *post*-*traumatic ventriculomegaly (PTV)* and *post*-*traumatic extra*-*axial CSF collections*. Schematically, PTV can result from post-traumatic atrophy (with normal ICP and normal CSF dynamics) and/or *post*-*traumatic hydrocephalus* (PTH) (with intermittently raised ICP and/or impaired CSF dynamics). PTH may be a frequent condition (incidence 0.7–29%) and has been reported to worsened prognosis of patients. Shunt surgery is generally proposed as reference treatment. Post-traumatic extra-axial fluid collections are probably frequent (incidence 3.7–10%). Two different situations should be distinguished: (i) passive CSF effusions with normal ICP and normal CSF dynamics, that can be classified as *hygroma*, with generally no neurological consequence and spontaneous resolution; (ii) active CSF flow impairment with raised ICP and/or disturbed CSF dynamics, that can be classified as *external hydrocephalus,* wich may have a negative impact on neurological prognosis and should probably treated by shunt surgery.

**Conclusions**: In absence of robust data, management of post-traumatic CSF disorders remains controversial. Further investigations are needed to improve our understanding of pathophysiology and clarified diagnosis, treatment and prognosis. In particular, the value of CSF dynamics analysis should be better investigated.

## P15 Conversion of post-traumatic external hydrocephalus to normal pressure hydrocephalus: an illustrative case

### Romain Manet^1^, Laurent Gergelé^2,3^, Zofia H. Czosnyka^3^, Marek Czosnyka^3^

#### ^1^Department of Neurosurgery B, Neurological Hospital Wertheimer, University of Lyon, France; ^2^Department of Intensive Care, Ramsay Générale de Santé, Hôpital privé de la Loire, Saint Etienne, France; ^3^Division of Neurosurgery, Department of Clinical Neurosciences, Addenbrooke’s Hospital, University of Cambridge, United Kingdom

##### **Correspondence:** Romain Manet

*Fluids and Barriers of the CNS* 2018, **15(Suppl 2):**P15

**Introduction:** Normal pressure hydrocephalus (NPH) is classified as idiopathic (iNPH) when no obvious cause can be identified. Occult traumatic external hydrocephalus could be a *primum movens* of “i”NPH.

**Materials and methods:** Case report.

**Results:** A 74-year-old man presented a progressive neurological decline with cognitive alteration (MMSE = 19/30), headaches and agitation, 10 days after mild traumatic brain injury. We suspected an external hydrocephalus because CT scan showed a subarachnoid spaces enlargement without ventriculomegaly and transcranial doppler (TCD) was in favor of a moderate raised of ICP. As CT scan showed “security signs”, a careful lumbar CSF withdrawal was performed (CSF opening pressure 20 mmHg; withdrawal of 20 mL with no complication). This resulted in an immediate positive effect on neurological status (MMSE = 28/30) and TCD parameters. The patient was discharged home the next day. During 20 months follow-up, patient presented progressive signs of NPH: slight gait and cognitive decline (MMSE = 25/30), ventriculomegaly with periventricular hyper-intensities on MRI and disturbed lumbar CSF dynamics (Rout (constant rate infusion) was 11 mmHg/mL/min; PVI (bolus) was 18 mL; mean pulse amplitude (peak to peak) raised from 2 mmHg (5 min baseline) to 8 mmHg (5 min plateau). A ventriculoperitoneal shunt resulted in fast (within 2 months) and long-lasting (31 months post-op FU) improvement of gait (mountain hiking) and cognitive functions (MMSE = 28/30).

**Conclusions:** Patients with post-traumatic external hydrocephalus can develop delayed NPH, which can be prevented by early shunting. They should receive a careful long-term follow up. Informed consent to publish had been obtained.

## P16 Down syndrome and normal pressure hydrocephalus first case in literature

### Gianpaolo Petrella^1^, Andrea Pietrantonio^1^, Agazio Menniti^1^, Alberto Delitala^1,2^

#### ^1^Department of Neurosurgery, S.Maria Goretti Hospital, Latina, Italy; ^2^Department of Neurosurgery, S.Camillo Forlanini Hospital, Rome, Italy

##### **Correspondence:** Gianpaolo Petrella

*Fluids and Barriers of the CNS* 2018, **15(Suppl 2):**P16

**Introduction:** Down syndrome (DS) was initially described by J. Langdon Down in 1866 and identified as a trisomy of chromosome 21 by Lejeune in 1959. DS is associated with characteristic physical features, deficits in the immune and endocrine systems and delayed cognitive development. A recent report suggests that 75% and 25% of adults with Down syndrome survive up to 50 and 60 years, respectively.

The proportion of the individuals that develop clinical dementia can vary considerably. There is no report regarding the association between normal pressure hydrocephalus (NPH) and a Down syndrome.

**Materials and methods:** We report a case of a 57 years old man with DS, who referred a progressive worsening in his cognitive abilities and gait since 1 year before medical consultation. Because of an epileptic seizure, a head CT scan was performed, showing marked ventricular enlargement, widening of the Sylvian and narrowing of parasagittal fissures and increased Evan’s ratio (more than 0.3). A diagnostic lumbar infusion test (LIT) showed a value of intracranial elastance (IE) of 0.3, thus a ventricular peritoneal shunting was performed (pressure setting at a 140 mmH_2_O).

**Results:** Postoperatively, a marked neurological improvement was noted up to 8 months later, when a progressive neurological deterioration occurred: pressure setting was lowered at 110 mmH_2_O and a new clinical improvement was seen.

**Conclusions:** The association between DS and NPH has never been described; after a thorough clinical, instrumental and radiological evaluation, if NPH is diagnosed, a prompt surgical treatment should be offered. Informed consent to publish had been obtained.

## P17 Guided regulation of hydrocephalus shunts with the new noninvasive method to monitor the intracranial pressure

### Fernando C. G. Pinto^1^, Gustavo Frigieri^2^, Cintya Y. Hayashi^3^, Daniel M. Costa^2^, Sergio Mascarenhas^2^

#### ^1^Cerebral Hydrodynamics Group, Division of Functional Neurosurgery, Institute of Psychiatry, Hospital das Clínicas, University of São Paulo, Brazil; ^2^Braincare Health Tecnology, São Carlos, São Paulo, Brazil; ^3^School of Medicine, Department of Neurology, University of São Paulo, Brazil

##### **Correspondence:** Fernando Campos Gomes Pinto

*Fluids and Barriers of the CNS* 2018, **15(Suppl 2):**P17

**Introduction:** Intracranial pressure (ICP) monitoring is an important guide for the diagnosis, treatment and prognosis of patients. Currently the methods of monitoring this important clinical parameter are invasive, making this measurement impracticable in several applications, for example in the setup of ventriculoperitoneal shunt. This study presents the results of the monitoring using the new noninvasive intracranial pressure sensor (niICP) during the regulation of shunts performed in 10 patients.

**Methods:** Patients with ventriculoperitoneal shunt and symptoms of intracranial hypertension (dizziness, nausea, gait difficulty, urinary incontinence and reduced quality of vision) were monitored with the niICP sensor before and after shunt setup using the same protocol of 5 min of monitoring in the sitting position and 5 min in the lying position at 0 degrees. The data were saved, anonymized and sent for mathematical analysis.

**Results:** The ten patients who with symptoms of intracranial hypertension presented morphology indicating reduction in brain compliance (P2 > P1) before the valve setup in both postural positions. The morphological findings were altered after the shunt regulation procedure, the ICP pulse morphology changed to P1 > P2, indicative of normal values of ICP.

**Conclusion:** Noninvasive monitoring of intracranial pressure is a practical, fast, low cost and efficient tool for the evaluation of patients with hydrocephalus and ventriculoperitoneal shunt.

## P18 Clinical analysis of safety and performance of the Sphera Duo™ fixed pressure valve

### Fernando C. G. Pinto^1^, Rodolfo C. Reis^1^, Matheus F. Oliveira^1^, Carlo E. Petitto^1^, Fabrizzio M. Matrone^1^, Vitor S. Nespoli^1^, João P. S. Castro^1^, Renata H. G. Yamashita^1^, Martha N. B. Morales^1^, Flávia M. G. Pinto1, João V. R. Morais^2^, Manoel J. Teixeira^3^

#### ^1^Cerebral Hydrodynamics Group, Division of Functional Neurosurgery, Institute of Psychiatry, Hospital das Clínicas, University of São Paulo, Brazil; ^2^Instituto Federal de Educação, Tecnologia e Ciência de São Paulo, Brazil; ^3^Department of Neurosurgery, University of São Paulo, Brazil

##### **Correspondence:** Fernando C. G. Pinto

*Fluids and Barriers of the CNS* 2018, **15(Suppl 2):**P18

**Introduction:** The main cerebral hydrodynamics complications in shunted patients are due to malfunction of the system. The Sphera Duo™ is a fixed pressure valve that works through a sequential double coil spring mechanism, seat and ruby sphere. According to the characteristic of the springs, three strips of pressure difference ensure a flow of 21 mL/h: low (3–7 cmH_2_O), medium (7–11 cmH_2_O) and high (11–14 cmH_2_O). The objective of this retrospective, single-center, single-arm cohort study is to confirm safety and performance of Sphera Duo™ when used in adult patients suffering from hydrocephalus (HC), idiophatic intracranial hypertension (IIH) or arachnoid cyst (AC).

**Methods:** The data were generated by reviewing 112 adult patient’s charts who were submitted to a VP shunt surgery for the treatment of cerebral hydrodynamics disturbs (HC, IIH, AC), from November 2014 to May 2017. The Sphera Duo™ was used in all cases. The primary endpoints were frequency and severity of complications or side effects. The secondary endpoints were clinical improvement after 1 year of shunt implantation: resolution of the intracranial hypertension syndrome or improvement of NPH clinical triad.

**Results:** 80.4% of the patients improved the neurological symptons and 8.9% remained stable. The reoperation rate was 15.2% in the first year after surgery.

**Conclusion:** Sphera Duo™ is safe when used in adult patients suffering from HC, IIH, AC. 89.3% of the patients improved the neurological symptons or remained stable. The reoperation rate was 15.2% in the first year after surgery.

## P19 Objectifying gait pattern analysis for the diagnosis of normal pressure hydrocephalus

### Juan F. Ramón^1^, Catalina García-Baena^1^, Daniela Volcinschi^2^, Lina Gómez^1^, Diego Gómez^1^, Fernando Hakim^1^

#### ^1^Neurosurgery Section, Department of Surgery, Hospital Universitario Fundación Santa Fe de Bogotá, Bogotá, Colombia; ^2^Faculty of Medicine, Universidad de los Andes, Bogotá, Colombia

##### **Correspondence:** Juan Fernando Ramón

*Fluids and Barriers of the CNS* 2018, **15(Suppl 2):**P19

**Introduction:** One of the principal alterations characteristically encountered in normal pressure hydrocephalus (NPH) is gait disturbance. It is not only the first symptom found in patients with NPH, but its analysis is also a fundamental aspect used in the diagnosis of the disease. Thus, given that to our knowledge there is not a measurable and quantifiable means of using this feature to diagnose the pathology, we seek to objectify gait pattern analysis by quantitatively determining gait pattern differences with respect to baseline.

**Methods:** We will perform a case–control study including 234 cases with a 1:1 ratio of controls. We will use the programme Kinovea version 0.8.15 to obtain measurements of the following gait parameters: speed, cadence, step length and stride length; each of which will be compared between the two groups. Statistical analysis will include absolute and relative frequencies, as well as central tendencies and dispersion measures. Numerical variables will be compared using parametric t-student or non-parametric U Mann–Whitney tests, according to their normal distribution determined with Shapiro–Wilk test.

**Results:** We expect to find a significant difference between gait parameters obtained in patients versus those encountered in normal subjects.

**Conclusions:** By determining quantifiable measures and differences in gait parameters, we expect to be able to establish specific and characteristic patterns which would allow for better characterisation of the disease. We hope to be able to use these findings to build a diagnostic test which will permit an earlier diagnosis.

## P20 Dolphins in the fishing net: the diagnosis of positional orthostatic tachycardia syndrome (POTS) in patients admitted for presumed cerebrospinal (CSF) hypotension

### Aruna Rao, Lacie Manthripragada, Mark Luciano

#### Department of Neurology, The Johns Hopkins Hospital, Baltimore, Maryland, United States

##### **Correspondence:** Aruna Rao

*Fluids and Barriers of the CNS* 2018, **15(Suppl 2):**P20

**Background:** Intracerebral pressure (ICP) monitoring is widely used in the intensive care setting for the management of traumatic brain injury. There are also elective uses of ICP monitoring when objective measurement of cerebrospinal fluid (CSF) pressure is required to make informed decisions about management whether it be CSF leak repairs, blood patches or adjustments in shunted patients.

Postural orthostatic tachycardia syndrome (POTS) is a disorder of the autonomic nervous system primarily affecting the control blood pressure and heart rate regulation. The hallmark of the disorder is the development of symptoms in the upright position and are relieved in the supine position which is similar to and can mimic cerebrospinal fluid (CSF) hypotension. The latter is a common indication for continuous intracerebral pressure (ICP) monitoring.

**Objective:** To determine the percentage of patients who are admitted for continuous intracerebral pressure monitoring that have normal intracerebral pressure monitoring but abnormal positional tachycardia consistent with positional orthostatic tachycardia syndrome or POTS.

**Methods:** This was a retrospective study in patients who were admitted for continuous ICP monitoring. We reviewed the charts of 130 patients admitted for continuous ICP monitoring between the years of 2015 and 2017. We measured ICP using Codman ICP express monitor which measures ICP using a strain gauge microchip located at the tip of the catheter. The micro sensor in inserted via a skull bolt by the neurosurgeon. ICP is transmitted as electrical voltage from the proximal end through nylon encapsulated copper wires which are connected to the Codman express monitor which displays the ICP measurement. We connect the Codman Express to a bedside monitor to obtain pressure waveforms using We converted the raw monitoring data into digital output format in order to analyze it. We converted the raw monitoring data into digital output format in order to analyze it. ICP is recorded in 60 s intervals and reported in mm of mercury (Hg). Other parameters are recorded using finger plethysmography include heart rate in beats per minute, blood pressure in mm of hg, oxygen saturation in percentage. ICP and heart rate was measured in supine position, sitting and standing position with the range of ICP reported as minimum, maximum and average values. Each position was maintained for a minimum of 10 min and average of 30 min before measurements were recorded. The average heart rate measurement was recorded in the same manner.

**Results:** Data from a total of 130 patients was collected. 83 were female, ages ranged from 16 to 68 years. Indication for ICP monitoring was presumed CSF hypotension in 60 patients (46%), presumed CSF hypertension in 49 patients (37%) and other indications including congenital hydrocephalus, shunt malfunction made up the rest. Of the CSF hypotension group 13/60 (21%) had normal intracerebral pressures but positional tachycardia of 30 beat increase in heart rate from supine to standing position consistent with POTS.

**Conclusion:** Our study demonstrates that obtaining objective measurement of continuous ICP with contiguous measurement of heart rate is important in diagnosing other causes of positional neurological symptoms that could mimic CSF hypotension. In our sample we were able to identify 21% of patients of POTS which were mistakenly thought to have CSF hypotension. This is an important distinction as the course of treatment for these two conditions is very different and could have valuable impact on patient care.

**References**

1. Agarwal AK, Garg R, Ritch A, Sarkar P. Positional orthostatic tachycardia syndrome. Postgrad Med J. 2007;83(981):478–80.
2. User manual for codman ICP monitoring systems; 2001.
3. Raboel PH, Bartek J, Andresen M, Bellander BM, Romner B. Intracranial pressure monitoring: invasive versus non-invasive methods—a review. Crit Care Res Pract. 2012;2012:950393.

## P21 A pilot study to assess gait and cognitive measurement devices for prediction of treatment response in patients with idiopathic normal pressure hydrocephalus

### James S. Samarasekara^1^, Fiona Evans^1^, Heinke Pulhorn^1^, Mark Lewis^2^

#### ^1^Department of Neurosurgery, Leeds General Infirmary, United Kingdom; ^2^Department of Neurology & Neuroscience, Leeds General Infirmary, United Kingdom

##### **Correspondence:** James S. Samarasekara

*Fluids and Barriers of the CNS* 2018, **15(Suppl 2):**P21

**Introduction:** Permanent shunting remains a potentially curative option for Idiopathic normal pressure hydrocephalus (iNPH), although up to 30% of patients do not see symptomatic benefit post procedure. We present a study protocol to examine the use of specialized gait/balance and cognitive measurement systems to better predict patients’ response to shunt treatment.

**Methods:** The gait device is a 3D measurement system that allows real-time analysis of physical movement properties. The cognitive device uses brain critical flicker frequencies to determine potential cognitive impairment. Our design is a single-group, before-and-after pilot/feasibility study which will run over the course of 1 year. A minimum of 20 patients with iNPH who undergo permanent shunting will be recruited. Demographic and patient outcome data will be collected pre and post treatment. Device testing will occur before and after initial diagnostic CSF drainage and after permanent shunt insertion. In identified tasks, correlation, specificity, sensitivity, positive-predictive and negative-predictive values will be identified. Parametric and non-parametric tests will be used to calculate task differences with a 0.05 significance level.

**Results:** The primary outcome is the identification of device tasks that are associated with symptomatic improvement after shunting. Secondary outcomes are to: identify task cutoff’s, identify the predictive values of relevant tasks, obtain data distributions and identify pragmatic issues with the study.

**Conclusions:** This is the first clinical trial to test currently available gait and cognitive measurement systems in patients with iNPH. We welcome feedback on this protocol and hope to identify parameters to better predict treatment response in iNPH patients.

## P22 Pitfall while changing ceratas valve pressure after lumbo-peritoneal shunt

### Kenji Sampei^1^, Chia-Cheng Chang^2^, Yoshihito Sakata^1^

#### ^1^Department of Neurosurgery, Nishi-Yokohama International General Hospital, Yokohama, Japan; ^2^Iyemasa Neurosurgical Clinic, Yokohama, Japan

##### **Correspondence:** Chia-Cheng Chang

*Fluids and Barriers of the CNS* 2018, **15(Suppl 2):**P22

**Introduction:** When lumbo-peritoneal shunt is performed using CERATAS Shunt System, it is controversial issue whether the programmable valve should be set in the ventral side or the dorsal side. In the cases whose valves are set in the ventral side, sometimes it is difficult to change valve pressure blindly. We examined the cause of these troubles and how to deal with this problem.

**Methods:** In four cases it was difficult to change the programmable pressure of the valves that were implanted in the ventral subcutaneous space. One case was thin and three had extra belly fat. In all cases the depth in the subcutaneous pressure valve was recognized not in excess of 1 cm by ultrasound echo. Therefore, we observed three-dimensionally the positioning of the implanted valve under fluoroscopic control.

**Results:** Shunt revision was needed for one who was only a thin case. In this case, we could not change the pressure for a reason that the bottom of the valve did not parallel to the adjustable magnet, which this valve was fixed firmly with a tissue under the skin. The valves of the other three cases were unstable in the belly fat tissue but the pressure could be changed with stretching the skin around the valve and/or pressing it and/or picking up the valve itself.

**Conclusions:** In case that it is not able to change the pressure with the adjunctive magnet, we should adjust it with some manipulation action by the hand under fluoroscopic guidance.

## P23 Effects of hyperbaric oxygen therapy in secondary lesions to experimental hydrocephalus associated with surgical treatment

### Stephanya C. Silva^1^, Omar Feres^1^, Pâmella S. Beggiora^1^, Hélio R. Machado^1^, Rafael M. Reis^2^, João E. Araújo^3^, Luíza S. Lopes^1^

#### ^1^Department of Surgery and Anatomy, Ribeirão Preto Medical School, University of São Paulo, Brazil; ^2^Department of Biomechanics, Medicine and Rehabilitation, Ribeirão Preto Medical School, University of São Paulo, Brazil; ^3^Department of Biomechanics, Medicine and Rehabilitation of the Locomotor System, Ribeirão Preto Medical School, University of São Paulo, Brazil

##### **Correspondence:** Stephanya C. Silva

*Fluids and Barriers of the CNS* 2018, **15(Suppl 2):**P23

**Introduction**: Hydrocephalus represents the accumulation of cerebrospinal fluid (CSF) in the cerebral ventricles. The increase of the CSF promotes various damages to the nervous tissue with cerebral hypoxia/ischemia the most important factor involved. Hyperbaric oxygen therapy (HBOT) promotes the improvement of O_2_ supply to tissues, and associated with surgical treatment, may further reduce the damage caused by hydrocephalus.

**Methods**: Seven-day-old Wistar Hannover rats were submitted to hydrocephalus by intracisternal injection of 10% kaolin in the cisterna magna. Were divided into six groups: control (n = 10), control with HBOT (3ATA/2 h/day) (n = 10), untreated hydrocephalic (n = 10), hydrocephalus with shunt (n = 10), hydrocephalus with shunt plus HBOT (n = 10). HBOT sessions was performed for 21 days. Behavioral tests (open field, Morris aquatic labyrinth, object recognition and activity monitor) and imaging exam by cranial ultrasonography were performed. After 21 days, the animals were euthanized, and the brain removed for histological and immunohistochemical studies.

**Results**: The hyperbaric treatment associated with CSF surgery appears to reduce ventricular enlargement, as seen by ultrasound examinations. The animals improved the behavioral performance (p = 0.0001), greater agility and greater exploration of the environment (p = 0.0001). They also presented greater preservation of spatial memory and learning capacity. Astrocytic activity was significantly reduced (p = 0.0001), highlighted by GFAP immunolabeling.

**Conclusions**: The results suggest that the hyperbaric treatment associated with the surgery improved the behavioral performance, offered benefits to the structures affected by the ventricular increase and a decrease of the ventricular size was observed. Thus, HBOT can be considered an adjuvant therapy for the surgical treatment of hydrocephalus.

## P24 Development of novel pulsatile diffuse correlation spectroscopy (DCS) indices for evaluating infant hydrocephalus

### Sutin D. B. Jason^1^, Chuan-Heng Hsiao^1^, Sarah Blackwell^1^, Francesca Yi^1^, Christopher Ha^1^, Brock Chimileski^1^, Benjamin C. Warf^1,2^, Pei-Yi Lin^1^, Ellen P. Grant^1^

#### ^1^Fetal-Neonatal Neuroimaging and Developmental Science Center, Boston Children’s Hospital and Harvard Medical School, Massachusetts, United States; ^2^Departments of Neurosurgery and Global Health and Social Medicine, Boston Children’s Hospital and Harvard Medical School, Massachusetts, United States

##### **Correspondence:** Sutin DB Jason

*Fluids and Barriers of the CNS* 2018, **15(Suppl 2):**P24

**Introduction:** Increases in mean intracranial pressure and intraventricular CSF pulsatility during hydrocephalus are suspected of causing progressive ventriculomegaly in infants. Investigations into the role of CSF pulsatility in treatment failure have been limited by the difficulty of measuring CSF dynamics. Cerebral blood flow (CBF) pulsatility is an alternative to CSF pulsatility which can be measured non-invasively. CBF pulsatility (PI) and resistive indices (RI) determined by Doppler ultrasound weakly correlate with hydrocephalus conditions, making them imperfect proxies for CSF pulsatility. We propose a new method to measure microvascular CBF pulsatility diffuse correlation spectroscopy (DCS). We hypothesize flow and pulsatile indices measured by DCS in the microvasculature may have different sensitivity to hydrocephalus progression than the corresponding Doppler ultrasound indices determined from blood flow in large cerebral arteries.

**Methods:** We have developed methods for fast DCS measurements of pulsatile CBF at rates up to 50 Hz in hydrocephalus infants. We enrolled three subjects of less than 3 months old with symptoms of ventriculomegaly or hydrocephalus and performed 14 measurements before and after treatment during their hospital stay. CBF PI and RI indices were computed from the DCS measurement of pulsatile CBF.

**Results:** We have demonstrated DCS measurements of mean and pulsatile CBF is feasible in infants with a resolution of up to five harmonics of the cardiac cycle. Work is ongoing to measure pulsatility in a larger cohort of infants.

**Conclusion:** DCS measurement of pulsatile CBF is a new development and we are exploring its potential as a diagnostic for hydrocephalus.

## P25 Simulations of combined broadband and frequency-domain near infrared spectroscopy for measurement of cerebral tissue water content during hydrocephalus

### Jacob Tatz^1^, Yvette Zou^1^, Chuan-Heng Hsiao^1^, Leticia Siqueira^1^, Cynthia Laurentys^1^, Benjamin Warf C.^2^, Ellen Patricia Grant^1^, Pei-Yi Lin^1^, Jason Sutin D. B.^1^

#### ^1^Fetal-Neonatal Neuroimaging and Developmental Science Center, Boston Children’s Hospital and Harvard Medical School, Massachusetts, United States; ^2^Departments of Neurosurgery and Global Health and Social Medicine, Boston Children’s Hospital and Harvard Medical School, Massachusetts, United States

##### **Correspondence**: Sutin Jason

*Fluids and Barriers of the CNS* 2018, **15(Suppl 2):**P25

**Introduction:** Frequency domain near-infrared spectroscopy (FDNIRS) and diffuse correlation spectroscopy (DCS) are optical techniques to measure cerebral tissue properties non-invasively. Using FDNIRS-DCS, we previously found hydrocephalus impairs cerebral blood flow (CBF) and oxygen metabolism (CMRO_2_) in infants. Furthermore, changes in the tissue optical scattering coefficient after surgery were found to be predictive of the likelihood of future treatment failure. The origin of this change is unclear but is suspected to be from changes in the distribution of water in the tissue, which is a significant component of the pathogenic mechanisms of hydrocephalus. We performed simulations to investigate the source of the signal and develop a new device to directly measure tissue water content by extending FDNIRS with broadband continuous wave NIRS covering the near-infrared water absorption band.

**Methods:** We simulated models of hydrocephalus using MCX, an open source software for GPU-based Monte Carlo simulation of photon fluence in tissue. We simulated conditions representing normal brains and those observed in hydrocephalus patients, including thin cortex from severe ventriculomegaly, extra-axial accumulation of cerebrospinal fluid (CSF), and cerebral edema.

**Results:** We compared the simulation results with measured data from hydrocephalus infants. We investigated the effect of different water distributions under the various conditions to determine the relationship between the pathological tissue properties and NIRS signals.

**Conclusion:** Combining broadband NIRS with FDNIRS-DCS is feasible and has potential for measuring changes in cerebral water transport underlying hydrocephalus.

## P26 Risk factors for phh among extremely premature infants with severe IVH: a penut ancillary study

### Hannah M. Tully^1,5^, Kerry Hancuch^2^, Christopher Traudt^3,4^, Bryan Comstock^2^, Daniel Doherty^4,5^, Sandra Juul^3,5^

#### ^1^Division of Pediatric Neurology, University of Washington/Seattle Children’s Hospital, Seattle, Washington, United States; ^2^Department of Biomedical Statistics, University of Washington, Seattle, United States; ^3^Division of Neonatology, University of Washington, Seattle, United States; ^4^Department of Pediatrics, University of Washington/Seattle Children’s Hospital, Seattle, United States; ^5^Center for Integrative Brain Research, Seattle Children’s Research Institute, Seattle, United States

##### **Correspondence:** Hannah M. Tully

*Fluids and Barriers of the CNS* 2018, **15(Suppl 2):**P26

**Introduction:** Among premature infants, post-hemorrhagic hydrocephalus (PHH) is a common consequence of severe intraventricular hemorrhage (IVH), yet little is known about why only some infants with severe IVH develop PHH. We sought to assess intrinsic and potentially modifiable risk factors associated with risk of PHH among a cohort of infants with severe IVH.

**Methods:** Retrospective cohort study using data prospectively collected part-of the multi-site PENUT clinical trial from infants born at 24–27 weeks’ gestational age. Demographic and perinatal clinical variables were analyzed by logistic regression to detect factors associated with the subsequent development of PHH.

**Results:** Among the 940 premature infants participating in the PENUT study, 130 experienced severe IVH, 82 (63%) of whom subsequently developed PHH. Compared to infants whose ventricular dilatation resolved, those who developed PHH were more likely to be male (OR: 2.4, 95% CI 1.1, 5.3) and to have received chest compressions during initial resuscitation: (OR: 2.9, 95% CI 1.2, 6.7). Each additional week of gestational age was associated with a 30% reduced risk of PHH (OR: 0.7, 95% CI 0.5, 0.95). 90% of infants received steroids prior to delivery, which was not associated with reduced risk of PHH. 80% of infants were delivered by C-section, which was not associated with reduced risk compared to vaginal delivery.

**Conclusions:** PHH remains a common complication of severe prematurity-associated hemorrhage. Among infants with Grade III/IV IVH, male sex, chest compressions and lower gestational age were associated with increased risk of PHH. Receipt of steroids prior to birth and C-section delivery were not associated with reduced risk of PHH.

